# Partially Randomized, Non-Blinded Trial of DNA and MVA Therapeutic Vaccines Based on Hepatitis B Virus Surface Protein for Chronic HBV Infection

**DOI:** 10.1371/journal.pone.0014626

**Published:** 2011-02-15

**Authors:** James S. Cavenaugh, Dorka Awi, Maimuna Mendy, Adrian V. S. Hill, Hilton Whittle, Samuel J. McConkey

**Affiliations:** 1 Medical Research Council Laboratories, Banjul, The Gambia; 2 Centre for Clinical Vaccinology and Tropical Medicine, University of Oxford, Oxford, United Kingdom; 3 David H. Smith Center for Vaccine Biology and Immunology, School of Medicine and Dentistry, University of Rochester, Rochester, New York, United States of America; 4 Institute of Maternal and Child Health, University of Port Harcourt, Post Harcourt, Nigeria; 5 International Agency for Research on Cancer, Lyon, France; 6 The Wellcome Trust Centre for Human Genetics, Oxford, United Kingdom; 7 Department of International Health and Tropical Medicine, Royal College of Surgeons in Ireland, Dublin, Ireland; Queensland Institute of Medical Research, Australia

## Abstract

**Background:**

Chronic HBV infects 350 million people causing cancer and liver failure. We aimed to assess the safety and efficacy of plasmid DNA (pSG2.HBs) vaccine, followed by recombinant modified vaccinia virus Ankara (MVA.HBs), encoding the surface antigen of HBV as therapy for chronic HBV. A secondary goal was to characterize the immune responses.

**Methods:**

Firstly 32 HBV e antigen negative (eAg^–^) participants were randomly assigned to one of four groups: to receive vaccines alone, lamivudine (3TC) alone, both, or neither. Later 16 eAg^+^ volunteers in two groups received either 3TC alone or both 3TC and vaccines. Finally, 12 eAg^–^ and 12 eAg^+^ subjects were enrolled into higher-dose treatment groups. Healthy but chronically HBV-infected males between the ages of 15 – 25 who lived in the western part of The Gambia were eligible. Participants in some groups received 1 mg or 2 mg of pSG2.HBs intramuscularly twice followed by 5×10^7^ pfu or 1.5×10^8^ pfu of MVA.HBs intradermally at 3-weekly intervals with or without concomitant 3TC for 11–14 weeks. Intradermal rabies vaccine was administered to a negative control group. Safety was assessed clinically and biochemically. The primary measure of efficacy was a quantitative PCR assay of plasma HBV. Immunity was assessed by IFN-γ ELISpot and intracellular cytokine staining.

**Results:**

Mild local and systemic adverse events were observed following the vaccines. A small shiny scar was observed in some cases after MVA.HBs. There were no significant changes in AST or ALT. HBeAg was lost in one participant in the higher-dose group. As expected, the 3TC therapy reduced viraemia levels during therapy, but the prime-boost vaccine regimen did not reduce the viraemia. The immune responses were variable. The majority of IFN-γ was made by antigen non-specific CD16^+^ cells (both CD3^+^ and CD3^–^).

**Conclusions:**

The vaccines were well tolerated but did not control HBV infection.

**Trial Registration:**

ISRCTN ISRCTN67270384

## Introduction

Hepatitis B virus (HBV) is a noncytopathic, hepatotropic DNA virus that can cause acute or chronic hepatitis (reviewed in [1], [2], [3], [4], [5], [6], [7], [8]). An effective preventative vaccine is available [9], [10], [11], however chronic HBV infection remains a serious public health burden in 5 to 10% of the world population, causing slightly over 50% of the cases of primary liver cancer worldwide [12], [13], [14]. Therapeutic vaccination could offer a curative treatment option. Two important questions arise for immunotherapy: what kind of immune response is needed? What epitopes or antigens should comprise the vaccine?

### Immune response to HBV

The immune response to HBV infection is complex and poorly understood in several important aspects. The antibody response is first to the core antigen (HBcAg) which does not predict control of the virus. HBV infection is clinically heterogeneous, ranging from completely asymptomatic to fatal, fulminant hepatitis, or to chronic liver failure, cirrhosis or hepatocellular carcinoma. There is no simple, quantitative relationship between the level of viraemia and the presence or severity of symptoms [15]. Nevertheless a meta-analysis concluded that there are statistically significant correlations between viraemia and histologic grading and biochemical and serological response [16]. The immune system is essential for HBV clearance [7], [17], [18]. The desired end point of therapy ought to be elimination of detectable viraemia [16].

### Effector mechanisms

Resolution of HBV infection is associated with vigorous and polyclonal HBV-specific CTL [19] activity directed against multiple HBV epitopes in the viral nucleocapsid, envelope and polymerase proteins [20], [21], whereas the CTL response is weak or absent in chronic carriers [22], [23]. The impaired T-cell responses can be restored transiently by 3TC therapy [24], [25], [26], [27]. Non-cytolytic mechanisms of viral control are expected on theoretical grounds [28] and are essential in a chimpanzee model [29], [30]. Similar results were subsequently shown in humans in a single-source outbreak [31]. Interferon-γ plays a key role in the clearance of HBV from chimpanzees' livers [30]. Studies with transgenic mice expressing HBV have demonstrated the importance of type I interferons (α, β) [32], [33], type II interferons (IFN-γ) [32], and type III interferons (IFN-λ) [34] as mechanisms for noncytolytic control. Most of the antiviral effect of CD8^+^ CTLs was shown to be mediated by IFN-γ [35]. Consequently, we used a cellular assay for IFN-γ as the primary measure of immune function in this study.

### Heterologous immunization for a CTL response

In animal models a CTL response can be elicited with DNA vaccination (reviewed in [36], [37]). DNA vaccination of humans has been reported for malarial antigens [38]. Mancini-Bourgine et al. reported the induction or expansion of T cell responses in humans after only DNA immunization with 0.5 mg of a DNA vaccine encoding the preS2 and S subunits of the HBV envelope protein in uninfected and in chronic HBV-infected people [39], [40]. Heterologous immunization, in which boosting for one antigen is done sequentially using different vectors, has been shown to be more effective than DNA immunization alone [41], [42], [43]. MVA's excellent safety profile and immunogenic properties make it a promising human vaccine candidate [44]. A prime-boost strategy using DNA followed by MVA has been used in several other studies and shown to be highly immunogenic for the induction of CD4^+^ and CD8^+^ T cells [45], [46], [47], [48], [49], [50]. In a murine malaria model, DNA immunization followed by recombinant MVA boosting induced a protective CTL response, whereas the vaccines in reverse order was not, nor was either of the vaccines by themselves [49]. These initial studies in mice have been extended to clinical trials. In a malaria vaccine study in The Gambia strong CD4 and weak CD8 T cell responses were induced by two 1 mg doses of a DNA vaccine given intramuscularly, followed by one dose of 3.0×10^7^ pfu (plaque forming units) MVA vaccine given intradermally at intervals 3 weeks apart [51]. Increasing the dose of the DNA vaccine to 2 mg and the MVA vaccine to 1.5×10^8^ pfu increased the effector T cell frequencies [52]. Dramatic loss of HBV viraemia was seen in a chronically infected chimpanzee after priming with a DNA immunization followed by boosting with a recombinant canarypox booster [53]. Taken together, these exciting results suggested that DNA priming with an HBV antigen followed by boosting with recombinant MVA expressing the same antigen could be a good choice for a therapeutic vaccine.

### Which antigen to use, and why?

The HBV genome is small, consisting of only 4 overlapping open reading frames. These encode 7 proteins: the large (L or pre-S1 + PreS2 + S), middle (sometimes “medium”) (M or pre-S2 + S), and small (S) surface antigens, the core (c) and pre-core (e) antigens (respectively known as HBcAg and HBeAg), the X antigen (so named because its function was initially enigmatic), and the viral polymerase. The antigenicities of these proteins differ; the core antigen is a very potent antigen by both a T cell dependent and a T cell independent mechanism [54] and is important for cellular immunity. The HBV S antigen (HBsAg), which is associated with viral adhesion, is also a very potent and reliable immunogen when assessed by antibody production. Neutralizing anti-HBs antibodies confer protection against future HBV infection, and all of the highly efficacious HBV prophylactic vaccines to date use HBsAg [11]. The excellent safety record with HBsAg was the primary motivation in choosing the middle surface protein (281 aa) from HBV genotype D as the antigen for vaccination in this study.

## Methods

### Objectives

The aim of this work was to determine if a heterologous therapeutic vaccination regimen was safe and effective in HBeAg negative and positive chronic HBV carriers. Change in viraemia by PCR was the main efficacy endpoint and sero-reversion the secondary one. The cellular immune response was measured by IFN-γ secretion in an ELISpot assay. Regarding safety, we already had some supportive safety data from pilot studies in UK and The Gambia on these vaccines (unpublished results).

The protocol for this trial and supporting CONSORT checklist are available as supporting information; see Checklist S1, Protocol S1, Protocol S2 and Protocol S3.

### Participants

Potential study participants were identified from databases of chronic hepatitis B carriers [9], [55], [56] from the Medical Research Council (MRC) Laboratories, Fajara, or from a local health centre. Males age 15 to 25 years who had HBV surface antigen (HBsAg) present in blood for over 6 months were eligible. The upper limit was chosen to avoid enrolling people who previously had vaccinia vaccination. Most had been positive since early childhood. Prospective volunteers had a baseline health screen. Those with significant illness, relevant allergy or ALT level over 88 IU/L were excluded.

Before enrollment into the study, potential candidates and members of their family were informed about the study in group meetings led by field workers in their first language (Wollof, Mandinka, or Fula). Each received an information sheet and consent form to take home, ponder, and discuss with family elders. Written informed consent was obtained for each person who enrolled. Parental written informed consent was obtained for those aged 15 to 18 years. Participants were not offered monetary compensation but were given transportation costs, a hot lunch and football video entertainment on study visit days, and free health care at MRC clinic during and for up to 6 months after the study ended.

The study documents and the recruitment and consent processes were reviewed by the joint Gambian Government/Medical Research Council Ethics Committee (http://www.saavi.org.za/inventory.htm#14) and the Central Oxford Research Ethics Committee (http://www.admin.ox.ac.uk/curec/). The clinical trial was monitored by an external group.

### Materials

Plasmid pSG2.HBs was generated by insertion of a gene fragment containing the *pre-S2* and *S* genotype D sequences of HBV strain *ayw* (the most common serotype in The Gambia) into the polylinker cloning region of vector pSG2. It contains the human cytomegalovirus (hCMV) immediate early promoter with intron A for driving expression of the HBsAg in mammalian cells, followed by the bovine growth hormone transcription termination sequence. The plasmid also contains a kanamycin resistance gene and is capable of replication in *Escherichia coli* but not in mammalian cells.

MVA.HBs contains the gene fragment with the same *pre-S2* and *S* sequences driven by the vaccinia P7.5 early/late promoter inserted into the thymidine kinase locus of MVA. It also contains the vaccinia late promoter P11 driving expression of the *lacZ* marker gene. MVA.HBs is produced in chicken embryo fibroblast cells. These were produced under Good Manufacturing Practice (GMP) conditions and donated by Oxxon Therapeutics (Oxford, UK). They were shipped to The Gambia on solid CO_2_ and stored at -70°C.

Rabies vaccine (Rabies Vaccine BP, Wistar rabies strain PM/WI 38 1503-3M, Human Diploid Cell Culture, Aventis Pasteur MSD) was stored lyophilized at 8°C until reconstituted following the manufacturer's instructions.

## Interventions

**Figure 1:**
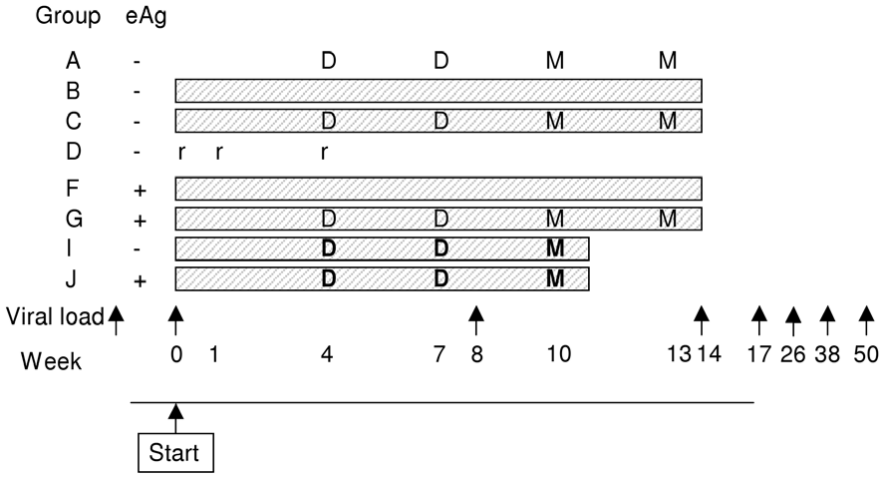
Time line illustrating group interventions. D = 1 mg pSG2.HBs. **D** = 2 mg pSG2.HBs. M = 5×10^7^ pfu MVA.HBs. **M** = 1.5×10^8^ pfu MVA.HBs. r =  rabies vaccination. eAg =  HBV e antigen. shaded block indicates lamivudine therapy. Groups I and J had 3-week earlier follow-up assays.

Figure 1 shows a time line for interventions. In the first phase four groups (A, B, C, D) of 8 HBsAg-positive, HBeAg-negative volunteers were recruited and allocated randomly. Those in groups A and C received 1 mg of pSG2.HBs intramuscularly twice at three weeks apart, which were then followed three weeks later by two doses of 5×10^7^ plaque forming units (pfu) of MVA.HBs (100 µL) intradermally, also three weeks apart. Those in groups B and C received oral 3TC therapy (100 mg daily; Zeffix®, GlaxoSmithKline, Greenford, Middlesex, United Kingdom) for 14 weeks, starting 28 days before vaccination. Those in the negative control group D received 0.1 mL (2.5 IU) of rabies vaccine intradermally on days 0, 7, and 28 (see Tables 1 and 2).

**Table 1:** Dosages for treatment groups.

Group	Assigned	*n*	HBsAg	HBeAg	pSG2.HBs	MVA.HBs	Lamivudine
A	8	7	+	-	1 mg (2×)	5×10^7^ pfu (2×)	
B	8	8	+	-			100 mg
C	8	9	+	-	1 mg (2×)	5×10^7^ pfu (2×)	100 mg
D	8	7	+	-			
F	8	7	+	+			100 mg
G	8	6	+	+	1 mg (2×)	5×10^7^ pfu (2×)	100 mg
I	12	7	+	+	2 mg (2×)	1.5×10^8^ pfu	100 mg
J	12	11	+	-	2 mg (2×)	1.5×10^8^ pfu	

**Figure 2:**
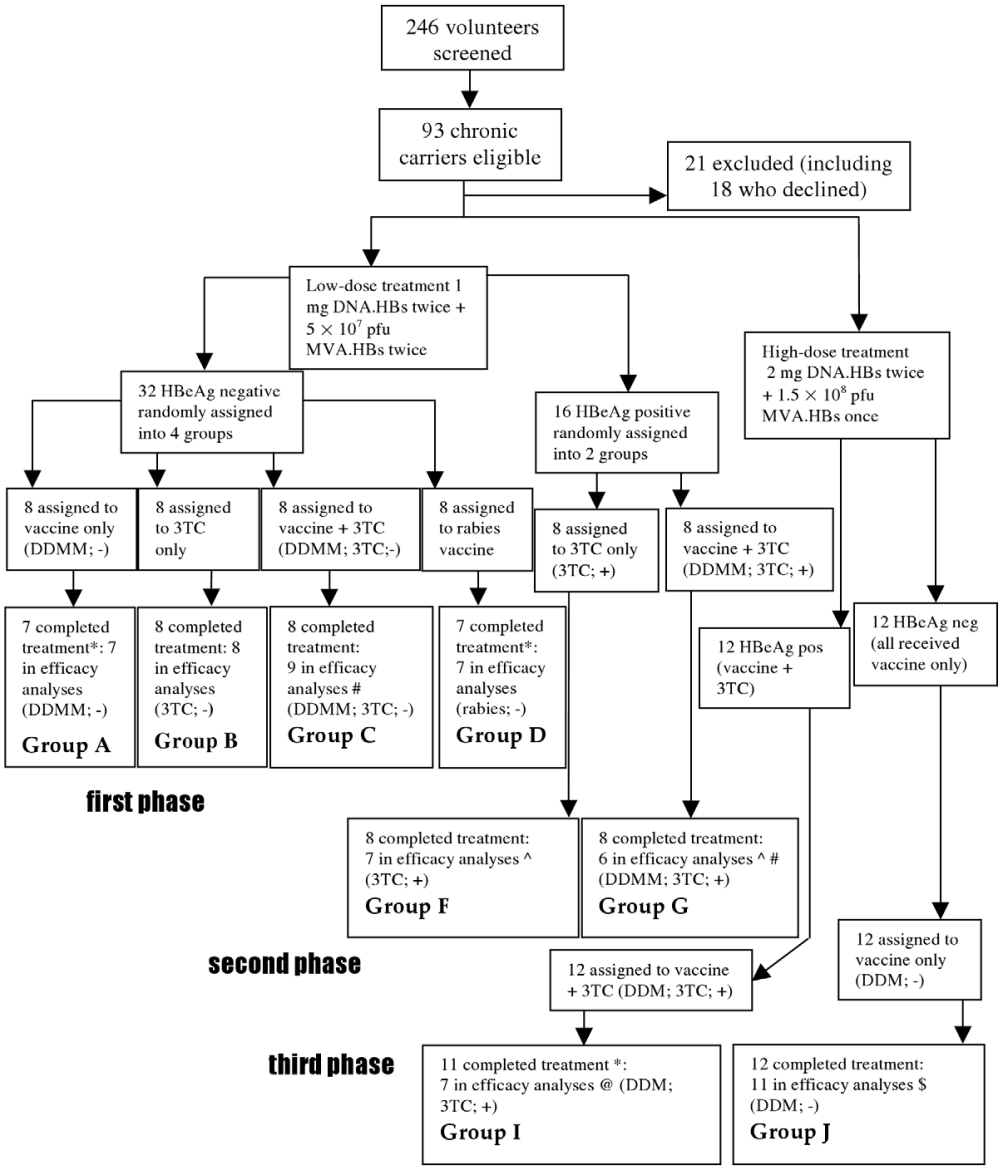
Flowchart showing the number of participants at each stage in the study. D =  pSG2.HBs; M =  MVA.HBs; 3TC =  lamivudine. * One participant in each of these groups declined to participate early in the study. The details are in the Results section. # One HBeAg negative participant was included in the VL analysis for DDMM; 3TC; - group who had been assigned in error to DDMM; 3TC; + group. ∧ One participant in each of these groups was discovered to have been HBsAg and HBeAg negative all through the study and did not meet eligibility criteria, due to a manual transcription error. @ Re-analysis of samples from four participants showed they had HBeAg negative chronic HBV from before the beginning of the intervention and thus did not meet eligibility criteria for this group. $ Re-analysis of samples from one participant showed he had HBeAg positive chronic HBV from the beginning of the intervention and thus did not meet eligibility criteria for this group.

• 2× indicates that the vaccine was administered twice.

• Vaccinations were separated by a 3-week interval.

• In the relevant groups, lamivudine was commenced 4 weeks before administration of the first vaccination and it was used for 14 weeks except for members of Group I, who used it for 11 weeks.

• *n* is the number of subjects in the efficacy analyses, not the number of subjects initially assigned to that group (see Figure 2 and related discussion).

**Table 2:** Time categories for analysis, in days.

Group	Pre-treatment	Treatment	Post-treatment	Follow-up
A	≤28	30–91	93–119	>119
B	≤0	3–98	119	>119
C	≤0	3–98	119	>119
D	≤0	7, 28	56	>56
F	≤0	3–98	119	>119
G	≤0	3–98	119	>119
I	≤0	3–77	98	>98
J	≤28	30–70	77–98	>98

In the second phase, two groups (F, G) of 8 HBeAg-positive volunteers each received 14 weeks of 3TC as described above. Group G also received the pSG2.HBs and MVA.HBs vaccines as described above. Consequently groups B and F were equivalent (received 3TC only) except for eAg status, and likewise C and G.

When the favorable safety and disappointing efficacy results were available from the groups described above, higher doses of vaccines were used in phase three, in two further non-randomized study groups (I, J). We planned to enroll 12 HBeAg-positive HBV carriers into Group I to receive 11 weeks of 3TC therapy and beginning at day 28 to receive 2 mg pSG2.HBs intramuscularly on two occasions, followed by one dose of 1.5×10^8^ pfu MVA.HBs (3 intradermal injections of 100 µL each), all 3 weeks apart. We planned to enroll 12 HBeAg-negative HBV carriers into Group J to receive the same vaccination regimen but without lamivudine. These changes in dose and regimen were based on results from trials of similar malaria vaccines. Table 1 summarizes the treatment interventions for each group.

Volunteers were observed for one hour after vaccination and were visited at home by trained field workers on the following second, fourth and seventh days to assess vital signs, local adverse events (discoloration, induration, blister formation, pain, limitation of arm motion, scar and other reactions), systemic adverse events (headache, nausea, malaise, axillary temperature) and to record other unsolicited adverse events.

One week after each vaccination and at 4, 13, 25 and 37 weeks after the last vaccination venous blood was collected for measurement of full blood count, urea, creatinine and liver enzymes (AST, ALT, γ-GT). For the serology and viral load assays, venous blood was collected 1 week after the second vaccine and at 1, 4, 13, 25 and 37 weeks after the last vaccine. Deviations from protocol times of up to 5 days were tolerated, but uncommon.

### Outcomes measures

#### HBV assays

Samples were tested for HBsAg by reverse passive hemagglutination assay (Wellcotest®, Murex Diagnostics, Dartford, UK) and later by Determine™ HBsAg (Abbott Laboratories, Illinois, USA), an immunochromatographic assay. Samples were tested for HBeAg using an enzyme immunoassay (Equipar Diagnostici, Saronno(Va), Italy). The plasma HBV viral load was measured initially by an outsourced laboratory (Covance) using Roche Amplicor qPCR. Later we developed and validated our own competitive real-time quantitative PCR as described elsewhere [57]. The limits of detection and quantification were about 40 and 260 copies mL^−1^ respectively. Because DNA was used as an immunogen, anti-DNA antibodies were measured by a standard assay in the Clinical Immunology Department, The Churchill, Oxford Radcliffe Hospital, Oxford.

#### Ex vivo ELISpot

Fresh *ex vivo* interferon-γ ELISpot assays were performed by adding 380,000 peripheral blood mononuclear cells (PBMCs) from heparinized fresh whole blood to each well of a quarter of a 96-well Millipore MultiScreen™ plate MAIPS4510 (Millipore, Billerica, Massachusetts, USA), along with the appropriate stimulant for that well, to a final volume of 100 µL and incubated overnight in a 37°C incubator with 5% CO_2_ in air. The cells were stimulated either with RN10 medium alone (i.e., RPMI 1640 [Sigma-Aldrich R 8758, St. Louis MO], penicillin and streptomycin [98 U mL^−1^], L-glutamine [1.96 mM] and 10% human heat-treated AB serum), with overlapping pools of peptides spanning the HBV middle surface protein (15-mers overlapping by 5 amino acids), or with a positive control (FEC [a mixture of 22 known HLA Class I restricted peptides from influenza, CMV and EBV], PHA, or PPD [tuberculin purified protein derivative]). The sequences of the peptides matched that in the vaccines exactly and are described in File S1. The ELISpot plates were coated with capture antibody (1-D1K, Mabtech, Stockholm, Sweden) overnight at 8°C and blocked with R10 (i.e., as RN10 but substituting fetal bovine serum for human) for 1 hour prior to the ELISpot assay. After overnight incubation, the ELISpot plates were emptied and washed with PBS-Tween. The tracer antibody (7-B6-1, Mabtech, Stockholm, Sweden) was added for 2 h to overnight at 8°C. The developed plates were read on an automated plate reader (Autoimmun Diagnostika GmbH, Strassberg, Germany) and manually edited and double checked to remove clearly artifactual marks from being counted as spots. The count settings and similar details are further described in File S1. These data were exported from the AID plate reader electronically as Microsoft Excel files which were imported into a Microsoft Access 2000 database for data management, presentation and analysis as described elsewhere [58]. Queries were designed to exclude data from unacceptable or suspicious wells.

#### Flow cytometry analysis

Intracellular cytokine staining (ICCS) was used to establish the phenotype of the IFN-γ producing cells from subjects in groups I and J. PBMCs, either freshly isolated by Lymphoprep™ (Axis-Shield, Oslo, Norway) density centrifugation or from previously frozen samples, were washed and then stimulated with the overlapping pools of HBsAg peptides, or with medium alone, or with a positive control (either FEC, PHA, or PPD) for at least 6 h, in accordance with BD Biosciences' recommendations for IFN-γ staining [59]. Brefeldin A (Sigma) was added at least 4 h before removal from the incubator and staining. Cells were washed and then 0.5 mL of FACS Permeabilizing Solution 2 (BD Biosciences) was added to each tube for 15 min prior to dilution with 3 mL of PBS. The cells were then stained with pre-mixed panels of antibody stains for 30-60 min. They were washed and then stored in approximately 200 µL of 4% formalin in PBS at 8°C until data acquisition on a BD FACSCalibur 4-color instrument (BD Biosciences). Cells passing through lymphocyte gates (both small and large lymphocytes on an SSC vs. FSC plot) were batch analyzed with FCS version 2.0 (De Novo Software) to generate Excel files, which were then imported into a Microsoft Access database for data management [58].

### Sample size

For the initial studies, a total of 32 subjects (8 per group) was considered a minimum number in order to meet the study objectives of assessing preliminary safety of the vaccines and determining its efficacy at reducing HBV DNA levels based on data about stability of HBV viral load in eAg positive subjects. Experience with antiviral agents and with vaccines in general suggested that a relatively large effect size might be expected if the treatment were successful. If heterologous prime-boost were to behave in humans as it has been seen in rodents and non-human primates, then 8 per group would be adequate to find this effect [50]. After gaining experience with likely numbers of dropouts and measurement variability, the sample size for groups I and J was increased to 12 to make it likely that data from at least 10 subjects would be available at the end of the study.

### Randomization

Initially 32 HBsAg-positive, HBeAg–negative volunteers were block randomized by the investigators using a table of random numbers to one of 4 groups: A, B, C, D. The randomization was performed after the decisions for enrollment had been made by the participant and communicated to the study field workers and physicians.

### Blinding

Because the primary and secondary end points of the study were laboratory measurements (qPCR and ELISpot) of blood samples with minimal opportunity for conscious or subconscious subjective bias, we felt that the benefit of blinding would be outweighed by its logistic difficulties, so no attempt at blinding was made.

### Statistical methods

#### Data management

Three relational databases were developed in Microsoft Access 2000: one for immunological (ELISpot and flow cytometry) data [58], one for clinical data, and one for virological (qPCR) data. The clinical data were double entered and discrepancies were identified using a tool developed at MRC for this purpose and corrected. Considerable care was given to the accuracy of the data.

#### Model fitting

Exploratory analysis of our immunological data used a mixed effects model. Initially we tried to fit the data for all volunteers to a cubic model. The variability within groups was high and there were no significant interactions. We then put the data into meaningful time categories from which repeated measures ANOVA with correlation between times was done. For efficacy analyses we did paired *t* tests before treatment and after treatment for each group using the time categories shown in Table 2. Group comparisons for categorical data were performed with Fisher's exact test. All calculated *p* values were 2-tailed. All results and participants were included in the safety analysis. Efficacy analyses were based on treatments received. Exploratory analyses (pairs plots) were done in R to see the overall correlation between all the laboratory values.

## Results

### Recruitment

Two hundred forty six volunteers were screened for eligibility between January 2002 and December 2003, of whom 153 were HBsAg-negative and hence ineligible. Of the remaining 93 HBsAg-positive volunteers, 18 volunteers declined to participate for personal reasons, probably related to the amount of visits and phlebotomy, and 3 were excluded: 2 because of sickle cell disease, and 1 lost HBsAg before the start of the study. Thus, 72 people were eligible, enrolled in the trial and were allocated to one of the 8 groups. Of these, 69 completed their treatment. One volunteer in group A dropped out after the first vaccination. One each in groups D and I declined after 4 weeks participation. No reasons were given for this. They were excluded from the efficacy analysis, but their results relevant to the safety of the interventions are presented. Figure 2 shows details of the treatment allocation and the reasons for not progressing in the study. The baseline characteristics of the participants in the different treatment arms are shown in Table 3.

**Table 3:** Baseline characteristics of volunteers: age, viraemia, and liver inflammation (mean ± SD).

Group	*n*	Age	log_10_ Viraemia	Range of log_10_ Viraemia	AST	ALT
A	7	20.5±4.2	2.9±1.9	5.9	26±4.0	19±7.0
B	8	16.3±2.2	3.3±2.0	6.6	23±10	23±7.3
C	9	17.6±3.5	2.9±2.2	6.4	28±6.5	19±3.8
D	7	18.8±2.9	2.3±2.2	5.5	30±7.6	17±8.2
F	7	17.6±3.4	9.2±0.6	2.1	39±22	28±23
G	6	16.2±2.6	8.7±0.5	1.3	41±11	20±10
I	7	17.7±2.5	8.8±0.9	2.0	72±50	81±61
J	11	20.6±2.4	4.2±0.6	2	22±7.8	15±10

At the end of the study we found that two HBsAg –negative participants had been enrolled in violation of the protocol. We then re-tested baseline screening samples and found that 6 volunteers had incorrect HBeAg determination then. In one case this was due to a borderline result, in two cases due to spontaneous loss of HBeAg in the period between the screening assay and the beginning of the study interventions, and in three cases to communication errors. Because the interventions and monitoring in group G were identical to those in group C, we reallocated the participant from group G to group C for the efficacy analyses. This made it possible for this person's results to be analyzed with the group that they should have been in, had the assignment been made correctly at the outset. The results from the other 5 participants (4 in group I and 1 in group J) were not included in the efficacy analysis but are included as safety data.

### Lamivudine compliance

Adherence as assessed by pill count was quite good: 11/43 had 100% compliance; 26/43 had 95–99% compliance; 4/43 had 90–94% compliance, and 2/43 had <90% compliance.

### Outcomes and estimation: safety

#### Clinical laboratory variables

Exploratory analyses of the laboratory results are provided in File S2. Overall ALT levels correlated more strongly with viraemia than did AST (Pearson correlation coefficients of 0.361 and 0.326 respectively), and overall ALT correlated strongly (as expected) with overall AST (Pearson correlation 0.788); see Ancillary Analyses below. The kinetics for the other biochemical data are shown in the File S3. No particularly striking changes were seen in ALT, AST, γ-GT, or haemoglobin; these varied about as much in the treatment groups as in the controls. The serum creatinine was elevated in groups A and C participants around the time of the MVA injections, and in group B around the time of the viral rebound. The variability was comparable across all groups. No anti-DNA antibodies were detected in any of the people who received pSG2.HBs. The dataset may be found in Dataset S1.

### Adverse events

#### Solicited systemic adverse events

In general the vaccines were safe and well tolerated. There were few systemic adverse events after the DNA and MVA vaccines at both doses as shown in Table 4. Most of these adverse events were mild, that is, they did not interfere with activities of daily living.

**Table 4:** Frequency of adverse events after each dose of MVA vaccine. Numbers in parentheses indicate the percentage of vaccine recipients in that group that reported each adverse event.

Adverse events	First dose DNA^♣^ *n* = 47	2^nd^ dose DNA^♣^ *n* = 46	MVA 1(5×10^7^ pfu)*n* = 23^♠^	MVA 2 after MVA 1(5×10^7^ pfu)*n* = 23^♠^	MVA(1.5×10^8^ pfu)*n* = 23^♥^
Tenderness	0	0	8 (34.8%)	10 (43.4%)	7 (30.4%)
Redness	0	0	17 (73.9%)	11 (47.8%)	17 (73.9%)
Hardness	0	1	23 (100%)	23 (100%)	23 (100%)
Scaling	0	0	23 (100%)	17 (73.9%)	23 (100%)
Shiny plaque	0	1	0	4 (17.4%)	22 (95.7%)
Fever	0	0	1 (4.3%)	0	0
Diarrhea	0	0	0	2 (8.6%)	0
Fatigue	2	4	4 (17.4%)	3 (13.0%)	1 (4.3%)
Body ache	2	5	9 (39.1%)	4 (17.4%)	0

♣ There were no unsolicited adverse events after DNA vaccination in groups I or J.

♠ 23 = 7+9+6+1 for groups A, C, G, and 1 of group exclude respectively (or alternatively, 7+8+8 for group A and the original allocations for groups C and G).

♥ 23 = 7+11+4+1 for groups I, J, I-originally, and J-originally respectively.

#### DNA vaccine (pSG2.HBs)

A total of 47 doses of 1 mg pSG2.HBs and 46 doses of 2 mg pSG2.HBs were given. Hardness at the vaccination site (of 2 mm diameter which resolved in 2 days) was noted in one participant and a temporary pigmented mark was noted in one other. These were graded mild by the investigators. After the administration of 1 mg pSG2.HBs 5 participants reported episodes of fatigue and body ache. The timing of these suggested to the investigators that these were unrelated to the vaccination. No systemic or local adverse events were recorded after 46 administrations of 2 mg pSG2.HBs.

#### MVA.HBs

A total of 46 doses of 5×10^7^ pfu MVA.HBs and 23 doses of 1.5×10^8^ pfu MVA.HBs were given. The vaccines were well tolerated at the different doses with mild and moderate adverse events documented (Table 4). No changes outside of the normal ranges were observed in the vital signs during 1 h post-vaccination. An episode of mild diarrhoea and one of mild fever were reported which resolved without treatment within 2 or 3 days respectively. Painful lymphadenopathy was found in one person in the first week after the first dose of 5×10^7^ pfu MVA.HBs vaccination. A 1.5 cm right axillary lymph node was palpated ipsilateral to the vaccination site in the skin over the deltoid muscle though there were no other abnormal symptoms or signs and no restriction of arm movements. By day 10 the swelling had resolved.

**Figure 3:**
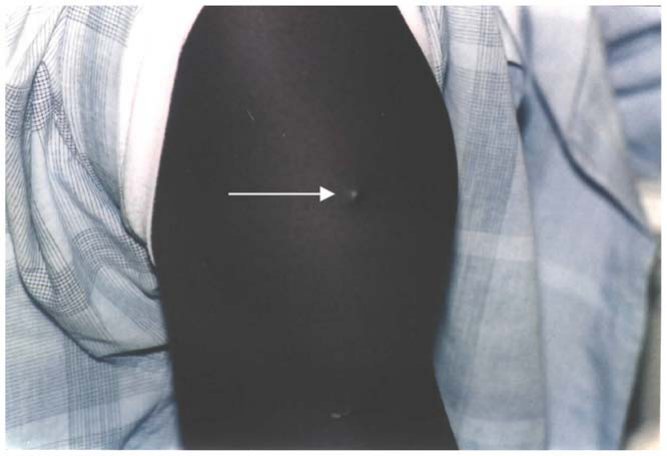
Typical shiny plaque seen at site of HBs.MVA injection on right shoulder. The skin is over the right deltoid muscle of a participant showing the vaccination site 129 days after 1.5×10^8^ pfu MVA.HBs administration by intradermal injection of 0.1 mL at each of 3 sites. An arrow highlights the small shiny pigmented macule seen at one of these sites.

A characteristic local reaction was observed after administration of MVA.HBs. After the intradermal injection, a small vesicle developed at the site, signifying correct intradermal injection technique. This disappeared within 30 minutes of vaccination. Induration developed during the first 2 days after vaccination, in most cases, non-tender. There was no limitation of arm movement. Subsequently, redness, induration and scaling were observed on the 2^nd^ day post-vaccination which developed to maximal size by the 4^th^ to 7^th^ day post-vaccination and gradually disappeared, leaving a shiny plaque scar of 3 to 5 mm diameter by the 28^th^ day post-vaccination as shown in the photograph in Figure 3. This developed by 4 weeks post-vaccination in approximately 1/2 of the cases and by 5 weeks in approximately 3/4 of the cases, the remaining cases taking up to 14 weeks to appear. The maximal diameter of the redness, induration and scaling varied from 2 mm to 13 mm, 0.5 mm to 15 mm and 0.3 mm to 12 mm respectively. These were similar for both dose regimens of MVA.HBs. However, a significantly higher proportion of volunteers who received 1.5×10^8^ pfu of MVA (three injections of 5×10^7^ pfu at once) had shiny plaque scars compared with those who received two injections of 5×10^7^ pfu of MVA on opposite shoulders three weeks apart (22/23 versus 4/23 individuals, *p* value = 7.3×10^−8^). The shiny plaques persisted beyond the end of the study: final observations ranged from day 245 to day 337. Giving three MVA.HBs injections to one individual at a time may increase the probability that at any one injection site a shiny plaque will develop (22/69 versus 4/46 injections, *p* value  = 3.3×10^−3^).

#### Unsolicited adverse events

Numerous unsolicited adverse events in vaccines and in control volunteers were recorded as shown in File S4. The most common unsolicited adverse events were headaches (50), anaemia (37), likely related to malaria, and malaria (33), which is endemic in The Gambia. Abdominal pain (27), fever (25), and cough (20) were also common complaints. Two adverse events happened that required hospitalization for treatment: an episode of malaria in one patient in the low-dose vaccine treatment group and an episode of moderate anaemia with pyrexia in one patient in the higher dose vaccine treatment group. Both episodes occurred 6–9 months after vaccination and were unrelated to the therapy.

### Outcomes and estimation: efficacy

#### HBV serology

None of the participants in any group lost HBsAg during the study period. One of seven HBeAg-positive participants in group I had lost HBeAg by day 63 of the protocol by which time he had received lamivudine 100 mg daily for 9 weeks and two administrations of 2 mg pSG2.HBs intramuscularly on days 28 and 49. During the study the HBV viral load for this participant also declined from 7.8 to 5.3 log_10_ copies mL^−1^. No other HBeAg-positive participant changed their serological status during the study.

#### Viral load

**Figure 4:**
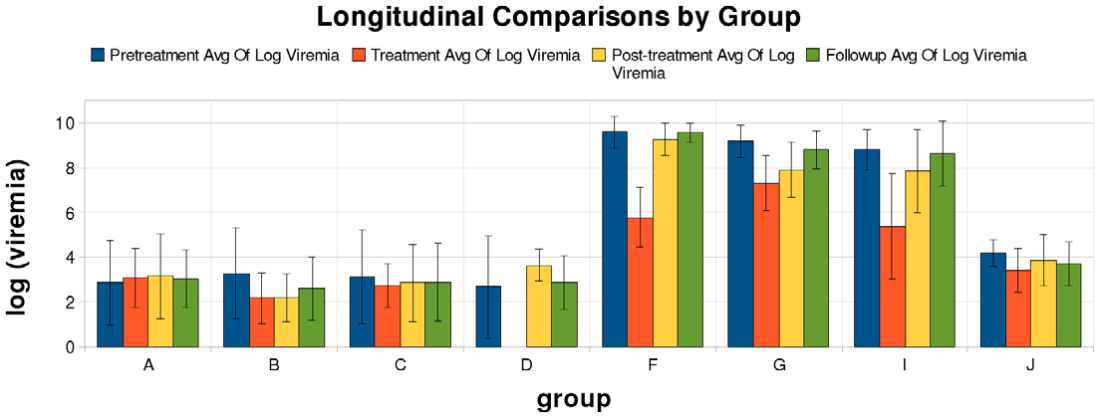
Groups compared directly and by treatment interval. This figure shows the average value of log_10_ (viraemia) for each group with error bars representing standard deviation. Sensible comparisons include longitudinal comparisons for each group as well as comparisons designed to test specific components of the therapy. For example, if one considers only groups A and D, one could infer treatment effects due to the vaccine. The comparisons for which one might infer therapeutic efficacy are shown in Table 7.

None of the vaccination regimens had a noticeable sustained effect on the HBV viral load (Figure 4). Individuals' viral kinetics are shown in File S3. Table 5 lists *p* values for before versus after comparisons within groups by treatment interval. As expected, most participants who received lamivudine had up to a 4 log_10_ decrease in HBV DNA viral copies mL^−1^ below their pretreatment levels. The HBeAg-negative and HBeAg-positive people who received lamivudine therapy had respective geometric means of 2.9 and 9.3 log_10_ copies mL^−1^ at baseline and 2.6 and 6.3 log_10_ copies mL^−1^ at end of lamivudine treatment. The decline in viraemia was most striking in the HBeAg positive groups who had high initial viral load. By three weeks after discontinuation of lamivudine there was a rebound in viral load back to the pretreatment values. The fluctuations that exist are very likely indicative of the natural course of HBV infection and the HBV DNA levels in the control arm (Group D) shows as much variation as in any of the other groups, except those taking lamivudine.

**Table 5:** Paired 2-tailed *t* test *p* values within each group for viraemia data.

Group	*n*	Treatment vs Pre-treatment	Post-treatment vs Pre-treatment	Follow-up vs Pre-treatment
A	7	1.53×10^−1^ (1.00)	4.22×10^−1^ (1.00)	7.97×10^−1^ (1.00)
B	8	1.34×10^−1^ (1.00)	9.80×10^−2^ (1.00)	1.04×10^−1^ (1.00)
C	9	6.58×10^−1^ (1.00)	9.01×10^−1^ (1.00)	9.97×10^−1^ (1.00)
D	7		8.87×10^−2^ (1.00)	3.96×10^−1^ (1.00)
F	7	2.32×10^−4^ (0.005)	3.24×10^−1^ (1.00)	9.17×10^−1^ (1.00)
G	6	1.01×10^−2^ (0.232)	3.52×10^−2^ (0.810)	2.62×10^−1^ (1.00)
I	7	3.13×10^−3^ (0.072)	2.28×10^−1^ (1.00)	9.78×10^−1^ (1.00)
J	11	7.24×10^−2^ (1.00)	3.64×10^−1^ (1.00)	1.37×10^−1^ (1.00)

Since a vaccine is intended to provide immunological memory, the most important comparison is the follow-up vs. pre-treatment, although one could also make a case for post-treatment vs. pre-treatment. Values compared were averages for each subject during the time interval, computed using the database software. Sufficient power was present even in Group G, with only 6 members, to see a statistically significant effect during this interval. However, after Bonferroni correction for multiple hypothesis testing, only in Group F is significance maintained at the traditional 0.05 level. Values in parentheses are after Bonferroni correction for multiple hypothesis testing. In no case is there evidence for the efficacy of the vaccine regimen in lowering viraemia. (For the follow-up vs. pre-treatment comparison for Group J, *n* = 10 since one person was lost to follow-up.)

### Outcomes and estimation: immunogenicity

#### IFN-γ responses measured by ELISpot

**Figure 5:**
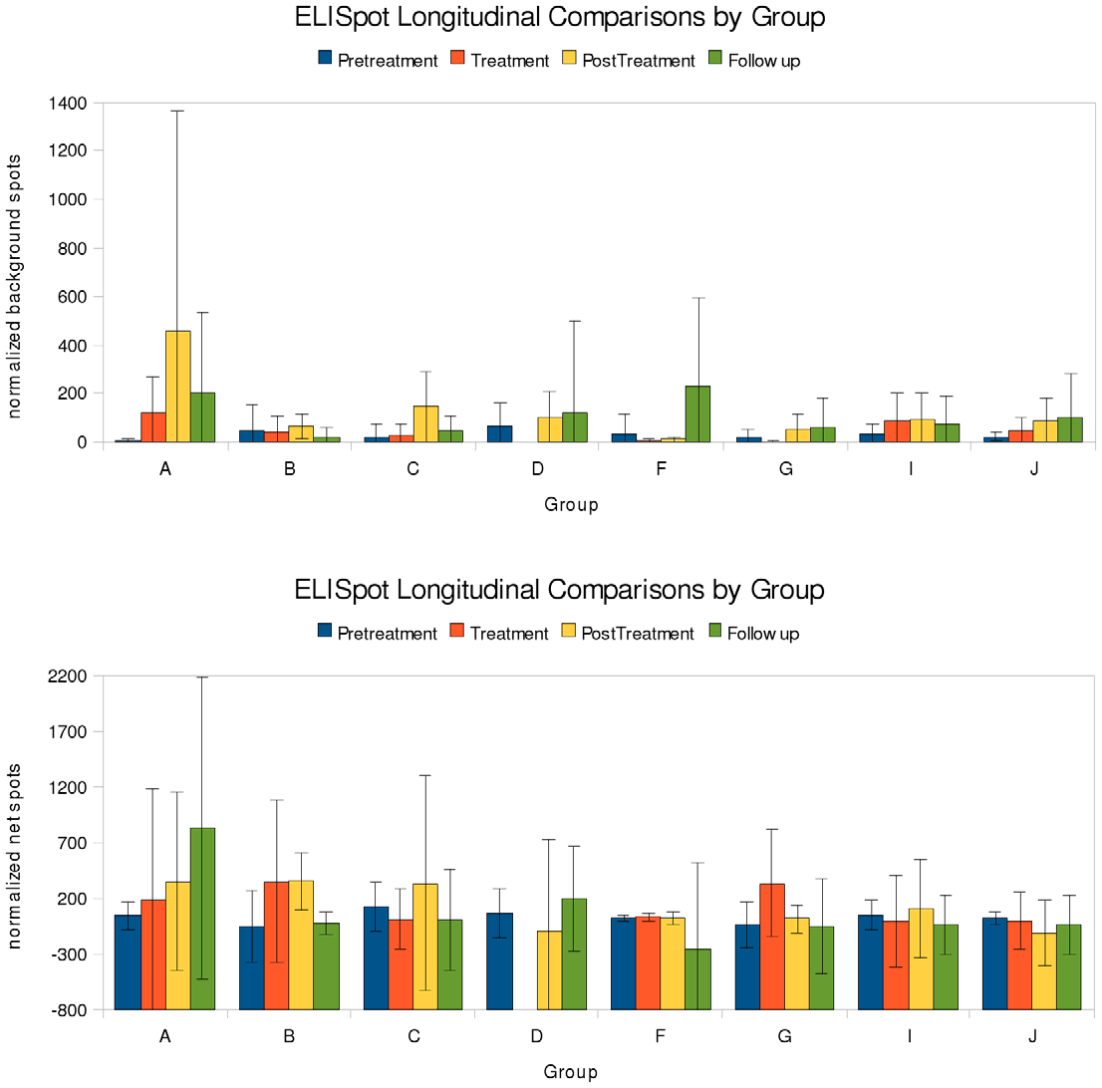
Background and net ELISpot results normalized to per million PBMCs. The net spots were calculated according to the following formula:
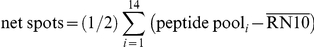
where RN10 is the average of the spots from the two negative control wells. The summation is over all 14 wells in the plate layout which contained overlapping pooled HBsAg peptides for each volunteer, and the factor of 1/2 normalizes for each peptide appearing twice in the matrix layout. Because of the 14 summations the effect of a slightly low or slightly high background (measured over only 2 wells) gets amplified in the final net spots count. The immune response would be expected to be strongest in the post-treatment time interval and to wane in the follow-up period, but in fact variability was often high in the follow-up period. This may reflect non-specific immune activation due to other maladies such as malaria or to the natural course of engaging a chronic HBV infection.

There was no strong evidence for vaccine-specific IFN-γ responses in any of the groups, although there was a small but discernable increase in background response in group C at day 119, four weeks after the last vaccination. Figure 5 shows the number of cells producing IFN-γ measured by ELISpot for the nonspecific (medium-only) and HBsAg-peptide stimulated cultures. As the size and clarity of spots can vary with cell type and assay conditions [60], figures in File S5 describe the quantitative amounts of IFN-γ produced. Table 6 shows associated *p* values for comparisons of IFN-γ producing cells. Further statistical comparisons of the number of spots and the amount of IFN-γ produced and putative epitopes and details about the ELISpot assay are described in File S6.

**Table 6:** Paired 2-tailed *t* test *p* values within each group for ELISpot data (normalized net spots).

Group	*n*	Treatment vs Pre-treatment	Post-treatment vs Pre-treatment	Follow-up vs Pre-treatment
A	7	7.33×10^−1^ (1.00)	2.43×10^−1^ (1.00)	9.42×10^−2^ (1.00)
B	8	1.74×10^−1^ (1.00)	6.89×10^−2^ (1.00)	8.54×10^−1^ (1.00)
C	9	3.59×10^−1^ (1.00)	5.43×10^−1^ (1.00)	4.77×10^−1^ (1.00)
D	7		5.46×10^−1^ (1.00)	7.54×10^−1^ (1.00)
F	7	3.57×10^−1^ (1.00)	7.97×10^−1^ (1.00)	2.73×10^−1^ (1.00)
G	6	9.09×10^−2^ (1.00)	5.98×10^−1^ (1.00)	9.53×10^−1^ (1.00)
I	7	7.69×10^−1^ (1.00)	7.88×10^−1^ (1.00)	3.75×10^−1^ (1.00)
J	11	7.36×10^−1^ (1.00)	1.45×10^−1^ (1.00)	3.01×10^−1^ (1.00)

Blood for ELISpot was not taken during the treatment period for groups B, F, and G. Values in parentheses are after Bonferroni correction for multiple hypothesis testing.

**Table 7:** Appropriate comparisons (companion table to Figure 4)

Comparison	eAg status	Experimental variable isolated
Group A vs Group C	negative	lamivudine
Group A vs Group D	“	vaccine
Group B vs Group C	“	vaccine
Group B vs Group D	“	lamivudine and rabies vaccine
Group C vs Group D	“	lamivudine, vaccine combination
Group A vs Group J	“	dose of vaccine
Group G vs Group F	positive	vaccine
Group F vs Group I	“	vaccine
Group G vs Group I	“	dose of vaccine

The background spots had moderate variability, except in group A, which was quite high. Group D controls showed as much variation as any other group.

#### Phenotyping of IFN-γ producing cells

Intracellular cytokine staining (ICCS) was used in groups I and J to identify the surface phenotype of the cells making IFN-γ. In these groups few IFN-γ producing cells were found using ICCS (consistent with the ELISpot results). Neither CD4^+^ nor CD8^+^ T cells made significant IFN-γ as assayed by ICCS. The time course of T cells (CD3^+^), probable NK cells (CD16^+^), and NKT cells (CD3^+^CD16^+^) that make IFN-γ is shown in File S3. Although few cells produced IFN-γ, the picture that emerges (more clearly in group J than I) is that the majority of IFN-γ production was made by antigen- nonspecific CD16^+^ cells, both CD3^+^ and CD3^–^, consistent with the ELISpot results.

### Ancillary analyses

#### Other results

All other analyses were exploratory. The HBV viral load result shown in Figure 4 suggested that there might be a difference in the responses during the treatment phase associated with the vaccine, specifically that the viral load in the vaccinated group who got lamivudine (group G) may have dropped less than in those who received lamivudine alone (group F). This difference is statistically significant before correction for multiple comparisons in a regression model, *p* value  = 0.014, but because 26 different such comparisons could have been performed it is not statistically significant after correction for multiple testing. However, the effect is interesting and it is biologically plausible that the immune response to vaccination could have increased viral replication. The study was underpowered to detect an interaction like this (Figure 4 and File S3).

Analysis also included pairs plots for the laboratory data and the corresponding correlation matrix. As one may expect, there are a fairly strong correlations between hemoglobin, red blood cell count, packed cell volume and mean corpuscular volume and between IFN-γ spot numbers and cytokine levels. See File S2 for details.

Following the negative efficacy and immunogenicity results, we transported leftover clinical vials of pSG2.HBs and MVA.HBs from Gambia back to UK and performed the murine stability and potency assays on the contents, which showed that they had not lost potency due to storage or transportation.

## Discussion

### Interpretation

#### Synopsis of key findings

We describe the safety, efficacy and immunogenicity of a new therapeutic vaccination regimen: priming with a DNA vaccine encoding the HBV surface protein and boosting with a recombinant poxvirus encoding the same antigen, in HBeAg-positive (generally high viraemia) and HBeAg-negative (generally low viraemia) healthy volunteers with chronic HBV, in some cases with concomitant lamivudine antiviral therapy. The vaccination regimens were well-tolerated but failed to achieve a reduction in HBV viraemia. Importantly, although there were a small number of volunteers in each treatment group, there was sufficient power to detect statistically significant effects during the treatment period, as demonstrated in the groups receiving lamivudine and as shown in Table 5. Also, as expected, this lamivudine-induced drop was greater in people with HBeAg-positive than in those with HBeAg-negative infection, as the latter began with markedly lower viral loads, and because the quantitative PCR assay performs less accurately at or near its limit of quantifiability.

There was high variability in net spots in fresh *ex vivo* IFN-γ ELISpot assays. The reasons why the background spots were so high are unknown, but the frequent bouts of malaria and other maladies which were reported as unsolicited adverse events may have caused temporary increases in nonspecific immune responses in the volunteers. ICCS in groups I and J suggested that NK and NKT cells produced IFN-γ in a peptide-nonspecific fashion after vaccination.

#### Possible mechanisms and explanations

The optimal dose of an immunogen is very difficult to predict [61]. Initially doses of 1 mg pSG2.HBS and 5×10^7^ pfu MVA.HBs were chosen. Later higher doses were used based on immunogenicity results of studies of similar malaria vaccines. That participants in the lower-dose vaccine group had 5 times more mild to moderate adverse events than those in the higher-dose group may be due to seasonal effects. Malaria was the most commonly observed unsolicited adverse event and is highly seasonal in The Gambia. Malaria season corresponded to the follow-up period for the lower-dose groups.

No effects were observed on transaminase levels, anti-HBe seroconversion, or HBsAg seroreversion after 9–11 months of follow up. One person lost HBeAg, but spontaneous loss of HBeAg occurs not infrequently, as demonstrated by the 2 participants who lost HBeAg in the interval between first screening and repeat baseline testing. The mean annual rate of spontaneous seroconversion has been estimated at 8% to 15% in individuals with active liver disease and 2% to 5% in those with normal ALT [6]. In another recent study we reported that 86% of HBV infected children in Gambia recruited between the ages of 1–4 years, lost HBeAg by the age of 19 years, compared to 30% who lost HBsAg [62].

One possible reason for vaccination failure is antigenic diversity. In Gambia there are two HBV genotypes: about 87% are genotype E, the rest A [55]. pSG2.HBS and MVA.HBs contain a genotype D sequence, which is 93% identical amino acids to genotype E [63]. It is unlikely that this significantly affected T cell responses. This is not a likely explanation for the failure of these vaccinations.

Another possible reason for the lack of efficacy is the profound immune tolerance which most infected persons in The Gambia have towards HBV. It is acquired in early childhood or at birth, in contrast to people in Europe who mostly acquire it as adults. Thus, the efficacy of immunotherapeutic agents may differ based on the epidemiology of the disease, associated with circumstances of acquisition and immune tolerance.

#### Comparison with other published studies

Other studies have assessed HBV vaccine therapy for chronic HBV infection [39], [64], [65], [66], [67]. The low efficacy found in this study contrasts with findings from some other studies which show that vaccine therapy in combination with antiviral drugs decreases HBV viral replication and HBV DNA to undetectable levels by inducing HBsAg-specific T-cells. Horiike et al. [66] describe intradermal administration of HBsAg protein with 1 year lamivudine therapy and found seroconversion from HBeAg to anti-HBe in 5 of 9 participants. However, that study was conducted in older people who may have acquired infection in adulthood and have elevated serum ALT levels, which may favor HBV control, in contrast to the young healthy chronic HBV carriers used in the present study. Dahmen et al. [65] show that 4 of 14 (28.6%) chronic HBV carriers with unfavorable prognostic factors, such as pre-core HBV mutants or previous interferon-α non-response, had viral clearance and biochemical responses when given HBV surface protein with alumimium hydroxide with lamivudine or interleukin-2 combination therapy. Yalcin et al. showed no significant effects on HBV levels, HBeAg to anti-HBe seroconversion or on transaminase levels following 3 intramuscular injections of a recombinant DNA vaccine also coding for HBsAg [68]. The variability seen between these studies may be due to variability in the populations and the stage of infection, different vaccines, frequency or route of administration and other factors.

There are favorable reports [39], [40] of using HBsAg in a DNA vaccine in chronically infected individuals. One difference between the studies is the number of DNA immunizations: four DNA immunizations with improvement seen after three immunizations, compared to two followed by MVA vaccines. This does not seem to be the sole explanation, however, in light of the human malaria DNA, MVA studies in which we showed very high levels of IFN-γ producing T cells (which were mostly CD4^+^ cells) [52]. The French group [39], [40] reporting the positive phase I trial result from DNA immunization alone used prolonged cultured ELISpot (for 2 weeks), whereas in the current study all of the ELISpots were ex vivo stimulated for less than 24 hours. The most important difference between the current study and that of Mancini-Bourgine [40] was that the current one included an untreated control group.

Only weak correlation (e.g., −0.117) was seen between any of the ELISpot immunogenicity measures and viraemia, in contrast to the strong correlation reported by Webster *et al.* using MHC-I tetramers instead of ELISpot [15]. Besides the assay differences (phenotypic marker versus functional assay), another possible reason for this discrepancy is that Webster *et al.* measured responses to core and polymerase proteins in addition to surface, which was the only one in this current study. Neither study found any association between markers of liver damage (AST, ALT) and cellular immune function, although we did find a weak association between markers of liver damage and viraemia. Thus, we conclude that HBV infection in Gambia is a heterogeneous condition which defies finding a relationship easily between viraemia and immune responses.

Strong net responses with low background spots, as seen in several cases in the *ex vivo* ELISpot results in this study, indicate an incomplete tolerance, and show that the ability to react to HBsAg specifically is still present in HBeAg negative HBV infected subjects. Suppressor T cells (also called regulatory T cells or Tregs) may modify the responses and have been shown to be important in mediating the immunosuppression characteristic of chronic HBV infections [69]. Regulation in immunology seems to have become synonymous with suppression, but activation and suppression are both forms of regulation. We prefer the original term (suppressor T cells) as more descriptive.

Recently results of some similar prime-boost vaccine trials have been published which were also disappointing [70], [71]. In contrast, another study reports that *in vitro* and in HLA transgenic mice a multiepitope heterologous prime-boost immunization with the plasmid DNA and a recombinant MVA worked as a therapeutic vaccine insofar as providing further enhancement of the immune responses [72]. However, they did not report any antiviral efficacy. Indeed, another vaccine trial that is similar to aspects of ours (in particular, the Group F versus Group G comparison, although with a different vaccine) also reported lack of efficacy of the therapeutic vaccine to reduce viraemia despite induction of a vigorous HBsAg-specific lymphoproliferative response [73]. Another earlier study [74] also reported a lack of efficacy of HBsAg for clearing the virus; the authors ascribed this to Th2 cytokines produced by HBsAg whereas they found Th1 cytokines produced by HBcAg. Indeed, in hindsight the short answer to our failure to generate an antiviral response may well be that we used the wrong antigen.

### Generalizability

#### Clinical implications

This study is longitudinal, dose-ranging, with eAg^+^ and eAg^–^ subjects across a wide range of viraemia, with and without concomitant lamivudine therapy, in a total of 8 arms. The consistent picture regarding efficacy that emerges from quantitative virological and immunological data is that pSG2.HBS and MVA.HBV are unable to break the profound tolerance of the immune system to HBsAg in HBV chronic carriers. It is likely that similar results would be seen in other populations including women, who were not included in this study. Likewise, expanding the age range considerably would probably not affect the results, although HBV is usually acquired at a very young age in The Gambia and the immune systems of very young children may make these results inapplicable to that population. The safety results of this study are also probably quite valid for a wider population, since (i) the DNA plasmid had such paltry immunogenicity itself, (ii) the HBV middle surface protein insert into MVA apparently did not radically increase its immunogenicity, and (iii) MVA was widely used in the final stages of the smallpox eradication campaign in Germany and has been well tolerated in many other studies. It is, however, noteworthy that the shiny plaques seen at the higher MVA.HBs dose were “completely missing” after MVA itself administered predominantly to participants with lightly or non-pigmented skin in Europe [44].

#### Research implications

Did our particular prime-boost vaccines fail because of the particular antigen chosen, the dosage (typically much higher in animals), or for some other reason? The most likely reason, we think, is that chronically infected people are profoundly immunotolerant towards the middle surface protein (M protein), which has been present in very high levels in blood and extracellular fluid since early childhood. In contrast, when given as a vaccine to non-infected people, it is very immunogenic and 2 doses of it in alum predictably lead to high levels of antibody. The HBV core protein may have been a better choice as it is strongly immunogenic by both T cell dependent and T cell independent mechanisms [54]. As pointed out by a reviewer, better responses might have been achieved by adding in ubiquitous T cell epitopes or possibly even slightly varying the HBsAg sequence (e.g., by 5–7% mismatches) to help break the tolerance. In the case of the 3TC-treated volunteers, a longer pre-treatment interval (8–12 weeks) might have allowed greater T cell recovery and possibly better results.

How can one break the immune tolerance induced by HBV? Because a decrease in viraemia (as for example during antiviral therapy) leads to increased T cell responsiveness, and that this is reversible, indicates that tolerance is actively maintained either directly or indirectly by the virus. There may be a role of suppressor T cells [75], [76]. There is evidence in mice that had been primed by DNA immunization that depleting suppressor T cells can enhance the CD8^+^ T cell response against HBV [77].

### Overall evidence

#### Limitations of the present study

The interpretations of the present study need to be limited by the fact that small or moderate sized effects cannot be excluded by this study design. The flow cytometry results in groups I and J indicated that most of the INF-γ producing cells were probably NK or NKT lymphocytes. One caveat to this is that there were very few gated cells making IFN-γ, and statistics with few events are less credible than with many events. However, these results are consistent with the few spots detected in ELISpot. This problem was exacerbated by the limited amount of blood taken, the variable recovery of PBMCs, and the fact that ELISpot had priority over ICCS for use of PBMCs. Furthermore, NK cells are not uniquely defined by CD16, and the CD16^lo^CD56^hi^ subset of NK cells has been identified as the subset that makes the most IFN-γ [78]. For these reasons we do not claim that the majority of the IFN-γ producing cells were definitely NK or NKT cells, only that the preponderance of evidence – including the ELISpot data and the fact that IFN-γ was also made with or without antigen (peptide) stimulation – indicates that this is likely.

Finally, this study was not blinded, but that does not seem to have been a problem given the laboratory nature of the data. We had no bias towards negative results; all the investigators were optimistic that the study would have demonstrated efficacy.

## Supporting Information

File S1 Supplementary Material: Methods.
(0.15 MB DOC)

File S2 Supplementary Material: Results of Exploratory Analyses.
(0.44 MB DOC)

File S3 Supplementary Material: Results of Individual Kinetics.
(0.19 MB DOC)

File S4 Supplementary Material: Results of Unsolicited Adverse Events.
(0.09 MB DOC)

File S5 Supplementary Material: Results of Epitope Screening.
(0.14 MB DOC)

File S6 Supplementary Material: Results of Statistical Significance of ELISpot.
(0.05 MB DOC)

Checklist S1 CONSORT checklist.
(0.23 MB DOC)

Protocol S1 Trial Protocol.
(0.44 MB DOC)

Protocol S2 Trial Protocol.
(0.41 MB DOC)

Protocol S3 Trial Protocol.
(0.27 MB DOC)

Dataset S1 Supplementary Material: Dataset.
(1.03 MB XLS)
