# Supplementary material for: Partially Randomized, Non-Blinded Trial of DNA and MVA Therapeutic Vaccines Based on Hepatitis B Virus Surface Protein for Chronic HBV Infection
Source: PLoS One. 2011 Feb 15;6(2):e14626. doi: 10.1371/journal.pone.0014626 (PMC3039644; doi:10.1371/journal.pone.0014626)
Supplement: File S1 — Supplementary Material: Methods. (0.15 MB DOC) [file pone.0014626.s001.doc]

**SUPPLEMENTARY MATERIAL: METHODS**

***ELISpot details***

***Peptides and peptide pools.*** The 46 peptides used for stimulations were combined into 14 overlapping pools of 7 peptides each (except the 7th and 14th pools which had 4 peptides). These peptides were 15-mers overlapping by 9 aa’s which spanned the HBV middle protein. Supplementary Table 1 shows the peptide pools for the ELISpot assays, and Supplementary Table 2 shows the sequences of the peptides used in ELISpot assays. Each peptide was present in 2 different pools as an aid to screening for which peptides were epitopes, similar to the layout optimization reported for HIV epitopes [1,2,3]. Peptides were synthesized by standard Fmoc chemistry by a laboratory that was outsourced by Oxxon Therapeutics and were sent to us by Oxxon Therapeutics.

***ELISpot count settings.***  ELISpot count settings are almost never reported but are absolutely critical to the results, so we would like to buck this trend. Although the best count settings varied occasionally from plate to plate, these were the most commonly used count settings with AID Elispot 3.0c software and worked well except when the background was very high: Minimum values: Intensity, 10; Size 1; Gradient 1 (Min); Basic Settings: Fuzz Filter OFF; Filter White Rings OFF; Emphasis SMALL; Sensitivity N/A; Background Enhancement: Dynamic Contrast Enhancement: OFF; Apply algorithm from versions 2.5 - 2.9: yes. These settings are sensitive and counted all spots, not only the large ones (which were rare).

***Net spots.*** The net spots were calculated according to the following formula:

net

where RN10 is the average of the spots from the two negative control wells. The summation is over all 14 wells and the factor of 1/2 normalizes for each peptide appearing twice in the matrix layout. We used the following exclusion criteria: (1) plates having extremely high background, (2) poor agreement between spots in the duplicate negative control wells, (3) many counts in the negative control wells but few counts in the peptide wells, (4) if they otherwise appeared too suspicious to accept without further confirmation, such as very few spots in all wells except for many spots in a well immediately adjacent to a positive control. Only wells that passed these criteria were included in the analyses.

**REFERENCES**

1. Addo MM, Yu XG, Rathod A, Cohen D, Eldridge RL, et al. (2003) Comprehensive Epitope Analysis of Human Immunodeficiency Virus Type 1 (HIV-1)-Specific T-Cell Responses Directed against the Entire Expressed HIV-1 Genome Demonstrate Broadly Directed Responses, but No Correlation to Viral Load. J Virol 77: 2081-2092.

2. Gillespie Geraldine MA, Pinheiro S, Sayeid-Al-Jamee M, Alabi A, Kaye S, et al. (2005) CD8^+^ T cell responses to human immunodeficiency viruses type 2 (HIV-2) and type 1 (HIV-1) gag proteins are distinguishable by magnitude and breadth but not cellular phenotype. European Journal of Immunology 35: 1445-1453.

3. Keating SM, Bollinger RC, Quinn TC, Jackson JB, Carruth LM (2002) Cross-Clade T Lymphocyte-Mediated Immunity to HIV Type 1: Implications for Vaccine Design and Immunodetection Assays. AIDS Research and Human Retroviruses 18: 1067-1079.

4. Lathey JL (2003) Preliminary Steps Toward Validating a Clinical Bioassay: A Case Study of the ELIspot Assay. BIOPHARM INTERNATIONAL 16: 42-51.

**Peptide pool**

**Pool 1**

**Pool 2**

**Pool 3**

**Pool 4**

**Pool 5**

**Pool 6**

**Pool 7**

**Pool 8**

**1**

**2**

**3**

**4**

**5**

**6**

**7**

**Pool 9**

**8**

**9**

**10**

**11**

**12**

**13**

**14**

**Pool 10**

**15**

**16**

**17**

**18**

**19**

**20**

**21**

**Pool 11**

**22**

**23**

**24***

**25**

**26**

**27**

**28**

**Pool 12**

**29**

**30**

**31**

**32**

**33**

**34**

**35**

**Pool 13**

**36**

**37**

**38**

**39**

**40**

**41**

**42**

**Pool 14**

**43**

**44**

**45**

**46**

**--**

**--**

**--**

**Supplementary Table 1. Pools of peptides.** Each peptide is represented in 2 pools (e.g., peptide 10 in pools 3 and 9).

* Peptide 24 was dropped in later assays (for Groups I, J) due to its low solubility.

| **Origin of sequence** | **Amino acid sequence** | **Origin of sequence** | **Amino acid sequence** |
| --- | --- | --- | --- |
| HBV pre-S2-S 1 | MQWNSTTFHQTLQDP | HBV pre-S2-S 35 | IPIPSWSAFGKFLWE |
| HBV pre-S2-S 2 | TFHQTLQDPRVRGLY | HBV pre-S2-S 36 | WAFGKFLWEWASARF |
| HBV pre-S2-S 3 | QDPRVRGLYFPAGGS | HBV pre-S2-S 37 | LWEWASARFSWLSLL |
| HBV pre-S2-S 4 | GLYFPAGGSSSGTVN | HBV pre-S2-S 38 | ARFSWLSLLVPFVQW |
| HBV pre-S2-S 5 | GGSSSGTVNPVLTTA | HBV pre-S2-S 39 | SLLVPFVQWFVGLSP |
| HBV pre-S2-S 6 | TVNPVLTTASPLSSI | HBV pre-S2-S 40 | VQWFVGLSPTVWLSV |
| HBV pre-S2-S 7 | TTASPLSSIFSRIGD | HBV pre-S2-S 41 | LSPTVWLSVIWMMWY |
| HBV pre-S2-S 8 | SSIFSRIGDPALNME | HBV pre-S2-S 42 | LSVIWMMWYWGPSLY |
| HBV pre-S2-S 9 | IGDPALNMENITSGF | HBV pre-S2-S 43 | MWYWGPSLYSILSPF |
| HBV pre-S2-S 10 | NMENITSGFLGPLLV | HBV pre-S2-S 44 | SLYSILSPFLPLLPI |
| HBV pre-S2-S 11 | SGFLGPLLVLQAGFF | HBV pre-S2-S 45 | SPFLPLLPIFFCLWV |
| HBV pre-S2-S 12 | LLVLQAGFFLLTRIL | HBV pre-S2-S 46 | FLPLLPIFFCLWVYI |
| HBV pre-S2-S 13 | GFFLLTRILTIPQSL | Flu peptide 1 | GILGFVFTL |
| HBV pre-S2-S 14 | RILTIPQSLDSWWTS | Flu peptide 2 | RVLSFIKGTK |
| HBV pre-S2-S 15 | QSLDSWWTSLNFLGG | Flu peptide 3 | ELRSRYWAI |
| HBV pre-S2-S 16 | WTSLNFLGGTTVCLG | Flu peptide 4 | SIIPSGPLK |
| HBV pre-S2-S 17 | LGGTTVCLGQNSQSP | Flu peptide 5 | LPFDKTTVM |
| HBV pre-S2-S 18 | CLGQNSQSPTSNHSP | Flu peptide 6 | ASCMGLIY |
| HBV pre-S2-S 19 | QSPTSNHSPTSCPPT | Flu peptide 7 | FMYSDFHFI |
| HBV pre-S2-S 20 | HSPTSCPPTCPGYRW | Flu peptide 8 | KTGGPIYKR |
| HBV pre-S2-S 21 | PPTCPGYRWMCLRRF | Flu peptide 9 | SRYWAIRTE |
| HBV pre-S2-S 22 | YRWMCLRRFIIFLFI | EBV peptide 1 | CLGGLLTMV |
| HBV pre-S2-S 23 | RRFIIFLFILLLCLI | EBV peptide 2 | DYCNVLNKEF |
| HBV pre-S2-S 24 | LFILLLCLIFLLVLL | EBV peptide 3 | QAKWRLQTL |
| HBV pre-S2-S 25 | CLIFLLVLLDYQGML | EBV peptide 4 | AVFDRKSDAK |
| HBV pre-S2-S 26 | VLLDYQGMLPVCPLI | EBV peptide 5 | FLRGRAYGL |
| HBV pre-S2-S 27 | GMLPVCPLIPGSSTT | EBV peptide 6 | YPLHEQHGM |
| HBV pre-S2-S 28 | PLIPGSSTTSTGPCR | EBV peptide 7 | GLCTLVAML |
| HBV pre-S2-S 29 | STTSTGPCRTCMTTA | EBV peptide 8 | RAKFKQLL |
| HBV pre-S2-S 30 | PCRTCMTTAQGTSMY | EBV peptide 9 | RRIYDLIEL |
| HBV pre-S2-S 31 | TTAQGTSMYPSCCCT | CMV peptide 1 | NLVPMVATV |
| HBV pre-S2-S 32 | SMYPSCCCTKPSDGN | CMV peptide 2 | SDEEEAIVAYTL |
| HBV pre-S2-S 33 | CCTKPSDGNCTCIPI | CMV peptide 3 | IPSINVHHY |
| HBV pre-S2-S 34 | DGNCTCIPIPSSWAF | CMV peptide 4 | TPRVTGGGAM |

**Supplementary Table 2.** Amino acid sequences of the peptides used in the immunological stimulation assays from HBV subtype *ayw*.
