# Supplementary material for: Partially Randomized, Non-Blinded Trial of DNA and MVA Therapeutic Vaccines Based on Hepatitis B Virus Surface Protein for Chronic HBV Infection
Source: PLoS One. 2011 Feb 15;6(2):e14626. doi: 10.1371/journal.pone.0014626 (PMC3039644; doi:10.1371/journal.pone.0014626)
Supplement: File S2 — Supplementary Material: Results of Exploratory Analyses. (0.44 MB DOC) [file pone.0014626.s002.doc]

**SUPPLEMENTARY MATERIAL: RESULTS of EXPLORATORY ANALYSIS**

## ***Exploratory statistics and data visualization***

We did some exploratory plots to detect very general trends and correlations on all the data and on selected subsets. Exploratory Figure 1 shows a pairs plot generated using the freely available open source software R [1] for the laboratory data. The corresponding Pearson correlation matrix is shown below in Exploratory Table 1 with entries highlighted for which the absolute value > 0.3. As expected, fairly strong overall correlations exist between AST and ALT; between hemoglobin, RBC (red blood cell count), PCV (packed cell volume), MCV (mean corpuscular volume), and (surprisingly less so) MCHC (mean corpuscular hemoglobin concentration); and between spots and cytokine (“spots” measuring number of cells producing IFN-γ, and “cytokine” measuring relative amount made). The strongest correlation was between the neutrophil count and the lymphocytes count (-0.919; 95% confidence intervals: -0.932, -0.905 against a null hypothesis of no correlation). There is a weak positive overall correlation between AST or ALT and LogViremia (0.326 and 0.361 respectively; for ALT and LogViremia the 95% confidence limits are 0.276 and 0.441). Note that in this analysis we include both the change in viremia as well as viremia itself, as the former may be more informative. Exploratory Figure 2 shows overall trends for some biochemical data.

In Exploratory Figure 1 there appears to be two subsets of donors: those for which a strong correlation exists between LogViremia and DeltaLogViremia, and those for which no such correlation exists. This difference is real and segregates naturally between the e antigen negative donors (groups A, B, C, D, I-originally, and J) and the e antigen positive donors (groups F, G, I, and J-originally), shown respectively in Exploratory Figures 3 and 4 respectively. The corresponding correlation matrices are listed as well. There are considerably more correlations in the e antigen positive subgroup. Although the values are still small in the e antigen positive subgroup, there is a correlation between the change in viremia and the AST level which is statistically significant:

**> cor.test(eAgpositive$DeltaLogViremia, eAgpositive$AST)**

Pearson's product-moment correlation

data: eAgpositive$DeltaLogViremia and eAgpositive$AST

t = 2.6653, df = 129, p-value = 0.008676

alternative hypothesis: true correlation is not equal to 0

95 percent confidence interval:

0.05925601 0.38490195

sample estimates:

cor

0.2284595

**REFERENCE**

1. R Development Core Team (2007). R: A language and environment for statistical computing. R Foundation for Statistical Computing, Vienna, Austria. ISBN 3-900051-07-0, URL http://www.R-project.org.


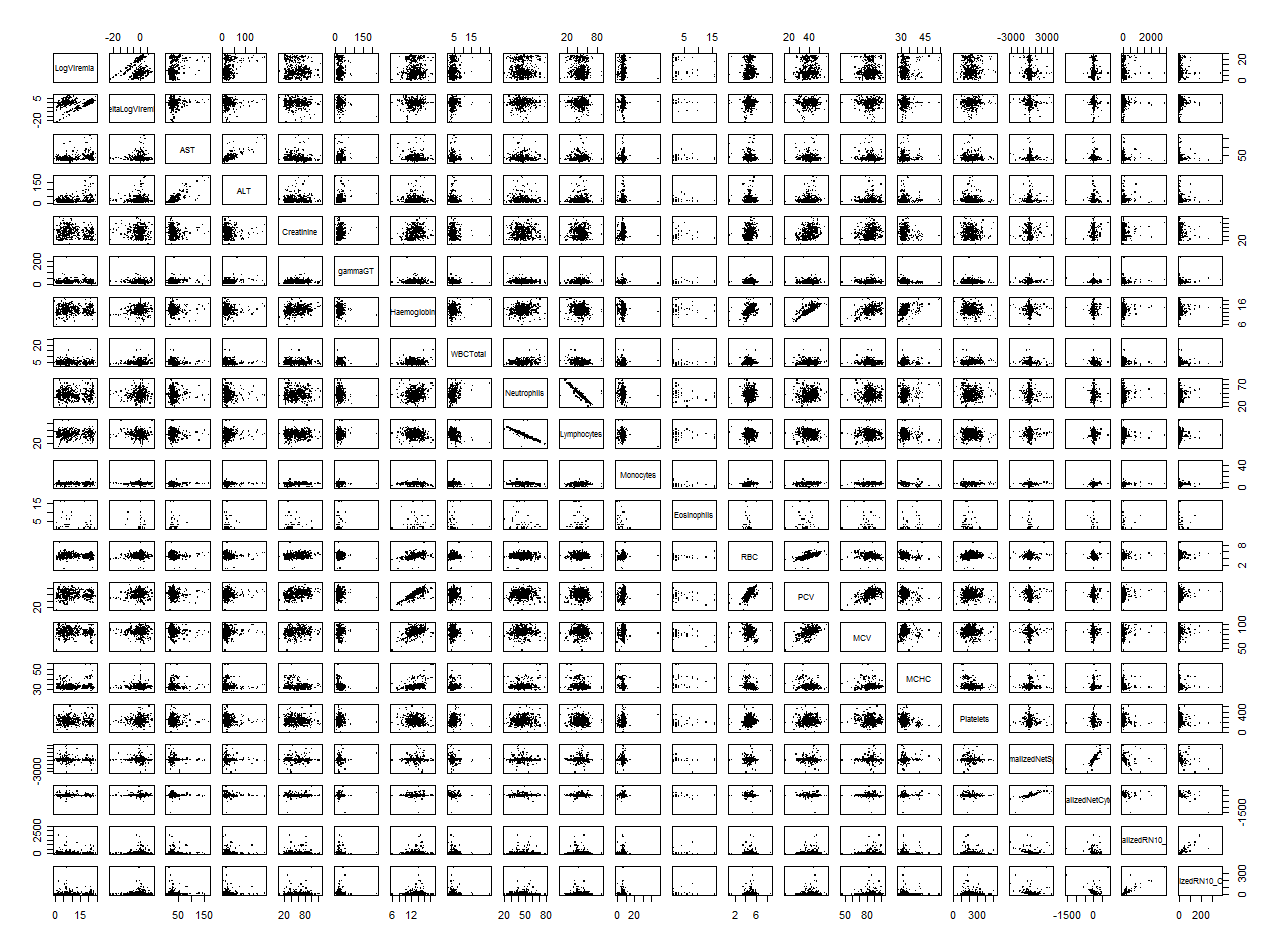


Exploratory Figure 1. Pairs plot for laboratory data. Here, the LogViremia is to base e. The R code to generate this figure (as well as to see more clearly the dimensions and names of the data) and the associated output are as follows:

**> labdata <- read.table("C:\\Users\\James Cavenaugh\\Documents\\labdata", header = TRUE)**

**> dim(labdata)**

[1] 572 25

**> names(labdata)**

[1] "Group" "Donor" "time" "copies.mL" "LogViremia"

[6] "DeltaLogViremia" "AST" "ALT" "Creatinine" "gammaGT"

[11] "Haemoglobin" "WBCTotal" "Neutrophils" "Lymphocytes" "Monocytes"

[16] "Eosinophils" "RBC" "PCV" "MCV" "MCHC"

[21] "Platelets" "NormalizedNetSpots" "NormalizedNetCytokine" "NormalizedRN10_Spots" "NormalizedRN10_Cytokine"

**> pairs(labdata[c(-1,-2,-3,-4)], pch = ".", cex = 2)**

**> round(cor(labdata[c(-1,-2,-3,-4)],use = "pairwise.complete.obs", method = "pearson"), 3)**

LogViremia DeltaLogViremia AST ALT Creatinine alphaGT Haemoglobin WBCTotal Neutrophils Lymphocytes Monocytes Eosinophils

LogViremia 1.000 0.176 0.326 0.361 -0.065 0.021 0.035 0.053 0.025 -0.034 -0.003 -0.138

DeltaLogViremia 0.176 1.000 0.082 0.028 -0.056 -0.160 0.131 0.086 0.036 -0.048 -0.016 -0.147

AST **0.326** 0.082 1.000 0.788 -0.013 0.200 -0.036 -0.018 -0.034 0.027 0.011 -0.134

ALT **0.361** 0.028 **0.788** 1.000 0.006 0.291 0.030 -0.045 -0.040 0.037 0.012 -0.057

Creatinine -0.065 -0.056 -0.013 0.006 1.000 0.151 0.236 -0.033 0.011 0.007 0.023 -0.098

alphaGT 0.021 -0.160 0.200 0.291 0.151 1.000 -0.055 -0.126 -0.075 0.080 -0.020 0.238

Haemoglobin 0.035 0.131 -0.036 0.030 0.236 -0.055 1.000 0.150 0.141 -0.129 -0.055 -0.041

WBCTotal 0.053 0.086 -0.018 -0.045 -0.033 -0.126 0.150 1.000 0.337 -0.305 -0.050 -0.060

Neutrophils 0.025 0.036 -0.034 -0.040 0.011 -0.075 0.141 **0.337** 1.000 -0.919 -0.108 -0.280

Lymphocytes -0.034 -0.048 0.027 0.037 0.007 0.080 -0.129 **-0.305** **-0.919** 1.000 -0.115 -0.051

Monocytes -0.003 -0.016 0.011 0.012 0.023 -0.020 -0.055 -0.050 -0.108 -0.115 1.000 -0.203

Eosinophils -0.138 -0.147 -0.134 -0.057 -0.098 0.238 -0.041 -0.060 -0.280 -0.051 -0.203 1.000

RBC 0.005 0.069 -0.085 0.007 0.201 -0.005 **0.495** 0.048 0.041 -0.050 0.119 0.203

PCV -0.003 0.076 -0.058 0.080 **0.347** 0.037 **0.798** 0.009 0.052 -0.045 0.105 -0.133

MCV 0.029 0.003 -0.054 0.109 0.203 0.065 **0.503** -0.097 -0.002 0.011 0.056 -0.207

MCHC 0.076 0.072 0.034 -0.052 -0.111 -0.178 **0.350** **0.307** 0.169 -0.169 -0.247 0.106

Platelets 0.010 0.073 0.002 -0.044 -0.056 0.000 -0.133 0.105 0.051 -0.068 0.141 -0.094

NormalizedNetSpots -0.117 -0.055 -0.044 -0.023 0.035 0.125 0.011 -0.063 -0.043 0.041 0.077 -0.192

NormalizedNetCytokine -0.094 -0.053 -0.004 -0.002 0.096 0.046 0.011 -0.010 -0.022 0.018 0.077 -0.184

NormalizedRN10_Spots -0.028 0.054 -0.008 0.026 0.111 0.046 0.120 -0.021 0.029 -0.038 -0.004 0.045

NormalizedRN10_Cytokine -0.043 0.047 -0.012 0.016 0.102 0.076 0.098 -0.010 0.030 -0.038 -0.027 0.032

RBC PCV MCV MCHC Platelets NormalizedNetSpots NormalizedNetCytokine NormalizedRN10_Spots NormalizedRN10_Cytokine

LogViremia 0.005 -0.003 0.029 0.076 0.010 -0.117 -0.094 -0.028 -0.043

DeltaLogViremia 0.069 0.076 0.003 0.072 0.073 -0.055 -0.053 0.054 0.047

AST -0.085 -0.058 -0.054 0.034 0.002 -0.044 -0.004 -0.008 -0.012

ALT 0.007 0.080 0.109 -0.052 -0.044 -0.023 -0.002 0.026 0.016

Creatinine 0.201 0.347 0.203 -0.111 -0.056 0.035 0.096 0.111 0.102

alphaGT -0.005 0.037 0.065 -0.178 0.000 0.125 0.046 0.046 0.076

Haemoglobin 0.495 0.798 0.503 0.350 -0.133 0.011 0.011 0.120 0.098

WBCTotal 0.048 0.009 -0.097 0.307 0.105 -0.063 -0.010 -0.021 -0.010

Neutrophils 0.041 0.052 -0.002 0.169 0.051 -0.043 -0.022 0.029 0.030

Lymphocytes -0.050 -0.045 0.011 -0.169 -0.068 0.041 0.018 -0.038 -0.038

Monocytes 0.119 0.105 0.056 -0.247 0.141 0.077 0.077 -0.004 -0.027

Eosinophils 0.203 -0.133 -0.207 0.106 -0.094 -0.192 -0.184 0.045 0.032

RBC 1.000 0.667 -0.098 -0.197 0.077 0.053 0.030 0.071 0.094

PCV **0.667** 1.000 0.550 -0.228 0.008 0.015 -0.009 0.129 0.116

MCV -0.098 **0.550** 1.000 -0.062 -0.095 0.043 0.015 0.095 0.045

MCHC -0.197 -0.228 -0.062 1.000 -0.227 0.042 0.059 0.008 0.000

Platelets 0.077 0.008 -0.095 -0.227 1.000 -0.041 -0.020 -0.059 -0.044

NormalizedNetSpots 0.053 0.015 0.043 0.042 -0.041 1.000 0.748 0.048 -0.023

NormalizedNetCytokine 0.030 -0.009 0.015 0.059 -0.020 **0.748** 1.000 0.166 -0.086

NormalizedRN10_Spots 0.071 0.129 0.095 0.008 -0.059 0.048 0.166 1.000 0.899

NormalizedRN10_Cytokine 0.094 0.116 0.045 0.000 -0.044 -0.023 -0.086 **0.899** 1.000

>

**Exploratory Table 1. Correlations for all laboratory data.**

**
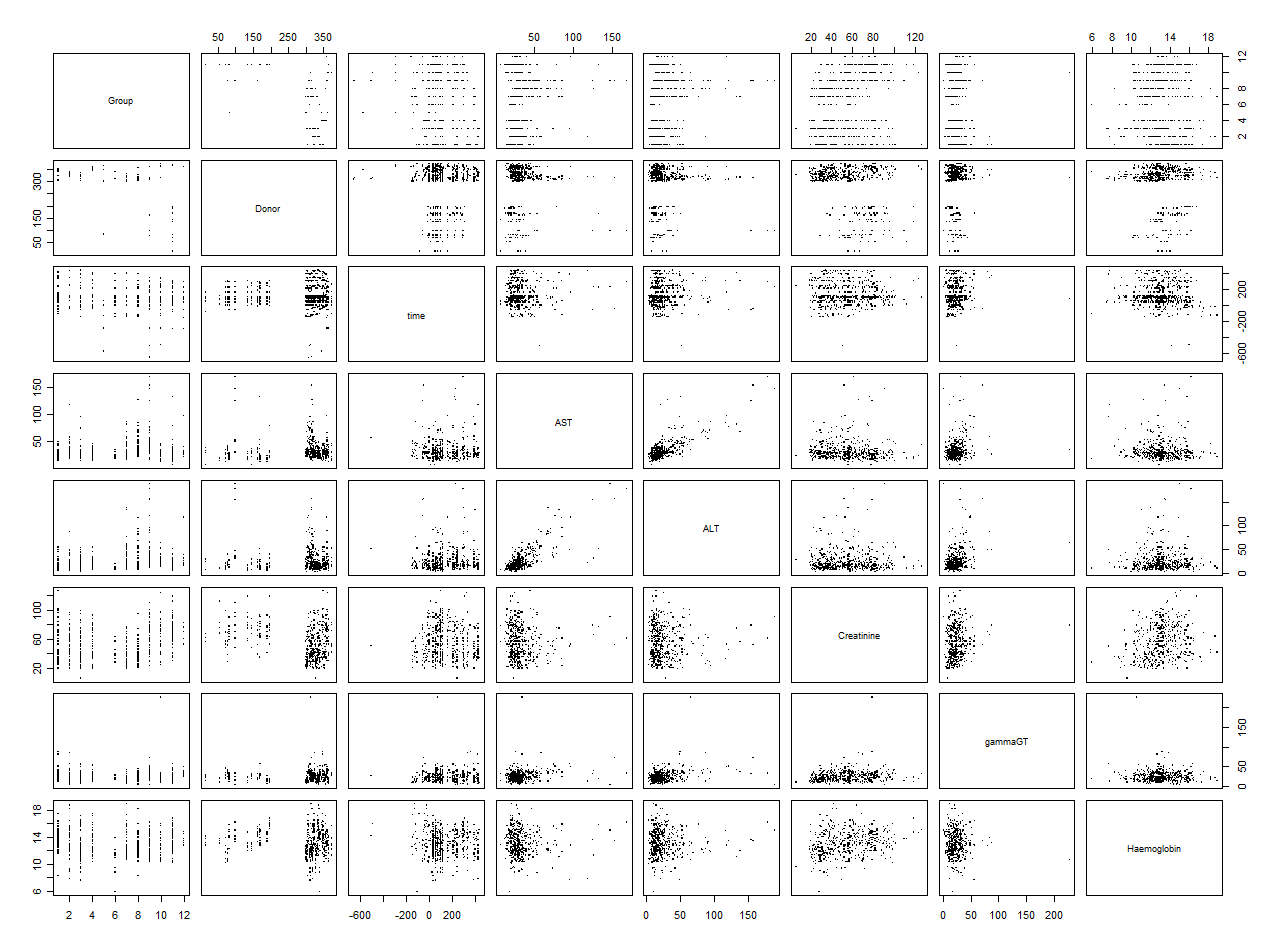
**

**Exploratory Figure 2. Pairs plot for group, donor, time, and biochemical data.** Some clustering of values for creatinine and hemoglobin is seen but occurs without regard to allocation group. The dataset is provided as a supplementary file. The R code and output used to prepare this plot is as follows:

> labdata <- read.table("C:\\Users\\James Cavenaugh\\Documents\\labdata", header = TRUE)

> names(labdata)

[1] "Group" "Donor"

[3] "time" "copies.mL"

[5] "LogViremia" "DeltaLogViremia"

[7] "AST" "ALT"

[9] "Creatinine" "gammaGT"

[11] "Haemoglobin" "WBCTotal"

[13] "Neutrophils" "Lymphocytes"

[15] "Monocytes" "Eosinophils"

[17] "RBC" "PCV"

[19] "MCV" "MCHC"

[21] "Platelets" "NormalizedNetSpots"

[23] "NormalizedNetCytokine" "NormalizedRN10_Spots"

[25] "NormalizedRN10_Cytokine"

> pairs(labdata[c(1,2,3,7,8,9,10,11)], pch = ".", cex = 2)


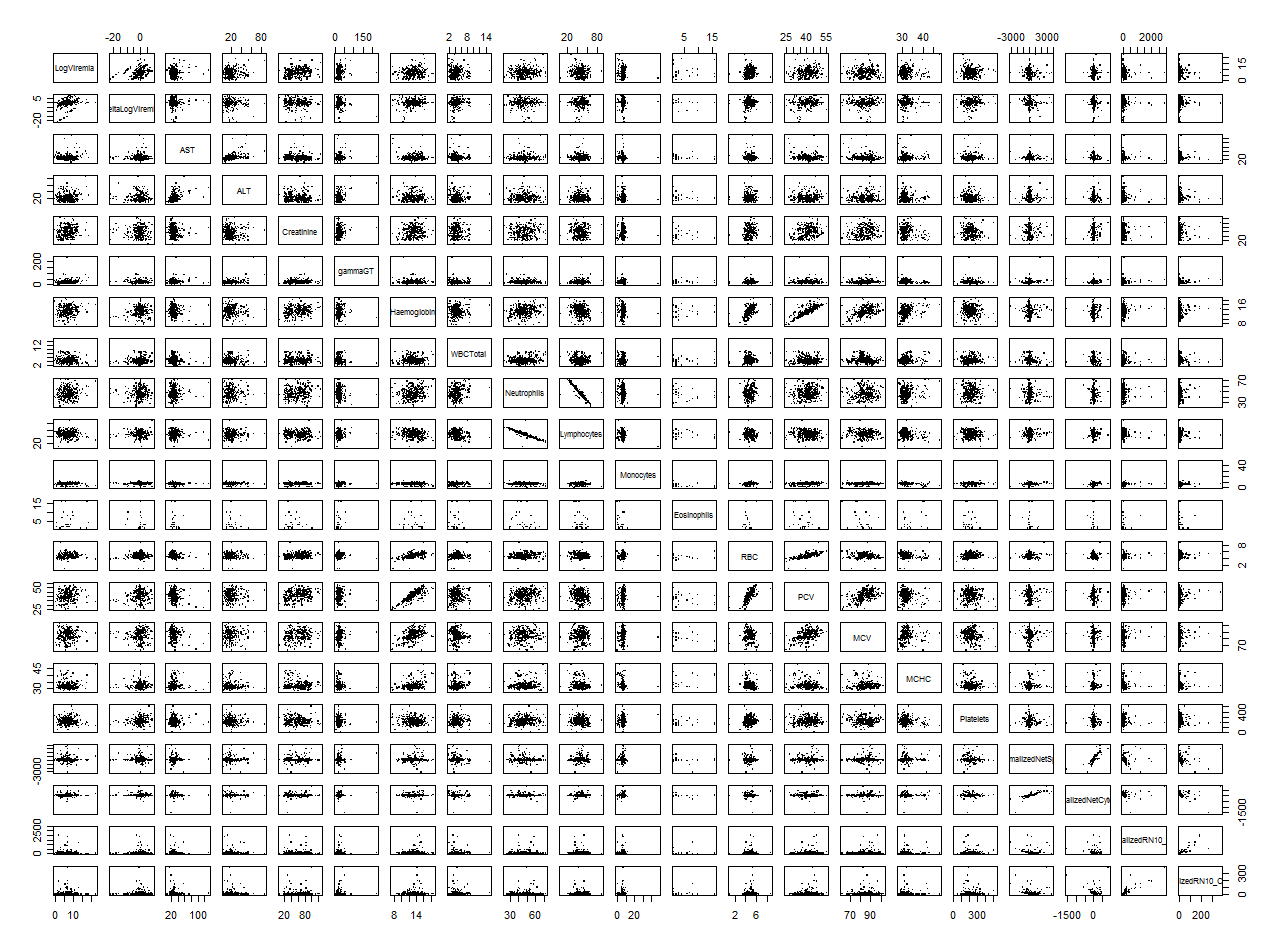


**Exploratory Figure 3. Pairs plot for HBeAg negative subset.** This is as in Exploratory Figure 1 except for only groups A, B, C, D, I-originally, and J. The corresponding correlation matrix is as follows:

LogViremia DeltaLogViremia AST ALT Creatinine gammaGT Haemoglobin WBCTotal Neutrophils Lymphocytes Monocytes Eosinophils

LogViremia 1.000 0.272 0.053 0.097 0.234 0.148 0.108 0.010 0.094 -0.067 -0.113 0.115

DeltaLogViremia 0.272 1.000 0.028 -0.056 -0.035 -0.203 0.175 0.127 -0.018 0.058 -0.024 -0.118

AST 0.053 0.028 1.000 0.494 -0.043 0.120 -0.144 -0.040 0.023 -0.023 -0.013 -0.140

ALT 0.097 -0.056 **0.494** 1.000 -0.045 0.391 -0.025 -0.038 0.041 -0.038 -0.029 -0.169

Creatinine 0.234 -0.035 -0.043 -0.045 1.000 0.126 0.187 -0.011 0.089 -0.038 -0.006 -0.243

gammaGT 0.148 -0.203 0.120 **0.391** 0.126 1.000 -0.041 -0.147 -0.062 0.079 -0.053 0.190

Haemoglobin 0.108 0.175 -0.144 -0.025 0.187 -0.041 1.000 0.082 0.229 -0.205 -0.071 -0.006

WBCTotal 0.010 0.127 -0.040 -0.038 -0.011 -0.147 0.082 1.000 0.277 -0.235 -0.020 0.157

Neutrophils 0.094 -0.018 0.023 0.041 0.089 -0.062 0.229 0.277 1.000 -0.920 -0.063 -0.205

Lymphocytes -0.067 0.058 -0.023 -0.038 -0.038 0.079 -0.205 -0.235 **-0.920** 1.000 -0.197 -0.252

Monocytes -0.113 -0.024 -0.013 -0.029 -0.006 -0.053 -0.071 -0.020 -0.063 -0.197 1.000 -0.287

Eosinophils 0.115 -0.118 -0.140 -0.169 -0.243 0.190 -0.006 0.157 -0.205 -0.252 -0.287 1.000

RBC 0.071 0.098 -0.201 -0.033 0.185 -0.035 **0.571** 0.098 0.119 -0.124 0.083 0.226

PCV 0.104 0.124 -0.161 0.035 **0.311** 0.020 **0.830** 0.030 0.148 -0.137 0.075 -0.265

MCV 0.051 0.032 -0.132 0.080 0.182 0.098 **0.500** -0.140 0.032 -0.021 0.040 **-0.426**

MCHC 0.021 0.070 0.017 -0.062 -0.124 -0.159 **0.320** 0.189 0.161 -0.154 -0.222 **0.498**

Platelets -0.066 0.166 -0.019 -0.092 -0.066 -0.056 -0.121 0.057 0.059 -0.075 0.145 -0.184

NormalizedNetSpots -0.012 -0.061 -0.036 0.029 0.045 0.164 0.014 -0.132 -0.077 0.071 0.132 -0.198

NormalizedNetCytokine -0.008 -0.068 -0.010 -0.002 0.137 0.066 0.002 -0.040 -0.056 0.050 0.123 -0.214

NormalizedRN10_Spots -0.039 0.033 -0.039 -0.013 0.087 0.060 0.110 -0.008 0.011 -0.019 0.005 0.019

NormalizedRN10_Cytokine -0.031 0.033 -0.044 -0.024 0.057 0.080 0.086 0.016 0.028 -0.033 -0.016 0.004

RBC PCV MCV MCHC Platelets NormalizedNetSpots NormalizedNetCytokine NormalizedRN10_Spots NormalizedRN10_Cytokine

LogViremia 0.071 0.104 0.051 0.021 -0.066 -0.012 -0.008 -0.039 -0.031

DeltaLogViremia 0.098 0.124 0.032 0.070 0.166 -0.061 -0.068 0.033 0.033

AST -0.201 -0.161 -0.132 0.017 -0.019 -0.036 -0.010 -0.039 -0.044

ALT -0.033 0.035 0.080 -0.062 -0.092 0.029 -0.002 -0.013 -0.024

Creatinine 0.185 0.311 0.182 -0.124 -0.066 0.045 0.137 0.087 0.057

gammaGT -0.035 0.020 0.098 -0.159 -0.056 0.164 0.066 0.060 0.080

Haemoglobin 0.571 0.830 0.500 0.320 -0.121 0.014 0.002 0.110 0.086

WBCTotal 0.098 0.030 -0.140 0.189 0.057 -0.132 -0.040 -0.008 0.016

Neutrophils 0.119 0.148 0.032 0.161 0.059 -0.077 -0.056 0.011 0.028

Lymphocytes -0.124 -0.137 -0.021 -0.154 -0.075 0.071 0.050 -0.019 -0.033

Monocytes 0.083 0.075 0.040 -0.222 0.145 0.132 0.123 0.005 -0.016

Eosinophils 0.226 -0.265 -0.426 0.498 -0.184 -0.198 -0.214 0.019 0.004

RBC 1.000 0.699 -0.084 -0.113 0.056 0.104 0.048 0.066 0.094

PCV **0.699** 1.000 0.511 -0.170 -0.038 0.044 -0.007 0.110 0.103

MCV -0.084 **0.511** 1.000 -0.012 -0.141 0.056 0.018 0.072 0.020

MCHC -0.113 -0.170 -0.012 1.000 -0.139 0.019 0.050 0.034 0.019

Platelets 0.056 -0.038 -0.141 -0.139 1.000 -0.043 -0.020 -0.038 -0.019

NormalizedNetSpots 0.104 0.044 0.056 0.019 -0.043 1.000 0.760 0.131 0.022

NormalizedNetCytokine 0.048 -0.007 0.018 0.050 -0.020 **0.760** 1.000 0.195 -0.086

NormalizedRN10_Spots 0.066 0.110 0.072 0.034 -0.038 0.131 0.195 1.000 0.899

NormalizedRN10_Cytokine 0.094 0.103 0.020 0.019 -0.019 0.022 -0.086 **0.899** 1.000


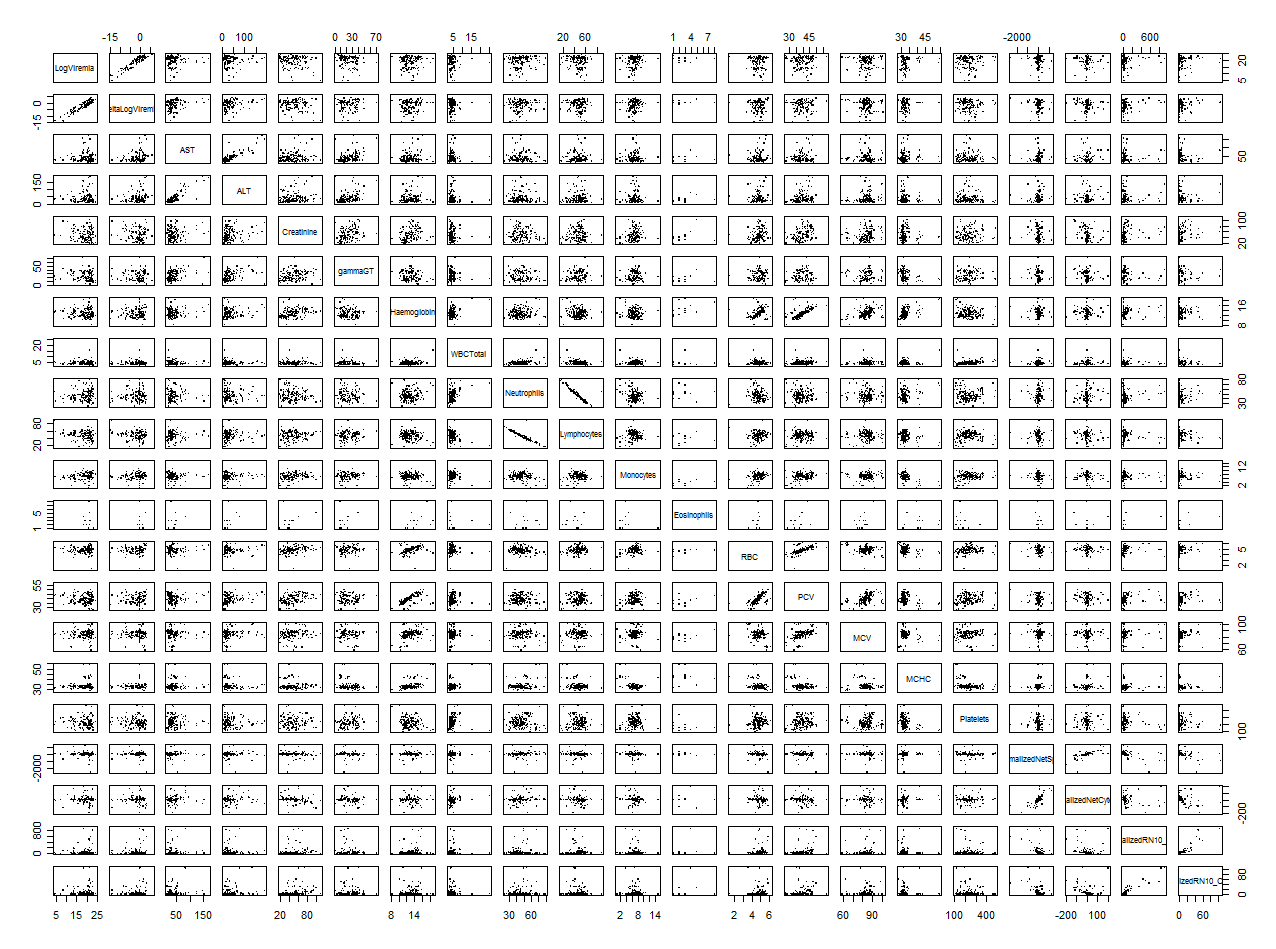


**Exploratory Figure 4. Pairs plot for HBeAg positive subset.** This is as in Exploratory Figure 1 except for only groups F, G, I, and J-originally. The corresponding correlation matrix is as follows:

LogViremia DeltaLogViremia AST ALT Creatinine gammaGT Haemoglobin WBCTotal Neutrophils Lymphocytes Monocytes Eosinophils

LogViremia 1.000 0.928 0.053 0.017 -0.205 -0.146 0.016 0.022 0.129 -0.267 0.103 -0.089

DeltaLogViremia **0.928** 1.000 0.228 0.163 -0.154 -0.026 0.060 0.032 0.104 -0.223 0.056 0.106

AST 0.053 0.228 1.000 0.870 0.115 0.413 0.084 -0.037 -0.048 0.026 0.017 0.063

ALT 0.017 0.163 **0.870** 1.000 0.153 0.394 0.099 -0.089 -0.055 0.039 0.034 0.422

Creatinine -0.205 -0.154 0.115 0.153 1.000 0.246 0.299 -0.035 -0.131 0.089 0.171 0.305

gammaGT -0.146 -0.026 **0.413** **0.394**  0.246 1.000 -0.139 -0.118 -0.122 0.090 0.152 0.193

Haemoglobin 0.016 0.060 0.084 0.099 0.299 -0.139 1.000 0.362 0.081 -0.086 -0.082 0.422

WBCTotal 0.022 0.032 -0.037 -0.089 -0.035 -0.118 **0.362** 1.000 0.384 -0.365 -0.143 -0.156

Neutrophils 0.129 0.104 -0.048 -0.055 -0.131 -0.122 0.081 **0.384** 1.000 -0.912 -0.247 -0.093

Lymphocytes -0.267 -0.223 0.026 0.039 0.089 0.090 -0.086 **-0.365** **-0.912** 1.000 0.097 -0.014

Monocytes 0.103 0.056 0.017 0.034 0.171 0.152 -0.082 -0.143 -0.247 0.097 1.000 -0.200

Eosinophils -0.089 0.106 0.063 **0.422** **0.305** 0.193 **0.422** -0.156 -0.093 -0.014 -0.200 1.000

RBC -0.086 -0.003 0.021 0.035 0.233 0.102 0.275 -0.029 -0.136 0.119 0.261 **0.341**

PCV -0.056 -0.025 0.080 0.209 **0.389** 0.090 **0.654** 0.018 -0.104 0.095 0.210 **0.473**

MCV 0.044 -0.029 0.015 0.193 0.163 -0.036 **0.357** -0.012 -0.001 -0.013 0.051 **0.611**

MCHC 0.084 0.108 0.007 -0.108 -0.088 -0.267 **0.495** **0.476** 0.295 -0.276 **-0.416** -0.041

Platelets 0.011 -0.096 -0.035 -0.089 0.037 0.160 -0.138 0.125 -0.012 -0.015 0.144 -0.245

NormalizedNetSpots -0.107 -0.095 0.036 0.018 -0.087 -0.068 -0.047 0.111 0.059 -0.037 -0.083 -0.255

NormalizedNetCytokine -0.069 -0.042 0.114 0.098 -0.229 -0.129 0.053 0.124 0.110 -0.099 -0.080 -0.013

NormalizedRN10_Spots 0.116 0.140 0.069 0.128 0.181 -0.030 0.130 -0.044 0.113 -0.129 -0.014 0.162

NormalizedRN10_Cytokine 0.062 0.102 0.122 0.180 0.292 0.053 0.115 -0.067 0.068 -0.096 -0.046 0.176

RBC PCV MCV MCHC Platelets NormalizedNetSpots NormalizedNetCytokine NormalizedRN10_Spots NormalizedRN10_Cytokine

LogViremia -0.086 -0.056 0.044 0.084 0.011 -0.107 -0.069 0.116 0.062

DeltaLogViremia -0.003 -0.025 -0.029 0.108 -0.096 -0.095 -0.042 0.140 0.102

AST 0.021 0.080 0.015 0.007 -0.035 0.036 0.114 0.069 0.122

ALT 0.035 0.209 0.193 -0.108 -0.089 0.018 0.098 0.128 0.180

Creatinine 0.233 0.389 0.163 -0.088 0.037 -0.087 -0.229 0.181 0.292

gammaGT 0.102 0.090 -0.036 -0.267 0.160 -0.068 -0.129 -0.030 0.053

Haemoglobin 0.275 0.654 0.357 0.495 -0.138 -0.047 0.053 0.130 0.115

WBCTotal -0.029 0.018 -0.012 0.476 0.125 0.111 0.124 -0.044 -0.067

Neutrophils -0.136 -0.104 -0.001 0.295 -0.012 0.059 0.110 0.113 0.068

Lymphocytes 0.119 0.095 -0.013 -0.276 -0.015 -0.037 -0.099 -0.129 -0.096

Monocytes 0.261 0.210 0.051 -0.416 0.144 -0.083 -0.080 -0.014 -0.046

Eosinophils 0.341 0.473 0.611 -0.041 -0.245 -0.255 -0.013 0.162 0.176

RBC 1.000 0.599 -0.277 -0.358 0.127 -0.180 -0.091 0.078 0.084

PCV **0.599** 1.000 0.480 -0.331 0.141 -0.161 -0.072 0.171 0.140

MCV -0.277 **0.480** 1.000 -0.097 -0.005 -0.022 -0.008 0.142 0.095

MCHC **-0.358** **-0.331** -0.097 1.000 -0.342 0.133 0.167 -0.047 -0.044

Platelets 0.127 0.141 -0.005 **-0.342** 1.000 0.006 0.010 -0.110 -0.119

NormalizedNetSpots -0.180 -0.161 -0.022 0.133 0.006 1.000 0.694 -0.512 -0.456

NormalizedNetCytokine -0.091 -0.072 -0.008 0.167 0.010 **0.694** 1.000 -0.126 -0.140

NormalizedRN10_Spots 0.078 0.171 0.142 -0.047 -0.110 **-0.512** -0.126 1.000 0.923

NormalizedRN10_Cytokine 0.084 0.140 0.095 -0.044 -0.119 **-0.456** -0.140 **0.923** 1.000

>
