# Supplementary material for: Partially Randomized, Non-Blinded Trial of DNA and MVA Therapeutic Vaccines Based on Hepatitis B Virus Surface Protein for Chronic HBV Infection
Source: PLoS One. 2011 Feb 15;6(2):e14626. doi: 10.1371/journal.pone.0014626 (PMC3039644; doi:10.1371/journal.pone.0014626)
Supplement: File S3 — Supplementary Material: Results of Individual Kinetics. (0.19 MB DOC) [file pone.0014626.s003.doc]

Kinetics Figure 1. Time courses of viremia for individual volunteers in various treatment groups.

Kinetics Figure 2. ELISpot counts in medium only. Note that the scale for Group A is twice that for the other groups.

Kinetics Figure 3. Net ELISpot counts (subtracting medium). Note that the scale for Group A is twice that for the other groups.

**
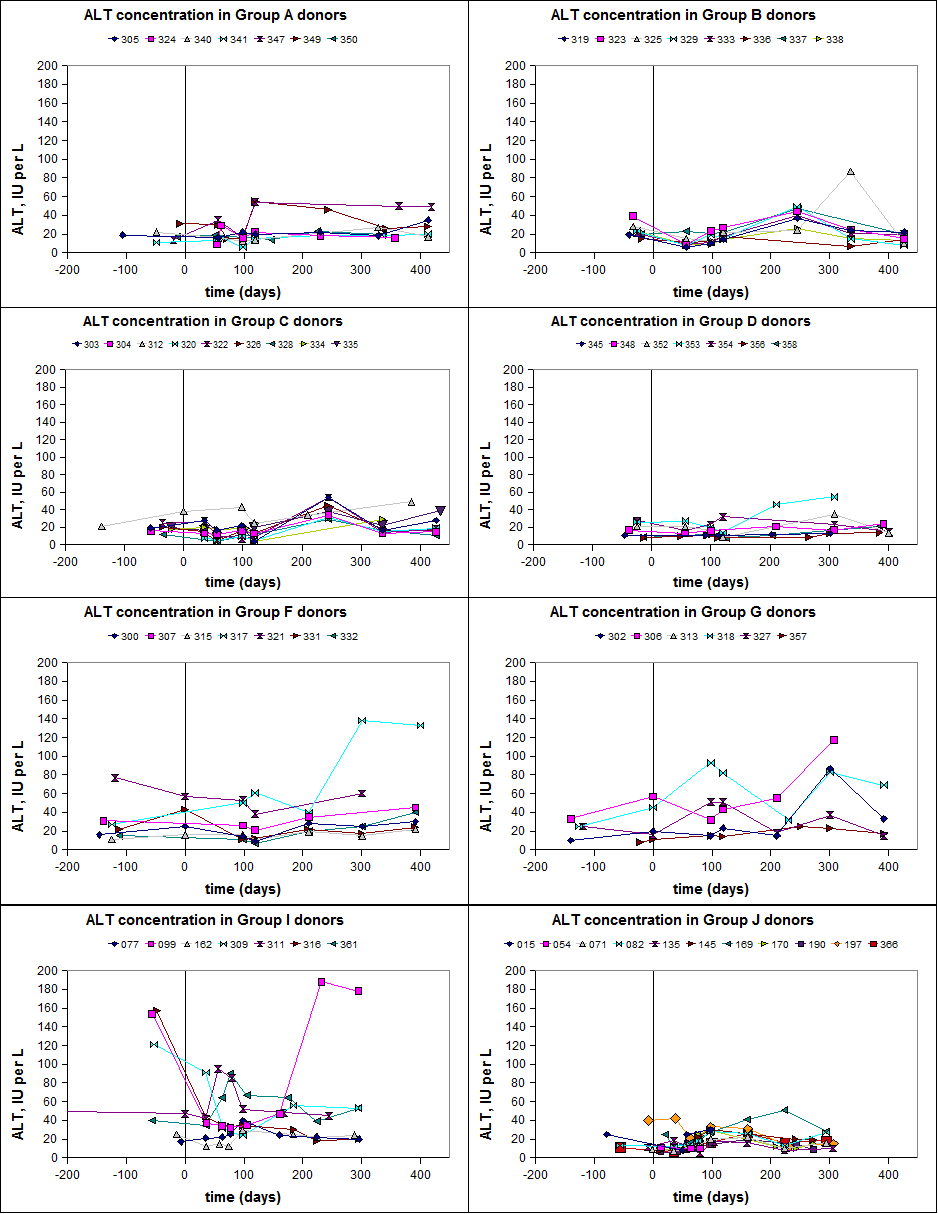
**

Kinetics Figure 4. Time courses of ALT for individual volunteers in various treatment groups.

Kinetics Figure 5. Phenotype of IFN-γ producing cells by ICCS in Group J. Left panels are for medium only; right panels are with peptide stimulation. From top to bottom, these panels show IFN-γ production by total lymphocytes, T cells (CD3^+^), NK cells (CD16^+^), and NKT cells (CD3^+^CD16^+^). These graphs were generated automatically from a form in our database [58], the input for which is indicated in each panel’s header.

Kinetics Figure 6. Phenotype of IFN-γ producing cells by ICCS in Group I. Left panels are for medium only; right panels are with peptide stimulation. From top to bottom, these panels show IFN-γ production by total lymphocytes, T cells (CD3^+^), NK cells (CD16^+^), and NKT cells (CD3^+^CD16^+^). As in Kinetics Figure 5, some donors have different times shown because each FCS dot plot region was considered acceptable or not (a yes/no field), and only those which stained acceptably were included in each datum.
