# Supplementary material for: Partially Randomized, Non-Blinded Trial of DNA and MVA Therapeutic Vaccines Based on Hepatitis B Virus Surface Protein for Chronic HBV Infection
Source: PLoS One. 2011 Feb 15;6(2):e14626. doi: 10.1371/journal.pone.0014626 (PMC3039644; doi:10.1371/journal.pone.0014626)
Supplement: File S4 — Supplementary Material: Results of Unsolicited Adverse Events. (0.09 MB DOC) [file pone.0014626.s004.doc]

**Table of unsolicited adverse events**

|  | groups with low dose MVA.HBs  *n* = 23^♠^ | groups with high dose MVA.HBs  *n* = 23^♥^ | control groups  *n* = 23^♣^ |
| --- | --- | --- | --- |
| **Body as a whole** |  |  |  |
| Anemia | 20 | 0 | 17 |
| Body ache | 4 | 1 | 1 |
| Chicken pox | 1 | 0 | 0 |
| Dizziness | 6 | 0 | 1 |
| Fatigue | 1 | 0 | 2 |
| Fever | 8 | 3 | 14 |
| Headache | 28 | 2 | 20 |
| Malaria | 16 | 1 | 16 |
|  |  |  |  |
| **Dental** |  |  |  |
| Abscess | 0 | 0 | 1 |
| Gum ache | 1 | 0 | 0 |
| Swollen jaw | 0 | 0 | 1 |
| Tongue ulcer or pain | 2 | 0 | 1 |
| Tooth ache, caries | 5 | 1 | 2 |
| Tooth, broken | 0 | 0 | 1 |
|  |  |  |  |
| **Dermatological** |  |  |  |
| Boils | 5 | 1 | 5 |
| Eczema | 1 | 1 | 1 |
| Fungal skin rash | 0 | 1 | 2 |
| Foot sores (septic, ulcers) | 1 | 0 | 0 |
| Onychomycosis | 1 | 0 | 0 |
| Paronychia | 1 | 0 | 0 |
| Penile sores | 1 | 0 | 0 |
| Pitiriasis versicolor | 0 | 1 | 1 |
| Pimples, scalp sepsis | 1 | 0 | 1 |
| Pruritis | 4 | 0 | 3 |
| Pustules | 2 | 0 | 0 |
| Rash | 5 | 1 | 4 |
| Ring worm | 5 | 0 | 5 |
| Scabies | 5 | 3 | 1 |
| Shingles | 0 | 0 | 1 |
| Tinea versicolor | 4 | 0 | 2 |
| Tinea cruris | 0 | 0 | 1 |
| Tinea capitis | 0 | 0 | 1 |
| Tinea pedis | 1 | 0 | 0 |
|  |  |  |  |
| **Gastrointestinal** |  |  |  |
| Abdominal pain | 10 | 4 | 13 |
| Diarrhea | 5 | 0 | 1 |
| Food poisoning | 0 | 0 | 1 |
| Worms | 0 | 1 | 0 |
|  |  |  |  |
| **Respiratory** |  |  |  |
| Bronchitis | 1 | 0 | 0 |
| Chest pain | 9 | 0 | 6 |
| Cold, runny nose, coryza | 7 | 0 | 2 |
| Cough | 14 | 0 | 6 |
| Nasal congestion | 1 | 0 | 0 |
| URTI, sore throat | 7 | 2 | 5 |
|  |  |  |  |
| **Miscellaneous** |  |  |  |
| Back ache | 0 | 0 | 4 |
| Bone pain | 0 | 0 | 1 |
| Blistered or ulcers on lip | 2 | 0 | 1 |
| Bruises | 7 | 0 | 4 |
| Ear complaints | 3 | 0 | 4 |
| Eye complaints | 4 | 0 | 1 |
| Foot pain | 0 | 0 | 1 |
| Injuries (cuts, wounds) | 7 | 1 | 6 |
| “heat on the back” | 0 | 0 | 1 |
| Hemorrhoids | 0 | 0 | 2 |
| Joint pain (not bruises) | 0 | 0 | 2 |
| Low appetite | 0 | 0 | 1 |
| Side pain | 1 | 0 | 0 |
| Swollen right leg | 1 | 0 | 0 |
| Tender lymphadenopathy | 0 | 1 | 1 |
| Ulcers | 13 | 1 | 0 |
| Urethritis | 2 | 1 | 0 |

**^♠^** Groups A, C, G, and #310 of group exclude (originally of group G).

^♥^ Groups I, J, I-originally, and J-originally.

^♣^ Groups B, D, F, and #339 of group exclude (originally of group F).
