# Supplementary material for: Partially Randomized, Non-Blinded Trial of DNA and MVA Therapeutic Vaccines Based on Hepatitis B Virus Surface Protein for Chronic HBV Infection
Source: PLoS One. 2011 Feb 15;6(2):e14626. doi: 10.1371/journal.pone.0014626 (PMC3039644; doi:10.1371/journal.pone.0014626)
Supplement: File S5 — Supplementary Material: Results of Epitope Screening. (0.14 MB DOC) [file pone.0014626.s005.doc]

**SUPPLEMENTARY MATERIAL: RESULTS of EPITOPE SCREENING**

## ***Epitopes involved***

To determine the peptide epitopes that may be involved in the net immune responses in cases of low background, we assumed a Poisson distribution between spots in the medium-only wells compared with the spots in the peptide-containing wells and a null hypothesis that none of the peptides were epitopes. A peptide was considered as a potential epitope if both of the pools containing that peptide showed statistically significantly more spots than the average of the background spots at a specified level of uncertainty. The type I error tolerance was set at α = 0.05. Only samples having < 10 spots in the medium-only wells were included. This is a sound way of screening for potential epitopes, though it may not be conclusive in each case, especially if there are many responses. Some donors did show potential epitopes for the HBV medium surface protein as shown below in Supplementary Figure 1. The most frequent peptide to appear as a potential epitope is peptide 44.

Although often only spot forming units (SFU) are reported for ELISpot assays, this technique offers additional, complementary information on the relative amounts of IFN-γ produced in different wells, besides the number of cells producing it. Because production of secreted proteins is not all or nothing, a measure of the amount produced could be as meaningful as the number of producers. This is especially true when using sensitive count settings that can count small spots, as done for the majority of plates in this trial. Supplementary Figures 2 and 3 show the relative amounts of IFN-γ produced for the medium-only and for net response, respectively. The quantity actually measured is the sum of the product of the spot intensity (proportional to the amount of cytokine secreted) times the number of spots and is therefore on a relative, pixel-based scale. Both measures (i.e., How many cells make IFN-γ? and relatively how much IFN-γ is made?) are subject to subjective biases insofar as what one chooses as the best count settings and the manual editing necessary to remove spot artifacts. The precision of both measures is about the same since spot detection and spot quantitation occur on the same spots with the same software, and since other factors usually dominate. The intra-assay, inter-assay, and biological variability of ELISpot assays have been examined for SFU output and are often very high (>50% CV) [4]. Here, the general features are the same in both sets of plots, except that Group C appears to have more clearly a vaccine-induced rise in net response in the relative IFN-γ measure than by spot counts.

**REFERENCES**

1. Addo MM, Yu XG, Rathod A, Cohen D, Eldridge RL, et al. (2003) Comprehensive Epitope Analysis of Human Immunodeficiency Virus Type 1 (HIV-1)-Specific T-Cell Responses Directed against the Entire Expressed HIV-1 Genome Demonstrate Broadly Directed Responses, but No Correlation to Viral Load. J Virol 77: 2081-2092.

2. Gillespie Geraldine MA, Pinheiro S, Sayeid-Al-Jamee M, Alabi A, Kaye S, et al. (2005) CD8^+^ T cell responses to human immunodeficiency viruses type 2 (HIV-2) and type 1 (HIV-1) gag proteins are distinguishable by magnitude and breadth but not cellular phenotype. European Journal of Immunology 35: 1445-1453.

3. Keating SM, Bollinger RC, Quinn TC, Jackson JB, Carruth LM (2002) Cross-Clade T Lymphocyte-Mediated Immunity to HIV Type 1: Implications for Vaccine Design and Immunodetection Assays. AIDS Research and Human Retroviruses 18: 1067-1079.

4. Lathey JL (2003) Preliminary Steps Toward Validating a Clinical Bioassay: A Case Study of the ELIspot Assay. BIOPHARM INTERNATIONAL 16: 42-51.

Supplementary Figure 1. Frequency of ELISpot responses to each HBV peptide. These were predicted by mapping significant responses in 2 wells onto the 2-D array formed by the composition of the peptide pools. To improve specificity assays with background levels greater than 10 spots per million PBMC were excluded. Results are shown in blue for subjects with the very common HLA-A2 haplotype and in red for all subjects. Type I error tolerance α = 0.05. (HLA haplotyping was also outsourced to another MRC, Fajara laboratory which performs this routinely.)

***Additional ELISpot output***

Supplementary Figure 2. IFN-γ produced in medium only. The scale is relative, based on the spot intensity (measured in pixels) times the number of spots.

Supplementary Figure 3. Net IFN-γ production. The scale is relative. Note the different ordinate axis scale for Group A compared to the others.
