# Supplementary material for: Partially Randomized, Non-Blinded Trial of DNA and MVA Therapeutic Vaccines Based on Hepatitis B Virus Surface Protein for Chronic HBV Infection
Source: PLoS One. 2011 Feb 15;6(2):e14626. doi: 10.1371/journal.pone.0014626 (PMC3039644; doi:10.1371/journal.pone.0014626)
Supplement: File S6 — Supplementary Material: Results of Statistical Significance of ELISpot. (0.05 MB DOC) [file pone.0014626.s006.doc]

| **Group** | ***n*** | **Treatment vs Pre-treatment** | **Post-treatment vs Pre-treatment** | Follow-up vs Pre-treatment |
| --- | --- | --- | --- | --- |
| A | 7 | 5.49 × 10^-1^ | 1.83 × 10^-1^ | 2.28 × 10^-1^ |
| B | 8 | 3.27 × 10^-1^ | 1.57 × 10^-1^ | 8.29 × 10^-1^ |
| C | 9 | 2.45 × 10^-1^ | 1.17 × 10^-1^ | 4.87 × 10^-1^ |
| D | 7 |  | 1.63 × 10^-1^ | 2.81 × 10^-1^ |
| F | 7 | 2.22 × 10^-1^ | 5.72 × 10^-1^ | 2.93 × 10^-1^ |
| G | 6 | 1.03 × 10^-1^ | 4.05 × 10^-1^ | 4.93 × 10^-1^ |
| I | 7 | 5.44 × 10^-1^ | 2.97 × 10^-1^ | 7.55 × 10^-1^ |
| J | 11 | 8.55 × 10^-1^ | 1.54 × 10^-1^ | 1.49 × 10^-1^ |

**Supplementary Table 1. Paired 2-tailed *t* test *p* values within each group for ELISpot data (normalized net IFN-γ cytokine).**

| **Group** | ***n*** | **Treatment vs Pre-treatment** | **Post-treatment vs Pre-treatment** | Follow-up vs Pre-treatment |
| --- | --- | --- | --- | --- |
| A | 7 | 8.88 × 10^-2^ | 9.79 × 10^-2^ | 6.86 × 10^-3^ |
| B | 8 | 8.84 × 10^-1^ | 7.21 × 10^-1^ | 5.95 × 10^-1^ |
| C | 9 | 7.96 × 10^-1^ | 3.63 × 10^-2^ | 1.33 × 10^-1^ |
| D | 7 |  | 7.57 × 10^-1^ | 3.76 × 10^-1^ |
| F | 7 | 3.85 × 10^-1^ | 4.46 × 10^-1^ | 2.31 × 10^-2^ |
| G | 6 | 3.05 × 10^-1^ | 3.18 × 10^-1^ | 2.12 × 10^-1^ |
| I | 7 | 2.01 × 10^-1^ | 7.16 × 10^-2^ | 3.55 × 10^-1^ |
| J | 11 | 3.18 × 10^-1^ | 5.56 × 10^-2^ | 1.58 × 10^-1^ |

**Supplementary Table 2. Paired 2-tailed *t* test *p* values within each group for ELISpot data (normalized background spots).**

| **Group** | ***n*** | **Treatment vs Pre-treatment** | **Post-treatment vs Pre-treatment** | Follow-up vs Pre-treatment |
| --- | --- | --- | --- | --- |
| A | 7 | 1.38 × 10^-1^ | 9.60 × 10^-2^ | 1.31 × 10^-3^ |
| B | 8 | 8.10 × 10^-1^ | 6.83 × 10^-1^ | 4.94 × 10^-1^ |
| C | 9 | 9.77 × 10^-1^ | 5.75 × 10^-2^ | 3.84 × 10^-1^ |
| D | 7 |  | 1.18 × 10^-1^ | 8.67 × 10^-2^ |
| F | 7 | 4.89 × 10^-1^ | 3.57 × 10^-1^ | 4.19 × 10^-2^ |
| G | 6 | 3.36 × 10^-1^ | 6.72 × 10^-1^ | 3.70 × 10^-1^ |
| I | 7 | 1.88 × 10^-1^ | 5.16 × 10^-2^ | 1.99 × 10^-1^ |
| J | 11 | 4.00 × 10^-1^ | 2.12 × 10^-2^ | 4.68 × 10^-2^ |

**Supplementary Table 3. Paired 2-tailed *t* test *p* values within each group for ELISpot data (normalized background cytokine).**
