# Supplementary material for: Partially Randomized, Non-Blinded Trial of DNA and MVA Therapeutic Vaccines Based on Hepatitis B Virus Surface Protein for Chronic HBV Infection
Source: PLoS One. 2011 Feb 15;6(2):e14626. doi: 10.1371/journal.pone.0014626 (PMC3039644; doi:10.1371/journal.pone.0014626)
Supplement: Protocol S2 — Trial Protocol. (0.41 MB DOC) [file pone.0014626.s009.doc]

University of Oxford

**Clinical Trial Protocol**

**A phase II clinical trial to evaluate two administrations of plasmid DNA (pSG2.HBs) and two administrations of modified vaccinia virus Ankara (MVA.HBs) expressing hepatitis B surface antigen combined with lamivudine in Gambian subjects with eAg-positive chronic hepatitis B virus infection: HBs004**

**Protocol No:** HBs004

Original protocol: 19^th^ April 2002

This document is the property of the University of Oxford. It may not be used, divulged or published without the consent of University of Oxford.

**Principal Investigator:** Samuel J McConkey

University of Oxford

**Funding Agency:** Wellcome Trust

The Wellcome Building 183 Euston Road

London NW1 2BE, United Kingdom

**Scientific co-investigators**

Adrian VS Hill

University of Oxford

Assan Jaye

Medical Research Council Laboratories, Fajara, The Gambia

Maimuna Mendy

Medical Research Council Laboratories, Fajara, The Gambia

Steve Kaye

Medical Research Council Laboratories, Fajara, The Gambia

Hilton C Whittle

Medical Research Council Laboratories

The Gambia, West Africa.

Antonio Bertoletti

University College Hospital

London

Vasee Moorthy

University of Oxford

**Clinical Monitor**

Tumani Corrah

Medical Research Council Laboratories

The Gambia, West Africa.

**Declaration**

I agree to conduct the study in accordance with the protocol

Samuel McConkey ……………………………… Date …… ……… … … … …

input.doc

**PROTOCOL SYNOPSIS**

| **Title:**  A phase I/II clinical trial to evaluate two administrations of plasmid DNA (pSG2.HBs) and two administrations of modified vaccinia virus Ankara (MVA.HBs) expressing hepatitis B surface antigen combined with lamivudine in Gambian subjects with eAg-positive chronic hepatitis B virus (HBV) infection: HBs004 |
| --- |
| **Funding Agency:**  The Wellcome Trust |
| **Objectives:**  Primary Objectives  1) To assess the safety of the DNA vaccine, pSG2.HBs, and recombinant MVA.HBs in eAg-positive chronic-HBV-infected Gambians  2) To determine the efficacy of a prime-boost immunisation regimen using these vaccines in reducing hepatitis B viral DNA levels and inducing viral clearance either with and without concomitant anti-viral chemotherapy  Secondary Objective  To assess the cellular immune response induced by pSG2.HBs and MVA.HBs in volunteers from Gambia who have eAg-positive chronic Hepatitis B |
| **Study design:**  This is a randomised controlled factorial study of two interventions: prime-boost vaccination and oral lamivudine therapy. Thirty-two subjects with evidence of eAg-positive chronic HBV infection will be divided into four groups. Group E will receive 1 mg plasmid pSG2.HBs by intramuscular injection on two occasions and then 5 x 10^7^ plaque-forming units (pfu) MVA.HBs by intradermal injection on two occasions. Each vaccination will be separated by 3 weeks. Group F will receive lamivudine 100mgs orally daily for 14 weeks. Group G will receive both interventions simultaneously and Group H, the control group, will receive neither. Safety will be assessed by adverse event monitoring, blood chemistry and haematological monitoring of the subjects. Blood samples will also be collected for measurement of HBV DNA by quantitative PCR and assessment of cellular immune response induced in the subjects. |
| **Subject numbers:**  32 subjects |
| **Summary of subject eligibility criteria:**  Healthy subjects aged 15 to 25 with eAg-positive chronic HBV infection |

| **Investigational agents:**  pSG2.HBs  Formulation: 1 mg/mL in phosphate-buffered saline  Vial size: 0.6 mL (0.5 mL intended usable volume); 2 vials per dose  Intended dose: 1 mg  MVA.HBs  Formulation: 5 x 10^8^ pfu/ml in 10 mM Tris, 140 mM NaCl (pH7.7)  Vial size: 0.2 mL (0.1 mL intended usable volume)  Intended dose: 5 x 10^7^ pfu  Lamivudine *Zeffix* (GlaxoSmithKline)  Formulation: 100 mg tablets  Intended dose: 100 mg daily by mouth |
| --- |
| **Endpoints:**  Primary Endpoints:  Toxicity determined by safety assessments.  HBV DNA levels by quantitative PCR  Secondary Endpoint:  Peripheral blood hepatitis B surface antigen-specific CD8+ T cell responses by Interferon-γ Elispot assay and tetramer staining. |

**Table of Contents**

[1. TEAM ROSTER 7](#__RefHeading___Toc7330251)

[2. BACKGROUND AND RATIONALE 8](#__RefHeading___Toc7330252)

[2.1 Introduction 8](#__RefHeading___Toc7330253)

[2.2 Role of cytotoxic T lymphocytes in chronic HBV infection 8](#__RefHeading___Toc7330254)

[2.3 Rationale for the study 9](#__RefHeading___Toc7330255)

[2.4 Investigational agents 11](#__RefHeading___Toc7330256)

[2.4.1 pSG2.HBs 11](#__RefHeading___Toc7330257)

[2.4.2 MVA.HBs 11](#__RefHeading___Toc7330258)

[2.5 Justification of doses and dose interval 11](#__RefHeading___Toc7330259)

[2.6 Preclinical summary 12](#__RefHeading___Toc7330260)

[2.6.1 Toxicology 12](#__RefHeading___Toc7330261)

[2.6.2 Preclinical immunogenicity 12](#__RefHeading___Toc7330262)

[2.6.3 Clinical safety data 13](#__RefHeading___Toc7330263)

[3. OBJECTIVES 13](#__RefHeading___Toc7330264)

[4. STUDY DESIGN 13](#__RefHeading___Toc7330265)

[5. SUBJECT SELECTION 13](#__RefHeading___Toc7330266)

[5.1 Inclusion criteria 14](#__RefHeading___Toc7330267)

[5.2 Exclusion criteria 14](#__RefHeading___Toc7330268)

[6. TRIAL MATERIAL 15](#__RefHeading___Toc7330269)

[6.1 Trial supplies 15](#__RefHeading___Toc7330270)

[6.2 Drug allocation and administration 15](#__RefHeading___Toc7330271)

[7. CONCOMITANT MEDICATION 16](#__RefHeading___Toc7330272)

[8. METHODS OF ASSESSEMENT 16](#__RefHeading___Toc7330273)

[8.1 Clinical monitoring 16](#__RefHeading___Toc7330274)

[8.2 Clinical laboratory tests 17](#__RefHeading___Toc7330275)

[8.2.1 Haematology 17](#__RefHeading___Toc7330276)

[8.2.2 Serum chemistry 17](#__RefHeading___Toc7330277)

[8.3 Cellular immune response assays 17](#__RefHeading___Toc7330278)

[8.4 Analysis of genetic polymorphisms 18](#__RefHeading___Toc7330279)

[8.5 Antibody assays 18](#__RefHeading___Toc7330280)

[8.6 Approximate total blood volumes over 350 days 18](#__RefHeading___Toc7330281)

[8.7 Urinalysis 18](#__RefHeading___Toc7330282)

[8.8 Sample labelling 19](#__RefHeading___Toc7330283)

[8.9 Sample shipment 19](#__RefHeading___Toc7330284)

[9. PROCEDURES 19](#__RefHeading___Toc7330285)

[10. ADVERSE EVENTS 20](#__RefHeading___Toc7330286)

[11. WITHDRAWALS 21](#__RefHeading___Toc7330287)

[12. STATISTICS 21](#__RefHeading___Toc7330288)

[12.1 Design of study 21](#__RefHeading___Toc7330289)

[12.2 Numbers of subjects 21](#__RefHeading___Toc7330290)

[12.3 Randomisation 22](#__RefHeading___Toc7330291)

[12.4 Methods of analysis 22](#__RefHeading___Toc7330292)

[12.5 Justification of open allocation 22](#__RefHeading___Toc7330293)

[12.6 Replacement subjects 22](#__RefHeading___Toc7330294)

[13. RECORDING AND COLLECTION OF DATA 22](#__RefHeading___Toc7330295)

[14. ETHICAL AND REGULATORY REQUIREMENTS 23](#__RefHeading___Toc7330296)

[14.1 Ethical standards 23](#__RefHeading___Toc7330297)

[14.2 Ethical review 23](#__RefHeading___Toc7330298)

[14.3 Informed consent 23](#__RefHeading___Toc7330299)

[14.4 Contact with General Practitioner 23](#__RefHeading___Toc7330300)

[14.5 Regulatory approval 24](#__RefHeading___Toc7330301)

[14.6 Subject Confidentiality 24](#__RefHeading___Toc7330302)

[14.7 Data Protection 24](#__RefHeading___Toc7330303)

[15. LEGAL AND ADMINISTRATIVE OBLIGATIONS 24](#__RefHeading___Toc7330304)

[15.1 Pre-study documentation 24](#__RefHeading___Toc7330305)

[15.2 Insurance 24](#__RefHeading___Toc7330306)

[15.3 Emergency contact cards 25](#__RefHeading___Toc7330307)

[15.4 Monitoring 25](#__RefHeading___Toc7330308)

[15.5 Adherence to the protocol 25](#__RefHeading___Toc7330309)

[15.6 Trial termination 25](#__RefHeading___Toc7330310)

[15.7 Retention of records 25](#__RefHeading___Toc7330311)

[16. AUDIT AND INSPECTION 25](#__RefHeading___Toc7330312)

[17. DISCLOSURE OF INFORMATION 25](#__RefHeading___Toc7330313)

[18. REFERENCES 26](#__RefHeading___Toc7330314)

[APPENDIX I. HANDLING PROCEDURES 29](#__RefHeading___Toc7330315)

[APPENDIX II. COLLECTION OF Peripheral BLood mononuclear cells 30](#__RefHeading___Toc7330316)

[APPENDIX III. FLOW DIAGRAM OF PROTOCOL PROCEDURES 31](#__RefHeading___Toc7330317)

[APPENDIX IV. ADVERSE EVENTS GRADING 32](#__RefHeading___Toc7330318)

# TEAM ROSTER

Principal Investigator

Samuel J McConkey,

Wellcome Tropical Medicine Research Fellow

MRC Laboratories, Fajara,

PO Box 273, Banjul

The Gambia

Tel +220 497381

Fax +220 496 117

[samuel.mcconkey@ndm.ox.ac.uk](mailto:samuel.mcconkey@ndm.ox.ac.uk)

Fellowship Mentor

Adrian VS Hill

Professor, Nuffield D. of Medicine

University of Oxford

Rm 7501, John Radcliffe Hospital

Oxford, OX3 9DU, UK

Tel: +44 (0) 1865 222301

Fax: +44 (0) 1865 221921

[adrian.hill@imm.ox.ac.uk](mailto:adrian.hill@imm.ox.ac.uk)

Co-investigators

Hilton C Whittle

MRC Laboratories

PO Box 273, Banjul,

The Gambia, West Africa.

Tel: +220 497 884

Fax: +220 496 513

hwhittle@mrc.gm

Assan Jaye,

MRC Laboratories, The Gambia

Tel +220 495 442 ext. 428/336/342

Assan_jaye@hotmail.com

Steven Kaye,

Medical Research Council Laboratories, The Gambia

Tel +220 495 442 ext. 342

skaye@mrc.gm

Clinical Monitor

Tumani Correh

MRC Laboratories,

PO Box 273, Banjul,

The Gambia

tcorrah@mrc.gm

Maimuna Mendy,

Medical Research Council Laboratories, The Gambia

Tel +220 495 442 ext. 334

mmendy@mrc.gm

Antonio Bertoletti

Institute of Hepatology

University College of London Medical School

69-75 Chenies Mews

London WC1E 6HX

Fax: +44 (0) 20-7380 0405

Tel: +44 (0) 20-7679 6517

a.bertoletti@ucl.ac.uk

Vasee Moorthy

Medical Research Council

PO Box 273,Banjul,

The Gambia, West Africa

Tel + 220 495 442

vasee.moorthy@ndm.ox.ac.uk

# BACKGROUND AND RATIONALE

## Introduction

Hepatitis B virus (HBV) infection is a leading cause of liver disease world-wide, with approximately a million new cases each year in Europe and 200,000 - 300,000 in the US. Infection rates are probably considerably higher in the endemic regions of Africa and Asia (Maddrey 2000). Between 5 – 90 % of those infected will develop chronic infection, depending on age of infection and therapy. There are more than 350 million chronic carriers of HBV world-wide particularly in sub-Saharan Africa and Asia. In The Gambia 15% of young adults have persistent HBV infection (Ryder *et al*. 1984). Chronic hepatitis leads to cirrhosis, hepatic failure and hepatocellular carcinoma in approximately one quarter of these individuals. HBV infection is therefore a serious public health problem, particularly in endemic areas despite an effective preventive vaccine.

Therapy of chronic hepatitis B infection is still an unmet clinical need. The first effective therapy for chronic HBV infection was treatment with interferon-alpha (IFN-α). The response rate is 5 – 40% (Farrell 2000) and is particularly disappointing in those infected vertically, in Asians, or in those with pre-core mutant HBV, with low alanine aminotransferase (ALT) levels and longstanding infections. This excludes most patients in sub-Saharan Africa. In patients with cirrhosis IFN-α can cause a flare of disease activity leading to liver failure and death. Side effects can be a problem, it is difficult to administer and is expensive.

The antiviral reverse-transcriptase-inhibitor lamivudine is a powerful inhibitor of HBV replication. After 12 weeks of monotherapy the serum levels of HBV-DNA drops by median of 10^5^ logs (Gauthier et al. 1999). It is safe and well tolerated over many years. On stopping therapy most patients revert to the pre-treatment levels of HBV in blood. Therapy for 12 months has been found to induce seroconverstion from HBeAg to anti-e and control of HBV DNA levels in 16-18% of patients compared to 4 –6 % in the control group (Farrell 2000). Patients with low HBV DNA and those with more abnormal ALT levels are more likely to have HBeAg seroconversion on lamivudine therapy. It appears to be equally effective in Asians and Caucasians. A change from methionine to isoleucine or valine in a conserved YMDD motif because of mutation at codon 552 is associated with virologic resistance to lamivudine but this mutant had impaired replication. Several other new antiviral therapies are currently being investigated.

## Role of cytotoxic T lymphocytes in chronic HBV infection

Cytotoxic T lymphocyte (CTL) activity is important in resolving viral infections in animals infected with influenza virus, lymphocytic choriomeningitis virus and mouse cytomegalovirus (Klavinskis *et al* 1989, Lin and Askonas 1981, Reddehease *et al* 1987). In addition, the importance of CTL activity has been shown in the resolution of cytomegalovirus infection in humans (Quinnan *et al* 1982). Several lines of evidence suggest that major histocompatibility complex (MHC) class I-restricted CTL also play a central role in the clearance of HBV infections.

Firstly, patients with acute, self-limited HBV infection usually mount a vigorous and polyclonal cytotoxic T cell response which is targeted against multiple epitopes in the viral nucleocapsid, envelope and polymerase proteins (Bertoletti *et al* 1991, Nayersina *et al* 1993, Rehermann *et al* 1995). In contrast, a vigorous, polyclonal CTL response is not usually readily detectable in the peripheral blood of patients with chronic HBV infection (Barnaba *et al* 1989).

Secondly, a positive clinical response to IFN-α treatment is associated with the induction of an inflammatory response in the liver and appearance of HBV-specific CTL responses (Perrillo 1989). Lamivudine therapy is also associated with increased T cell responses (Boni et al. 1998).

Thirdly, once activated, HBV-specific CTL appear to be able to effect viral elimination, both by direct lysis of infected cells as well as by indirect non-lytic antiviral mechanisms. This is supported by animal models that utilise mice transgenic for the entire HBV genome; in these mice, adoptive transfer of HBV-specific CTL leads to termination of HBV replication in the liver (Guidotti et al 1994, Guidotti et al. 1996). Chimpanzees develop an acute self-limited infection with HBV and they mount a cellular immune response similar to that observed in acutely infected humans. They effect clearance of HBV immunologically through a TNF and IFN-gamma dependent mechanism without widespread hepatocyte death (Guidotti *et al*. 1999).

Finally, patients undergoing spontaneous remission also develop a CTL response to HBV that is similar in strength and specificity (including nucleocapsid, envelope and polymerase responses) to patients who have recovered from acute hepatitis (Rehermann *et al* 1996).

Taken together, these data support the idea that HBV-specific CTL are present in chronically infected patients but that they are quantitatively or qualitatively ineffective with respect to HBV clearance. More importantly, the data also imply that this defect is reversible and that a vaccine capable of inducing HBV-specific CTL responses could represent an effective therapy for chronic HBV infection.

## Rationale for the study

A number of different strategies can be employed to induce CTL responses, including live replicating vectors, nucleic acid immunisation, novel adjuvants, multimeric protein particles, and denatured protein monomers or peptides. Several of these approaches have been applied to the induction of HBV CTL. However, the CTL responses induced have been somewhat disappointing, particularly when the formulations have been administered to humans.

Recombinant viral vectors have been used and developed extensively as antigen delivery systems for over a decade. The induction of hepatitis B surface antigen (HBsAg)-specific and nucleocapsid core (HBcAg)-specific CTL has been demonstrated in mice using vaccinia virus vectors (Ishikawa *et al* 1993). HBV-specific CTL responses have also been demonstrated after immunisation of mice, rhesus macaques and chimpanzees with recombinant retroviral vectors. However, high doses were required, particularly in primates (Townsend *et al* 1997, Sällberg *et al* 1998). Although a variety of attenuated recombinant viral vectors have been developed as antigen delivery systems, not all attenuated viruses are replication-incompetent in mammalian hosts. The use of attenuated but replication-competent viruses can lead to side effects, particularly in immunocompromised individuals.

DNA immunisation strategies are attractive because of their ease of construction and manufacture, and their ability to induce both humoral and cellular immunity. The induction of anti-HBV CTL has been demonstrated in mice following immunisation with DNA plasmids encoding surface (Schirmbeck *et al* 1995) or core antigens (Kuhober *et al* 1996). Human studies of DNA vaccines have been undertaken for influenza, HIV, hepatitis, malaria and various cancers. In general, immune responses were only induced at the highest doses, suggesting that DNA vaccines may be less immunogenic in humans than they are in rodents. To date, no significant side effects have been reported in these studies and valuable safety data are being accumulated. In particular, no evidence of insertion events of plasmid DNA into the host genome have been detected in detailed studies (Nichols *et al* 1995).

Various strategies are being investigated to improve the immunogenicity of DNA vaccines including the use of improved promoter sequences, the addition of immune stimulatory sequences, targeting the antigen to different cellular components, co-administration of cytokine and chemokine encoding plasmids and improved delivery systems. Recent preclinical studies suggest that the response elicited by DNA priming can be substantially enhanced by boosting with the same antigen delivered by a heterologous system, *e.g.,* using a recombinant viral vector (Hanke *et al* 1998, Schneider *et al* 1998). In particular, boosting DNA-primed animals with replication-impaired poxviruses such as modified vaccinia virus Ankara (MVA) induced high levels of protection against malaria (Schneider *et al* 1998) whereas MVA-priming followed by DNA boost was not effective.

MVA is a strain of vaccinia virus that does not replicate in most cell types, including normal human tissues (Mayr *et al* 1978). MVA was derived by 576 passages of a vaccinia virus from a horse pox lesion and was administered to 120,000 people in the last stages of the smallpox eradication programme in Germany. The genome of MVA has been fully sequenced and the virus has six genomic deletions totalling 30kb. As a result of these genomic deletions, MVA has a desirable profile of immune defence genes in contrast to other vaccinia strains (Alcami and Smith 1992, 1995; Symons *et al* 1995; Hirsch *et al* 1996). Recombinant MVA is considered to be a promising human vaccine candidate because of its safety profile and immunogenic properties.

Chronic HBV infection is associated with impaired cellular immunity in the host. This may be due to the high viraemia. The impairment of T cell responses can be restored by lamivudine therapy (Boni 1998).

The aim of this study is to assess the safety, efficacy and immunogenicity of therapeutic heterologous prime-boost vaccination with DNA plasmid vaccine (pSG2.HBs) followed by recombinant MVA vaccine (MVA.HBs) in volunteer subjects with eAg-positive chronic HBV infection. Studies in 10 healthy volunteers in Gambia have shown evidence of cellular immunogenicity and a good safety profile. A study in eAg-negative chronic carriers is in progress. Each vaccine vector will deliver the HBsAg and preS2 antigens. There will be 4 groups of 8 volunteers. Group A will receive the vaccines described above. Group B will receive lamivudine antiviral therapy. Group C will receive both lamivudine and the investigational vaccines and the final control group D will receive no intervention. Previous studies by Vasee Moorthy, Samuel McConkey and Adrian Hill (University of Oxford) in humans using the same DNA and MVA vectors carrying malaria antigens have demonstrated that the vaccines appear to be well tolerated, immunogenic and partly effective in preventing malaria. This is the case also at doses of 2 mg DNA.ME-TRAP and 15 x 10e8 pfu of MVA.ME-TRAP.

## Investigational agents

### pSG2.HBs

Plasmid pSG2.HBs was generated by insertion of a gene fragment containing the *pre-S2* and *S* sequences of HBV strain *ayw* into the polylinker cloning region of vector pSG2. Plasmid pSG2.HBs contains the human cytomegalovirus (hCMV) immediate early promoter with intron A for driving expression of the HBsAg in mammalian cells, followed by the bovine growth hormone transcription termination sequence. The plasmid also contains a kanamycin resistance gene and is capable of replication in *Escherichia coli* but not in mammalian cells.

### MVA.HBs

MVA.HBs contains the gene fragment containing the *pre-S2* and *S* sequences of HBV strain *ayw* inserted into the thymidine kinase locus of MVA. Expression of the HBsAg gene is driven by the vaccinia late/early P7.5 promoter. MVA.HBs also contains the vaccinia late promoter P11 driving expression of the *lacZ* marker gene. MVA.HBs is produced in chicken embryo fibroblast cells.

## Justification of doses and dose interval

The doses to be used and the interval for dosing will be selected based on ongoing studies in humans with related vaccines that use the same vectors and on results from preclinical models.

A previous study of a recombinant DNA vaccine expressing malaria antigens (Adrian Hill, Vasee Moorthy, Samuel McConkey, University of Oxford) used doses of 500 μg and 1mg in Oxford and Gambia. The dose was well tolerated and moderate immune responses were generated (Moorthy, manuscript in preparation). A study of 2 mg DNA vaccine for *Plasmodium falciparum* has been completed, which showed it was well tolerated and suggests that this dose could produce more effective priming than lower doses. Dose escalating studies in humans (Le *et al* 2000, Wang *et al* 1998) evaluated doses of 20, 100 500 and 2500 μg of plasmid DNA; local reactogenicity and systemic symptoms were few and mild. There were no clinically significant biochemical or haematologic changes and no detectable anti-double stranded DNA antibodies were generated. Some CTL responses were observed, particularly in the high dose groups. A DNA vaccine containing 5 mg and 8 mg of DNA has been given by Merck, and was well tolerated (unpublished data). A dose of 1 mg of pSG.HBs will be used in this trial but future studies may evaluate higher doses.

In other clinical trials, Adrian Hill, Vasee Moorthy and Samuel McConkey studied a recombinant MVA vector expressing malaria antigens. The backbone of the MVA vectors for the malaria vaccines is identical to the ones in the proposed study. The doses used were 3 x 10^7^ pfu, 6 x 10^7^ pfu and 15 x 10^7^ pfu. The highest dose of MVA.ME-TRAP has been used in 35 volunteers in Oxford in 4 different studies, and it appears to be well tolerated and highly immunogenic. A higher dose of 5 x 10^8^ pfu MVA vector has been used safely in bovine calves and primates. In humans, the malaria-MVA 5 x 10^7^ pfu was well tolerated and immunogenic. A dose 5 x 10^7^ pfu of MVA.HBs will be used in this study based on safety and immunogenicity results of the on-going studies.

A phase I study of pSG.HBs and MVA.HBs was completed in UK in March 2002. Six healthy subjects will receive each vaccination. The safety results of this study indicate it was well tolerated.

In a pre-clinical study, mice were primed with a DNA vaccine expressing influenza nucleoprotein either nine, six or three days before boosting with a recombinant MVA expressing the same antigen. Primed antigen-specific CD8^+^ T cells remained unresponsive to the boost for up to six days, but were fully boostable after nine days (Schneider *et al* 1999). The heterologous prime-boost strategy for malaria has been tried using intervals of 3, 4 and 8 weeks between the prime and the boosting vaccinations and the shorter intervals appear better, based on measures of cellular immunogenicity. An effective vaccine for Ebola virus in monkeys had an interval of 12 weeks, but in this case antibody responses are probably important for protection (Sullivan *et al.* 2000). A dosing interval of three weeks has therefore been selected for this study. If the on-going malaria clinical trials show increased benefit from a longer interval between prime and boost then this would be considered in this trial.

## Preclinical summary

### Toxicology

Preclinical toxicology was done in mice in preparation for the CTX application. Each vaccine was given by the some route of administration as is anticipated in the clinical trials. MVA.HBs 5 x 10^6^ pfu was administered intradermally into the pina of the ear. Reddening of the ear was noted in about half the mice that received MVA.HBs. Inflammatory changes at the MVA intra-dermal injection site and at the DNA intra-muscular injection site were noted by microscopic pathology done 6 days after vaccination. In addition, mandibular and inguinal lymph nodes showed increased cellularity. By 14 days later, these changes were not detected and there was no evidence of treatment effect after the recovery period.

### Preclinical immunogenicity

Immunogenicity of the product was also confirmed in BALB/c mice by giving 25μg pSG2.HBs at day 0 and MVA.HBs at day 14. Immunogenicity was evaluated at day 28 using 51Cr-release assay and showed > 15% peptide-specific lysis in prime-boosted animals.

### Clinical safety data

To date the pSG.HBs has been given to 6 subjects in UK and 5 subjects in Gambia. It is particularly well tolerated, with no local and few systemic adverse events.

MVA.HBs has been given to 18 subjects in UK and to 5 in Gambia. Transient local redness, hardness of 5 – 15 mm, itch, and scaling were commonly noted. No severe adverse events occurred. There was evidence in the Gambian subjects of activation of the cellular arm of the immune system by the vaccine.

# OBJECTIVES

Primary Objectives

1) To assess the safety of the DNA vaccine, pSG2.HBs, and recombinant MVA.HBs in healthy chronic HBV-infected eAg-positive Gambians.

2) To determine the efficacy of a prime-boost immunisation regimen using these vaccines in reducing hepatitis B viral DNA levels and inducing viral clearance either with and without concomitant anti-viral chemotherapy

Secondary Objective

To assess the cellular immune response induced by pSG2.HBs and MVA.HBs in healthy chronic HBV-infected eAg-positive Gambians.

# STUDY DESIGN

The initial Phase I study will recruit 10 subjects who do not have chronic HBV infection into 2 groups. Five subjects in one group will receive pSG.HBs on two occasions and those in the other will receive MVA.HBs on two occasions. Local and systemic adverse events will be recorded.

The main study is a randomised controlled study. Subjects will be divided into four groups, with 8 subjects in each group. The subjects will be randomised in blocks so that approximately an equal number of subjects with HLA A2 will be in each group to facilitate tetramer assays.

Group E will receive plasmid pSG2.HBs by intramuscular injection on two occasions three weeks apart and MVA.HBs by intradermal injection on two occasions three weeks apart. The first MVA.HBs will be given three weeks after the second pSG2.HBs. Group F will receive lamivudine 100mgs orally daily for 14 weeks. Group G will receive both interventions so that the first vaccine is given after 4 weeks of lamivudine therapy and Group H, the control group, will receive neither. The dose of pSG2.HBs will be 1 mg and the dose of MVA.HBs will be 5 x 10^7^ plaque-forming units (pfu).

Subjects will be required to stay in the unit during dosing and for one hour after dosing. Follow-up will occur 3 monthly for 9 months after vaccination.

# SUBJECT SELECTION

Number of subjects: 32 (in four groups of 8) for the Phase II study

## Inclusion criteria

1. Gambian (from any ethnic group) aged 15 to 25 years inclusive.
2. Chronic HBV infection, defined by presence of HBs for more than 6 months
3. ALT level < twice upper limit of normal value for The Gambia
4. Detectable eAg in the blood
5. Willing to practice effective contraception

## Exclusion criteria

1. Any clinically significant deviation from the normal range in clinical chemistry or haematology variables or any clinically significant deviation from normal in physical examinations, as evaluated by the Investigator, except for the alanine animotransferase (ALT) and aspartate aminotransferase (AST), which may be up to twice the upper limit of the normal range.
2. Any acute illness within two weeks of the start of the study.
3. A history of either significant (as defined by the Investigator) renal, haematological, endocrine, cardiovascular, gastrointestinal, genito-urinary, metabolic, neurological or chronic respiratory disease.
4. Evidence of autoimmune hepatitis, alcoholic liver disease, or liver failure.
5. A history of asthma, allergic skin rash or other allergic conditions, including drug allergies.
6. Allergy to eggs or egg protein.
7. History of drug or alcohol abuse (>30 units/week).
8. History of having taken barbiturates or other drugs affecting liver enzymes within four weeks of the start of the study.
9. A positive test result for HIV.
10. Participation in a drug study within three months of the start of the present study.
11. Previous participation in a vaccine study.
12. Smallpox vaccination

# TRIAL MATERIAL

## Trial supplies

Trial vaccines will be provided by Oxxon Pharmaccines Ltd free of charge.

Lamivudine (Zeffix) will be obtained from GlaxoSmithKline.

pSG2.HBs will be supplied in glass vials containing 0.6 mL (0.5 mL intended usable volume) of plasmid DNA at a concentration of 1 mg/mL in phosphate-buffered saline.

MVA.HBs will be supplied in glass vials containing 0.2 mL (0.1 mL intended usable volume) of MVA.HBs at a concentration of 5 x 10^8^ pfu/mL in 10 mM Tris, 140 mM NaCl, pH 7.7.

The final dose for pSG.HBs will be 1 mg and for MVA.HBs it will be 5 x 10^7^ pfu.

*Treatment pSG2.HBs MVA.HBs Lamivudine*

*group dose dose dose*

Group E Yes, Yes, Yes, Yes none

Group F none none 100 mg daily

Group G Yes, Yes, Yes, Yes 100 mg daily

Group H none none none

Each vaccinated subject will receive 4 vials of pSG2.HBs and 2 vials of MVA.HBs. So 84 vials of pSG.HBs and 42 vials of MVA.HBs will be needed in total. Each vial will be labelled with the company name (Oxxon Pharmaccines Ltd), the name of the product, the concentration, the contents (filled volume), storage details, the manufacturing date and lot number. The outer packaging will include all of the above, plus a warning that the product must be stored out of the reach of children, the statement “For clinical trial use only” and the re-test date.

All study materials will be stored securely in a temperature-controlled facility at MRC laboratories up to the time of administration. The pSG.DNA will be stored at -20ºC +- 3ºC. The MVA.HBs will be stored at - 80ºC +- 5ºC. The investigator or his designated deputy will maintain accurate records demonstrating dates and amounts of drug received, to whom dispensed and accounts of any supplies accidentally or deliberated destroyed; these details will be recorded on the drug accountability forms.

## Drug allocation and administration

Following completion of the screening procedure, each subject will be allocated a study number. Group E will receive pSG2.HBs on day 28 and day 49 and MVA.HBs on day 70 and 91. Group F will receive 100 mg of lamivudine (Zeffix, GSK) for 14 weeks. Group G will receive lamivudine (Zeffix, GSK) for 14 weeks and pSG2.HBs on day 28 and day 49 and MVA.HBs on day 70 and day 91. Group H will receive rabies vaccine on Day 0, 7, and 28.

The pSG2.HBs will be administered as a single deep intramuscular injection. The MVA.HBs will be administered as one- five intradermal injections. During administration of the vaccines, medicines and resuscitation equipment will be immediately available for the management of anaphylaxis. Subjects will stay in the unit for one hour after each vaccination for observation.

In order to minimise dissemination of the recombinant MVA into the environment the inoculation site will be covered with a durable occlusive adherent dressing for two days after immunisation. This should absorb any virus that may leak out through the needle track. Thereafter, it is unlikely that there will be any shedding of virus into the environment as the MVA virus will not replicate. The dressing will be removed by study personnel two days after immunisation. Handling procedures are described in detail in Appendix I.

There are no restrictions on food or lifestyle activity except for illegal drugs, potentially toxic herbs and excessive alcohol usage.

# CONCOMITANT MEDICATION

Non-prescription preparations (*e.g.,* paracetamol) may be given during the study on approval by the Investigator. Any medication taken during the study must be documented on the case report form (CRF) in the appropriate section.

# METHODS OF ASSESSEMENT

## Clinical monitoring

If the subject identifies a general practitioner, they will be contacted after satisfactory screening as notification that the subject is taking part in the study and to ascertain any significant medical history.

On initial screening subjects will be questioned on their past and current health. Concurrent medication will be documented.

Major body systems will be examined and any abnormalities noted. A further physical examination will be performed on day 119.

Subjects will be weighed in light outdoor clothing without shoes and the weight recorded in kilograms. Height (cm) without shoes will be recorded.

Blood pressure will be measured using standard methods. Measurements will be made after the subject has been sitting for at least 2 minutes. Recordings will be made in mmHg. Pulse rate (PR) will be recorded in beats per minute (bpm) at the radial pulse (minimum observation time 1 minute) after the subject has been sitting for 2 minutes. Respiratory rate (RR) will be recorded in breaths per minute (minimum observation time 1 minute) after the subject has been sitting for 2 minutes. The subject’s axillary temperature will be recorded in ºC with the subject sitting (minimum observation time 2 minutes). The above vital sign assessments will be made at screening, day 0 and on days 28, 35, 49, 56, 70, 77, 91, 98, 119, 182, 266, and 350. If at any time vital signs show clinically significant changes then they shall continue to be assessed at 15 minute intervals until no longer indicated (outside ranges BP 90/50 – 150/90 mmHg, PR 40 – 110 bpm, RR 10 – 18/min, temperature 35 – 37.5ºC).

Tolerance and adverse events will be assessed throughout the study. Subjects will be instructed to volunteer adverse events noted at any time during the study.

## Clinical laboratory tests

A copy of the laboratory normal ranges, laboratory accreditation certificates and details of membership of QA schemes will be obtained.

Blood samples will be taken by venepuncture.

### Haematology

The following will be measured:

Haemoglobin

Packed cell volume (PCV)

White cell count

Differential (neutrophils, lymphocytes, monocytes, eosinophils, basophils)

Platelet count

Each assessment will require 1.0 ml of blood.

Subjects in Groups E and G will have 1.0 ml of blood taken in an EDTA tube at screening, and on days 35, 56, 77, 98, 119, 182, 266, and 350. Total = 9mL per subject.

Subjects in Groups F and H will have 1.0 ml of blood taken in an EDTA tube at screening and on days 98, 182, 266 and 350. Total = 5 mL per subject.

### Serum chemistry

The following will be measured:

Aspartate transaminase (AST)

Alanine transferase (ALT)

Creatinine

Gamma glutaryl transpeptidase (GGT)

Each assessment on serum will require 0.5mL of blood.

Subjects in groups E and G will have serum chemistry measured at screening and on days 35, 56, 77, 98, 119, 182, 266, and 350. Total = 4.5 mL per subject.

Subjects in Groups F and H will have serum chemistry measured at screening and on days 98, 182, 266, and 350. Total = 2.5 mL per subject.

## Cellular immune response assays

Induction of cellular immune responses will be assessed by antigen specific T lymphocyte activation measured in an ELISPOT (gamma interferon release) assay, tetramer staining (for HLA A*02 subjects), proliferation and cytotoxicity assay.

In groups E and G blood samples for CTL responses will be taken at screening and on days 56 and 98. Those who show strong specific T cell responses at that time will be asked to provide samples for T cell assays at days 119, 182 and 350. Samples of whole blood collected into sterile heparinised tubes will be processed to isolate peripheral blood mononuclear cells, which will either be analysed fresh or cryopreserved (details are provided in Appendix II).

Each assessment will be done on 20 ml of blood. Total = 60- 120 ml per subject.

In groups F and H blood samples for CTL responses will be taken at screening, and on day 98. If responses are found samples will be taken on days 119, 182 and 350. Total = 40- 100 ml per subject.

## Analysis of genetic polymorphisms

Each subject will have DNA extracted by the salting out method from the cellular pellet of the EDTA sample. The DNA will be stored and archived. Individuals whose DNA is studied will have consented as follows “I understand that a sample of my blood and its genes may be stored and used to study the susceptibility to chronic hepatitis B infection or disease or effectiveness of the hepatitis vaccines,” and this will limit the selection of candidate genes/polymorphisms to be genotyped. Thus, genes to be studies may include, amongst others, the following: HLA class I and II genes, toll-like receptor genes, the TNF and other cytokine genes.

## Antibody assays

After pre-test counselling, an HIV test will be performed at screening.

For all subjects, quantitative PCR will be used to measure HBV DNA levels (Roche, Amplicor). This will be done at screening and day 0, 56, 98, 119, 182, 266 and 350. Serology for HBs, anti-HBs antibody, HbsAg, anti-HBe antibody will be done at the same times in all subjects. Total = 16 ml per subject.

The presence of anti-DNA antibodies will be determined using a standard assay in the Clinical Immunology Department, The Churchill, Oxford Radcliffe Hospital, Oxford. Samples may also be assayed for the presence of anti-MVA antibodies. Excess plasma from the material collected for cellular immune responses as described above will be used for these tests so no additional blood will be collected for these assays.

HLA testing will be performed once for each subject at baseline. Two ml of blood will be taken into an EDTA tube.

## Approximate total blood volumes over 350 days

| Group | E | F | G | H |
| --- | --- | --- | --- | --- |
| Screening for HBV, HIV | 3 | 3 | 3 | 3 |
| Haematology | 9 | 5 | 9 | 5 |
| Serum chemistry | 4.5 | 2.5 | 4.5 | 2.5 |
| CTL assay | 60 | 40 | 60 | 40 |
| HBV PCR and serology | 24 | 24 | 24 | 24 |
| HLA typing | 2 | 2 | 2 | 2 |
| Total ml per subject | 102.5 | 76.5 | 102.5 | 76.5 |

## Urinalysis

Urinalysis will be performed on a freshly voided mid-stream specimen. The following parameters will be measured: pH, protein, glucose and ketones. Sediment will also be examined microscopically for white cells, red cells, bacteria and casts (present or absent).

Urinalysis will be performed at screening and on day 119.

## Sample labelling

Labels will be prepared for all tubes and containers used to collect, store or ship samples. Each label will include the following information:

1. Type of sample
2. Subject number
3. Time of sampling
4. Study number
5. Date of sample

## Sample shipment

Most of the assays will be done in the MRC Laboratories in Fajara, The Gambia. The assays for anti DNA antibodies, and ANA will be shipped to Nuffield Department of Clinical Medicine, University of Oxford. Assays for host genetic polymorphisms will be done in Wellcome Trust Centre for Human Genetics in Oxford, but the samples will be archived and stored in Fajara.

# PROCEDURES

(See Flow Diagram, Appendix III)

1. Subjects will be screened in the eight weeks prior to entering the study. The screen will consist of checking subject eligibility and a full physical examination. The following will be carried out: height, weight, vital signs, haematology, serum chemistry, urinalysis and testing for HBV antibodies, HBsAg, HBeAg and HIV.

1. On each vaccination day subjects will visit the clinical site. The following assessments will be performed pre-dosing: vital signs (20 minutes pre-dose), questioning for adverse events (AEs). Provided all is satisfactory, subjects will receive the first vaccination. Subjects receiving MVA.HBs will have a dressing applied over the injection site. Subjects will remain at the clinical area for one hour following vaccination and will then be allowed to return home.

1. Two days after vaccination subjects will be visited at home by a field worker or they will return to the clinical area. The dressing will be removed from subjects who received MVA.HBs. The injection site will be examined and the subjects will be questioned for AEs.
2. Four days after vaccination subjects will be visited at home by a field worker or they will return to the clinical area. The injection site will be examined and the subjects will be questioned for AEs.
3. Seven days after vaccination subjects will be visited at home by a field worker or they will return to the clinical area. Vital sign assessments will be performed. Blood samples will be taken for haematology, serum chemistry and CTL assays. The injection site will be examined and the subjects will be questioned for AEs.
4. Twenty one days after vaccination subjects will return to the clinical area. Vital sign assessments will be performed. Blood samples will be taken prior to dosing for haematology, serum chemistry and CTL assays. The injection site will be examined and the subjects will be questioned for AEs. Subjects will receive the next vaccination. Subjects who receive MVA.HBs will have a dressing applied over the injection site. Subjects will remain at the clinical site for one hour following vaccination and will then be allowed to return home.
5. Subjects will return to the clinical site on day 119. A full physical examination will be performed and vital signs will be assessed. Blood samples will be taken for haematology, serum chemistry and antibody assays. The injection site will be examined and the subjects will be questioned for AEs.

# ADVERSE EVENTS

Adverse events, however minor, will be recorded as observed by the Investigator or as volunteered by the subject. Full details will be documented in the CRF whether or not the Investigator or his deputies consider the event to be related to the trial substance.

Serious adverse events (SAEs) that occur during the study or within six months of the final vaccination will be notified immediately by telephone to the Ethics Committees that have approved the project.

Serious adverse event are defined as an event that:

1. results in death;
2. is life-threatening (*i.e.,* the subject was at risk of death at the time of the event);
3. requires or prolongs in-patient hospitalisation;
4. results in persistent or significant disability/incapacity;
5. is a congenital anomaly/birth defect;
6. is a cancer.

Minimum details to be given in a telephone report are:

- Name of reporting doctor and contact telephone number.
- Study number.
- Nature of adverse event.
- Subject details (number, initials, sex, date of birth, weight and age).
- Date and time of event.
- Date and time of pSG2.HBs or MVA.HBs administration and dose.
- Rechallenge if applicable.
- Other drug history.
- Other relevant history.
- Outcome.
- Causality.

The event will be documented on the SAE page of the CRF and reported to the Regulatory Authorities as appropriate.

Other adverse events will be graded according to Appendix 4.

If any SAE occurs then that volunteer will not be administered further vaccinations. After the ethics committee’s response to the SAE is received, the Principal Investigator, Clinical Monitor and available co-investigators will meet to determine the future plan for the study, which could involve amending the protocol, discontinuing the vaccinations, or continuing unchanged for the other volunteers.

# WITHDRAWALS

Subjects are free to withdraw from the study at any time without giving a reason. However, the investigator will make an attempt to determine the reason for withdrawal from the study. They may be withdrawn from the study by the supervising physician if any untoward events occur which the Investigator considers detrimental to the well-being of the subject.

Full documentation will be made of any withdrawals that may occur during the study. The Investigator will document the date and time of the withdrawal and the results of any assessments made at this time.

Subjects who have been withdrawn will be monitored at the discretion of the Investigator. They will be replaced as described in section 12.6.

# STATISTICS

## Design of study

This is a randomised, controlled phase II study of the safety, efficacy and immunogenicity of therapeutic prime-boost vaccination for HBV. There will be four groups of subjects in the study: Group E will receive plasmid pSG2.HBs by intramuscular injection on two occasions followed by MVA.HBs by intradermal injection on two occasions. Group F will receive 100 mg of lamivudine daily for 14 weeks. Group G will receive the lamivudine and the same vaccinations as Group E starting after 14 days of lamivudine therapy. Group H will be followed as a control group and receive only rabies vaccination to measure the spontaneous sero-conversion rate.

Most of the clinical results, including adverse event and concomitant medication records will be recorded directly into the Case Report Form. Laboratory reports from the haematology, biochemistry, microbiology and serology laboratories will also be stored and filed as source documents. The original results of HBV DNA quantification and Elispots will be retained. Worksheet from the eAg, anti-core, anti-HBs and HBsAg determinations will also be considered source documents.

## Numbers of subjects

A total of 32 subjects (8 per group) is considered a minimum number in order to meet the study objectives.

There are two primary endpoints of this study. The first is to assess safety of this particular combination of pSG2.HBs and MVA.HBs in Gambians. The second is to determine the efficacy of a prime-boost immunisation regimen using these vaccines in reducing hepatitis B viral DNA levels and inducing viral clearance either with and without concomitant anti-viral chemotherapy. Formal sample sizing calculations have not been performed but it is believed that the sample size of 8 subjects per group will be sufficient for this purpose.

This study will also be used to determine the feasibility of inducing cellular immune responses in subjects using the pSG2.HBs and MVA.HBs vaccines. The proposed numbers of subjects (8 per group) is sufficient for this purpose.

## Randomisation

Block randomisation will be used to ensure that subjects with HLA A*02 are evenly distributed between groups, and that there are at least 2 subjects with HLA A*02 in each of the four groups.

## Methods of analysis

Haematological, serum chemistry, urinalysis parameters and other safety data will be subject to clinical review and tabulation as appropriate. Abnormal laboratory parameter values in data listings will be flagged.

The quantitative HBV DNA assay will be compared using non-parametric methods (Wilcoxon rank sum test) at each time point.

For each subject, the number of gamma-interferon-secreting cells per sample will be determined by ELISPOT assay at each timepoint. Analysis of the cellular immune response will be performed using descriptive statistics.

## Justification of open allocation

No attempt will be made in the study to conceal the allocation group of the subjects either from the subjects themselves, the investigators or laboratory personnel. This is justified given the objective nature of the outcome measures (millimetres of redness at vaccine site, HBV-DNA measure) and the added difficulties that would be encountered in executing a placebo controlled study.

## Replacement subjects

Any subject who withdraws or is withdrawn from the study for reasons considered unconnected with the study medication may be replaced and a new subject allocated to the same treatment. The new subject will complete all study assessments. Safety data for all subjects will be included in the analysis.

# RECORDING AND COLLECTION OF DATA

The investigator will create and use case report forms for the recording and collection of data. All forms will be filled out in black ink or typed. Corrections to data on the CRFs will only be made by crossing out the incorrect data and writing the correct values next to those crossed out. The incorrect data must never be obliterated using such materials as Snopake or Tippex. Each correction will be initialled and dated by the person making the correction. The Investigator or his designated deputy will sign the CRFs to indicate that, to his knowledge, they are complete and correct. If further changes are made after this, the Investigator or his designated deputy will be made aware of the corrections and his awareness documented by initialling and dating the changes.

The Investigator will create and maintain a study file and data management file for the study. All raw data concerning the study will be contained therein. The Investigator will maintain records of drug dispensing, signed consent forms, case report forms, all correspondence and other records which support them. Details of study participation should be entered in the subject’s clinic notes.

# ETHICAL AND REGULATORY REQUIREMENTS

## Ethical standards

This study will be performed in accordance with the current ethical standards for clinical trials in Gambia and United Kingdom.

## Ethical review

The final study protocol will be approved by the Ethics Committee and the Scientific Review Committee at the Medical Research Council Laboratories, Fajara, Gambia. The study will also be approved by the Ethics Committee that has jurisdiction for central Oxfordshire where University of Oxford is located. Written approval of the protocol and informed consent will be obtained prior to recruitment of subjects into the study.

The Investigator will inform, and obtain approval from, the Ethics Committee for all subsequent protocol amendments and amendments to the Information Sheet and Consent Form.

Serious or unexpected adverse events occurring during the trial likely to affect the safety of the subjects or the conduct of the trial will also be reported to the Ethics Committee by the Investigator.

## Informed consent

The Investigator’s will obtain witnessed written informed consent from the subject after adequate explanation of the aims, methods, anticipated benefits and potential hazards of the study before any study medications are administered. For subjects under 18 years written informed consent from the subject’s parents will also be obtained after adequate explanation. The subject will be given a copy of the informed consent documentation. The original copy of the signed and dated informed consent will be retained in the records.

## Contact with General Practitioner

If the subject indicates that they have a Family Doctor or General Practitioner the Investigator will inform the subject’s General Practitioner (GP) that the subject is taking part in the study.

## Regulatory approval

The study will be performed in compliance with the requirements of the Gambian Regulatory authorities.

## Subject Confidentiality

Individual subject medical information obtained as a result of this study will be kept confidential and will not be disclosed to third parties other than those noted below. On the CRFs and other documents, except for the medical chart, and the consent documentation, subjects will be identified by their initials and a subject study number only. The medical chart and the consent documentation will be stored in a secure file.

Medical information may be given to the subject’s personal physician or other appropriate medical personnel responsible for the subject’s welfare.

In compliance with regulatory guidelines regarding the monitoring of clinical studies data generated as a result of the study will be available for inspection, on request, by personnel from regulatory agencies. This shall include all study-relevant documentation (including medical histories), laboratory test results, treatment and diagnostic reports, admission/discharge summaries for hospital admission occurring while the subject is on-study and autopsy reports (if available) for deaths occurring during or in temporal proximity to the study. As part of the required content of informed consent, the subject will be informed that his/her records will be reviewed in this manner.

## Data Protection

All personnel involved in the study will observe or work within the confines of the Data Protection Directive.

# LEGAL AND ADMINISTRATIVE OBLIGATIONS

## Pre-study documentation

The following documents will be assembled at the beginning of the study.

- signed protocol;
- copy of approved Information Sheet and Consent Form;
- copy of the Ethics Committee approval of the protocol and consent form;
- *curricula vitae* of the Principal Investigator and all Co-investigators;
- name and address of the Ethics Committee and membership of the Ethics Committee;
- laboratory normal ranges and documentation of laboratory certification;
- local regulatory approval;

## Insurance

This study is being conducted by the University of Oxford which will have adequate insurance coverage to indemnify the investigators. There is no no-fault compensation scheme in place. The investigators will provide for medical care free of charge for an individual who has an adverse event in the trial to the extent that these are available in The Gambia.

## Emergency contact cards

All subjects will be issued with a study card when they leave the clinic which states that they are participants in a clinical trial. Contact numbers in case of emergency will also be included.

## Monitoring

No provision has been made for monitoring in this trial. The investigator will review the data for completeness and accuracy on 4 occasions during the trial. The investigator will design a database for the results, perform the analysis and present the results.

## Adherence to the protocol

Deviations from the protocol will be avoided as much as possible. Deviations that occur will be documented in a file in the regulatory folder.

The clinic visits may be scheduled three days before or after the planned time at the convenience of the subject and the investigators.

## Trial termination

The trial may be completed as planned, or terminated earlier by the Investigator.

## Retention of records

Study files will be stored securely at the study site whilst the trial is on-going.

# AUDIT AND INSPECTION

ICH guidelines on Good Clinical Practice (GCP) require independent audit of clinical studies. Quality control/assurance may be performed pre-study, during study and post-study. The regulatory authorities in certain countries reserve the right to audit study sites following submission of data in regulatory dossiers. The Investigator understands the procedures that may occur and agrees to give access to the necessary documentation and files.

# DISCLOSURE OF INFORMATION

The information generated as a result of this trial may be presented in verbal or written form at the discretion of the investigator.

# REFERENCES

Alcami A, Smith GL. A soluble receptor for interleukin-1 beta encoded by vaccinia virus: a novel mechanism of virus modulation of the host response to infection. Cell 1992;71:153-167.

Alcami A, Smith GL. Vaccinia, cowpox and camel pox viruses encode soluble gamma-interferon receptors with novel broad species specificity. J Virol 1995;69:4633-4639.

Barnaba V, Franco A, Alberti A, Balsano C, Benvenuto R, Balsano F. Recognition of hepatitis B virus envelope proteins by liver-infiltrating lymphocytes in chronic HBV infection. J Immunol 1989;143:2650-2655.

Bertoletti A, Ferrari C, Fiaccadori F *et al*. HLA class I restricted human cytotoxic T cells recognise endogenously synthesised hepatitis B virus nucleocapsid antigen. Proc Natl Acad Sci USA 1991;88:10445-10449.

Boni C, Bertoletti A, Penna A *et al.* Lamivudine treatment can restore T cell responsiveness in chronic hepatitis B. J Clin Invest 1998: 102: 968-975.

Farrell G. Hepatitis B e Antigen Seroconverstion: Effects of Lamivudine Alone or in combination with Interferon alpha. J Med Virol 2000; 61: 374-379.

Gauthier J, Bourne EJ, Lutz MW, Crowther LM, Dienstag JL, Brown NA, and Condreay LD. Quantitation of hepatitis B viremia and emergence of YMDD variants in patients with chronic hepatitis B treated with lamivudine.

J Infectious Diseases 1999; 180: 1757-1762.

Guidotti LG, Ando K, Hobbs MV *et al*. Cytotoxic T lymphocytes inhibit hepatitis B virus gene expression by a noncytolytic mechanism in transgenic mice. Proc Natl Acad Sci USA 1994;91:3764-3768.

Guidotti LG, Ishikawa T, Hobbs MV, Matzke B, Schreiber R, Chisari FV. Intracellular inactivation of the hepatitis B virus by cytotoxic T lymphocytes. Immunity 1996 Jan;4(1):25-36

Guidotti LG, Rochford R, Chung J, Shapiro M, Purcell R, Chisari FV. Viral clearance without destruction of infected cells during acute HBV infection. Science 1999 284:825-9

Hanke T, Blanchard TJ, Schneider J *et al*. Enhancement of MHC class I-restricted peptide-specific T cell induction by a DNA prime/MVA boost vaccination regimen. Vaccine 1998;16:439-445.

Hirsch VM, Fuerst TR, Sutter G *et al*. Patterns of viral replication correlate with outcome in SIV-infected macaques: effect of prior immunisation with a trivalent SIV vaccine in modified vaccinia virus Ankara. J Virol 1996;70:3741-3752.

Ishikawa T, Kakumu S, Yoshioka K *et al*. Relative immunogenicity of hepatitis B-encoded antigens as targets for cytotoxic T cell response. Immunology 1993;80:313-318.

Klavinskis LS, Whitton JL, Oldstone MBA. Molecularly engineered vaccine which expresses an immunodominant T-cell epitope induces cytotoxic T lymphocytes that confer protection from lethal virus infection. J Virol 1989;63:4311-4316.

Kuhober A, Pudollek H-P, Reifenberg K *et al*. DNA immunisation induces antibody and cytotoxic T cell responses to hepatitis B core antigen in H-2b mice. J Immunol 1996;156:3687-3695.

Lin YL, Askonas BA. Biological properties of an influenza A virus-specific killer T cell clone: inhibition of virus replication *in vivo* and induction of delayed type hypersensitivity reactions. J Exp Med 1981;154:225-234.

*Maddrey WC. Hepatitis B: An important public health issue. J Med Virol 2000; 61: 362-366.

Mayr A, Stickl H, Muller HK, Danner K, Singer H. [The smallpox vaccination strain MVA: marker, genetic structure, experience gained with the parenteral vaccination and behaviour in organisms with a debilitated defence mechanism]. Zentralbl Bakteriol B 1978;167:375-390.

Nayersina R, Fowler P, Guilhot S *et al*. HLA-A2 restricted cytotoxic T lymphocyte response to multiple hepatitis B surface antigen epitopes during hepatitis B virus infection. J Immunol 1993;150:4659-4671.

Nichols WW, Ledwith BJ, Manam SV, Troilo PJ. Potential DNA vaccine integration into host cell genome. Ann New York Acad Sci 1995;772:30-39.

Perrillo RP. Treatment of chronic hepatitis with interferon: experience in Western countries. Semin Liver Dis 1989;9:240-248.

Quinnan GV, Kirmani N Jr, Rook AH *et al*. Cytotoxic T cells in cytomegalovirus infection: HLA-restricted T lymphocyte and non-T-lymphocyte cytotoxic responses correlate with recovery from cytomegalovirus infection in bone marrow transplant recipients. N Engl J Med 1982;307:7-13.

Reddehase MJ, Mutter W, Munch K, Buhring HJ, Koszinowski UH. CD8-positive T lymphocytes specific for murine cytomegalovirus immediate early antigens mediate protective immunity. J Virol 1987;61:3102-3108.

Rehermann B, Fowler P, Sidney J *et al*. The cytotoxic T lymphocyte response to multiple hepatitis B virus polymerase epitopes during and after acute viral hepatitis. J Exp Med 1995;181:1047-1058.

Rehermann B, Lau D, Hoofnagle JH, Chisari FV. Cytotoxic T lymphocyte responsiveness after resolution of chronic hepatitis B virus infection. J Clin Invest 1996;97:1655-1665.

Ryder RW, Whittle HC, Wojiecowsky T, Moffat WM, Baker BA, Sarr E, Oldfield F. Screening for hepatitis B virus markers is not justified in West African transfusion centres. Lancet ii; 449-452 (1984).

Sällberg M, Hughes J, Javadian A *et al*. Genetic immunisation of chimpanzees chronically infected with the hepatitis B virus, using a recombinant retroviral vector encoding the hepatitis B virus core antigen. Hum Gene Ther 1998;9:1719-1729.

Schirmbeck R, Bohm W, Ando K, Chisari FV, Reiman J. Nucleic acid vaccination primes hepatitis-B virus surface antigen-specific cytotoxic T lymphocytes in nonresponder mice. J Virol 1995;69:5929-5934.

Schneider J, Gilbert SC, Blanchard TJ *et al*. Enhanced immunogenicity for CD8^+^ T cell induction and complete protective efficacy of malaria DNA vaccination by boosting with modified vaccinia virus Ankara. Nature Medicine 1998;4:397-402.

Schneider J, Gilbert SC, Hannan CM *et al*. Induction of CD8^+^ T cells using heterologous prime-boost immunisation strategies. Immunol Rev 1999;70:29-38.

Sullivan NJ, Sanchez A, Rollin PE, Yang Z, Nabel GJ. Development of a preventive vaccine for Ebola virus infection in primates. Nature 2000; 408:605- 608.

Symons JA, Alcami A, Smith GL. Vaccinia virus encodes a soluble type I interferon receptor of novel structure and broad species specificity. Cell 1995 81:551-560.

Townsend K, Sällberg M, O’Dea J *et al*. Characterisation of CD8^+^ cytotoxic T lymphocyte responses after genetic immunisation with retrovirus vectors expressing different forms of the hepatitis B virus core and e antigens. J Virol 1997;71:3365-3374.

#### APPENDIX I. HANDLING PROCEDURES

**Storage and handling of the pSG2.HBs and MVA.HBs vaccines**

The vaccine will be stored in the Pharmacy at Medical Research Council Laboratories. Plasmid pSG2.HBs is supplied in glass vials containing 0.6 mL (0.5 mL intended usable volume) of plasmid at a concentration of 1 mg/mL. MVA.HBs is supplied in glass vials containing 200 microlitres of vaccine. The intended usable dose is 100 microlitres (5x10^7^pfu). The vials will be stored in cardboard boxes at –20ºC.

**Location of Immunisations**

Immunisations will take place at Medical Research Council Laboratories, Fajara.

During every administration, adrenaline (1 mg), chlorpheniramine (2.5-5.0 mg) and hydrocortisone (100 mg) in pre-filled syringes and resuscitation equipment will be immediately available for management of anaphylaxis which should be performed as per standard practice.

**Immunisation Procedures and Bandaging**

Gloves and goggles must be worn while handling the vaccines.

MVA.HBs will be drawn up using a 300 microlitre syringe. 100 microlitres of vaccine will be drawn up without allowing air to enter the syringe. It will be administered as a single intradermal injection using the finest gauge needle available.

Plasmid pSG2.HBs will be drawn up with a 1 mL syringe. 500 microlitres will be drawn up from two vials without allowing air to enter the syringe. It will be administered as a single deep intramuscular injection.

The remainder of the vaccine and capped vial will be disposed of into a sealed plastic bag and then into double yellow bags for incineration.

The syringe and needle will be disposed of into a small, specially designated sharps bin, labelled “biohazard”. This should be sealed immediately and placed into a large sharps bin for incineration.

If any product comes out of the skin after injection absorbent material should be used as soon as possible to remove it. The absorbent material should be placed in double yellow bags for incineration.

A dry adhesive dressing will be applied to cover the entire immunisation site. This will remain on the site for two days. The dressing will be removed following two days and disposed of into double yellow plastic bags for incineration. The site will then be cleaned once with an antiseptic. A further dressing may then be applied if desired but no special disposal arrangements are required for this subsequent dressing.

If during the one hour observation period following immunisation it becomes necessary to observe the immunisation site, the initial bandage may be removed and disposed of as above. However, a further bandage should then be applied for the following two days and then disposed of into double yellow bags for incineration.

Any other materials used during the vaccination should be disposed of into double yellow bags for incineration.

On completion of immunisation and bandaging procedures surfaces should be cleaned with 1% Virkon.

If there is any spillage of vaccine, this should be mopped up with liquid Virkon (1%) using absorbent materials which are then disposed of into double yellow bags for incineration.

**Accidental Inoculation**

Any accidental administration (by any route) of vaccine to a person other than the intended vaccinee, *e.g.,* an attending member of staff, should be managed on site as follows. The site of contact with the vaccine should be covered with an adhesive dressing and the individual should be observed as for vaccinees for at least a three hour period with appropriate clinical observations.

**Reporting**

An accident report should be completed.

#### APPENDIX II. COLLECTION OF Peripheral BLood mononuclear cells

SOP for preparation of PBMC is available.

#### APPENDIX III. FLOW DIAGRAM OF PROTOCOL PROCEDURES

| Day | Screen^1^ | 0 | 28 | 30 | 32 | 35 | 49 | 51 | 53 | 56 | 70 | 72 | 74 | 77 | 91 | 93 | 95 | 98 | 119 | 182 | 266 | 350 |
| --- | --- | --- | --- | --- | --- | --- | --- | --- | --- | --- | --- | --- | --- | --- | --- | --- | --- | --- | --- | --- | --- | --- |
| Eligibility, Letter to GP | X |  |  |  |  |  |  |  |  |  |  |  |  |  |  |  |  |  |  |  |  |  |
| Consent documents | X |  |  |  |  |  |  |  |  |  |  |  |  |  |  |  |  |  |  |  |  |  |
| Height and weight | X |  |  |  |  |  |  |  |  |  |  |  |  |  |  |  |  |  | X |  |  |  |
| Physical examination | X |  |  |  |  |  |  |  |  |  |  |  |  |  |  |  |  |  | X |  |  |  |
| Urinalysis | X |  |  |  |  |  |  |  |  |  |  |  |  |  |  |  |  |  | X |  |  |  |
| HLA testing | X |  |  |  |  |  |  |  |  |  |  |  |  |  |  |  |  |  |  |  |  |  |
| HIV serology | X |  |  |  |  |  |  |  |  |  |  |  |  |  |  |  |  |  |  |  |  |  |
| HBV serology | X | X |  |  |  |  |  |  |  | X |  |  |  |  |  |  |  | X | X | X | X | X |
| HBV DNA level | X | X |  |  |  |  |  |  |  | X |  |  |  |  |  |  |  | X | X | X | X | X |
| Gps E & G Vaccination |  |  | X |  |  |  | X |  |  |  | X |  |  |  | X |  |  |  |  |  |  |  |
| Gps F & G Lamivudine |  | X | X | X | X | X | X | X | X | X | X | X | X | X | X | X | X |  |  |  |  |  |
| Vital signs | X | X | X^2^ |  |  | X | X^2^ |  |  | X | X^2^ |  |  | X | X^2^ |  |  | X | X | X | X | X |
| Injection site exam |  |  | X | X | X | X | X | X | X | X | X | X | X | X | X | X | X | X | X | X | X | X |
| Record of medication | X | X | X | X | X | X | X | X | X | X | X | X | X | X | X | X | X | X | X | X | X | X |
| Tolerance/AEs |  |  | X | X | X | X | X | X | X | X | X | X | X | X | X | X | X | X | X | X | X | X |
| GpE&G FBC, Chem | X |  |  |  |  | X |  |  |  | X |  |  |  | X |  |  |  | X | X | X | X | X |
| GpE&G T cell assays | X |  |  |  |  |  |  |  |  | X |  |  |  |  |  |  |  | X | ?^3^ | ?^3^ |  | ?^3^ |
| GpE&G DNA antibody | X |  |  |  |  |  |  |  |  | X |  |  |  |  |  |  |  | X |  | X |  | X |
| GpF&H FBC, Chemistry | X |  |  |  |  |  |  |  |  |  |  |  |  |  |  |  |  | X |  | X | X | X |
| GpF&H T cell assays | X |  |  |  |  |  |  |  |  |  |  |  |  |  |  |  |  | X | ?^3^ | ?^3^ |  | ?^3^ |
| EDTA volume | 5 |  |  |  |  | 2 |  |  |  | 2 |  |  |  | 2 |  |  |  | 2 | 2 | 2 | 2 | 2 |
| LiHeparin volume | 20 |  |  |  |  |  |  |  |  | 20 |  |  |  |  |  |  |  | 20 | 20^3^ | 20^3^ |  | 20^3^ |
| Clotted volume | 5 |  |  |  |  | 3 |  |  |  | 3 |  |  |  | 3 |  |  |  | 3 | 3 | 3 | 3 | 3 |

^1^ Within 8 weeks ^2^ Measured pre-dose ^3^ Samples will be taken if detectable responses are found on day 98

GP general practitioner, HIV Human immunodeficiency virus, AEs adverse events

#### APPENDIX IV. ADVERSE EVENTS GRADING

**1. Definition and Grading Intensity of Adverse Events**

An adverse event is defined as any unintended change in the body structure (signs) or body function (symptoms), whether or not considered related to test product. During the entire study, subjects will be instructed to report all adverse events. All adverse events, whether volunteered, elicited or noted on physical examination, will be recorded throughout the study.

The severity of adverse events will be categorized as follows:

• MILD = Experience that is minor and does not cause significant discomfort to subject or change in activities of daily living (ADLs); subject is aware of symptoms but symptoms are easily tolerated.

• MODERATE = Experience is an inconvenience or concern to the subject and causes interference with ADLs but the subject is able to continue with ADLs.

• SEVERE = Experience significantly interferes with ADLs and the subject is incapacitated and/or unable to continue with ADLs.

SERIOUS = Experience permanent disability, death or admission to hospital for any reason other than management of malaria

**2. Criteria for Determining Relationship to Test Product**

The Investigator will make a determination of the relationship of the adverse event to the test product. The relationship to test product of all adverse events will be classified according to the following guidelines:

• NOT RELATED = Data available to clearly identify an alternative cause of the reaction, e.g., hemorrhage due to mechanical injury.

• UNLIKELY

•• Reasonable temporal relation to vaccination, BUT

•• Unlabeled/unexpected reaction, AND

•• The reaction can be reasonably explained by other factors (such as interventions), AND

•• Negative de-challenge, if available, OR

•• No reasonable temporal relation to vaccination.

• POSSIBLE

•• Reasonable temporal relation to vaccination, AND

•• Labeled/expected reaction, OR

•• Unlabeled/unexpected reaction, BUT

•• Other factors could have caused or contributed to the reaction (such as subject’s clinical state, concomitant therapy, and/or other interventions).

APPENDIX IV continued – Adverse Event Grading

• PROBABLE

•• Reasonable temporal relation to vaccination, AND

•• Labeled/expected reaction, AND

•• The reaction cannot be reasonably explained by other factors (such as the subject’s clinical state, concomitant therapy, and/or other interventions).

• HIGHLY PROBABLE

•• Reasonable temporal relation to vaccination, AND

•• Labeled/expected reaction, AND

•• The reaction cannot be reasonably explained by other factors (such as the subject’s clinical state, concomitant therapy, and/or other interventions), AND

•• Positive de-challenge, if applicable, AND

•• Positive re-challenge, OR

•• Application/vaccination site reaction.

**3. Table for Grading Severity of Adult Adverse Experiences**

ABBREVIATIONS: Abbreviations utilized in this Table include:

ULN =Upper Limit of Normal LLN =Lower Limit of Normal

R_x_ =Therapy Req =Required

Mod =Moderate IV =Intravenous

ADL =Activities of Daily Living Dec =Decreased

ESTIMATING SEVERITY GRADE

For abnormalities NOT found elsewhere in the Toxicity Table use the scale below to estimate grade of severity:

GRADE 1 Mild Transient or mild discomfort (< 48 hours); no medical intervention/therapy required.

GRADE 2 Moderate Mild to moderate limitation in activity – some assistance may be needed; no or minimal medical intervention/therapy required.

GRADE 3 Severe Marked limitation in activity, some assistance usually required; medical intervention/therapy required, hospitalisation possible.

GRADE 4 Serious, Life- threatening Extreme limitation in activity, significant assistance required; significant medical intervention/therapy required, hospitalisation or hospice care probable.

APPENDIX IV continued – Adverse Event Grading

| Parameter | | Grade 1  Mild | | | Grade 2  Moderate | | Grade 3  Severe | Grade 4  Potentially  Life-Threatening | |
| --- | --- | --- | --- | --- | --- | --- | --- | --- | --- |
| LABORATORY | | | | | | | | | |
| LIVER TRANS-  AMINASE (LFTs)  AST (SGOT)  ALT (SGPT) | | 2.0 – 5.0 x ULN  2.0 – 5.0 x ULN | | | >5.0 – 10.0 x ULN  >5.0 – 10.0 x ULN | | >10.0 – 25.0 x ULN  >10.0 – 25.0 x ULN | > 25.0 x ULN  > 25.0 x ULN | |
| RENAL CREATININE | | ------------------- | | | >25 – 50% rise above baseline | | > 50 – 100 % rise above baseline, or permanent rise | >100 % rise above baseline | |
| Parameter | Grade 1  Mild | | | Grade 2  Moderate | | Grade 3  Severe | | | Grade 4  Potentially  Life-Threatening |
| CARDIOVASCULAR | | | | | | | | | |
| Cardiac Arrhythmia | ------------ | | | Asymptomatic; transient dysrhythmia, no R_x_ req | | Recurrent/persistent  dysrhythmia;  symptomatic R_x_ req | | Unstable dysrhythmia,  hospitalization and R_x_ req | |
| Hypertension | Transient, increase >20 mm Hg diastolic BP; no R_x_req | | | Recurrent; chronic increase >20 mm Hg diastolic BP; R_x_ req | | Acute R_x_ req; outpatient OR hospitalization possible | | Hospitalization req OR end organ damage | |
| Hypotension | Transient orthostatic hypotension with heart rate increased by >20 beats/min OR decreased by <10 mm Hg systolic BP, no R_x_ req | | | Symptoms OR BP decreased by <20 mm Hg systolic, correctable with oral fluid R_x_ | | IV fluid req OR hospitalization | | Mean arterial pressure <60 mm Hg, OR end organ damage, OR shock, vasopressor R_x_ req | |
| Pericarditis | Minimal effuision | | | Mild/mod asymptomatic effusion, no R_x_ | | Symptomatic effusion, pain, EKG changes | | Tamponade OR pericardiocentesis OR surgery req | |
| Hemorrhage, blood loss | ------------ | | | Mildly symptomatic, no R_x_ req | | Gross blood loss OR 1-2 units transfused | | Massive blood loss OR >2 units transfused | |
| GASTROINTESTINAL | | | | | | | | | |
| Nausea | Mild OR transient; reasonable intake maintained | | | Mod discomfort OR intake decreased for <3 days | | Severe discomfort OR minimal intake for >3 days | | Hospitalization req | |
| Vomiting | Mild OR transient; 2-3 episodes per day OR mild vomiting lasting <1 week | | | Mod OR persistent; 4-5 episodes per day; OR vomiting lasting > week | | Severe vomiting of all food/fluids in 24 hrs OR orthostatic hypotension OR IV R_x_ req | | Hypotensive shock OR hospitalization req for IV R_x_ req | |
| Diarrhea | Mild OR transient; 3-4 loose stools per day OR mild diarrhea lasting <1 week | | | Mod OR persistent; 5-10 loose stools per day OR diarrhea lasting >1 week | | >10 loose stools/day bloody diarrhea; OR orthostatic hypotension OR electrolyte imbalance, >2 L IV fluid req | | Hypotensive shock OR severe electrolyte imbalance | |
| Oral Discomfort/  Dysphagia | Mild discomfort, no difficulty swallowing | | | Difficulty swallowing but able to eat and drink | | Unable to swallow solids | | Unable to drink fluids; IV fluids req | |
| Constipation | ------------ | | Moderate abdominal pain 78 hours with impaction require outpatient prescription | | | Requiring disimpaction or hospital treatment | | Distention with vomiting OR obstipation | |

APPENDIX IV continued – Adverse Event Grading

| Parameter | Grade 1  Mild | Grade 2  Moderate | Grade 3  Severe | Grade 4  Potentially Life-Threatening |
| --- | --- | --- | --- | --- |
| Bronchospasm Acute | Transient; no R_x_; FEV1 or peak flow reduced to 70% - 80% | R_x_ req; normalizes with bronchodilator; FEV1 or peak flow 50% - 69% | No normalization with bronchodilator; FEV1 or peak flow 25% - 49%, retractions | Cyanosis; FEV1 or peak flow <25% OR intubated |
| Dyspnea | Dyspnea on exertion | Dyspnea with normal activity | Dyspnea at rest | Dyspnea requiring O_2_ therapy |
| NEUROLOGICAL | | | | |
| Neuro-cerebellar | Slight incoordination  OR  Dysdiadochokinesia | Intention tremor OR dysmetria OR slurred speech OR nystagmus | Ataxia requiring assistance to walk or arm incoordination interfering with ADLs | Unable to stand |
| Neuro-psych/mood | --------------- | --------------- | Severe mood changes requiring medical intervention; suicidal ideation | Acute psychosis req hospitalization ; suicidal gesture/attempt |
| Parasthesia (burning, tingling, etc.) | Mild discomfort; no R_x_ req | Mod discomfort; non-narcotic analgesia required | Severe discomfort; OR narcotic analgesia req with symptomatic improvement | Incapacitating; OR not responsive to narcotic analgesia |
| Neuro-motor | Mild weakness in muscle of feet but able to walk and/or mild increase or decrease in reflexes | Mod weakness in feet (unable to walk on heels and/or toes), mild weakness in hands, still able to do most hand tasks and/or loss of previously present reflex or development of hyperreflexia and/or unable to do deep knee bends to weakness | Marked distal weakness (unable to dorsiflex toes or foot drop, and mod proximal weakness e.g., in hands interfering with ADLs and/or requiring assistance to walk and/or unable to rise from chair unassisted | Confined to bed or wheel chair because of muscle weakness |
| Neuro-sensory | Mild impairment (decreased sensation, e.g., vibratory, pinprick, hot/cold in great toes) in focal area or symmetrical distribution | Mod impairment (mod decreased sensation, e.g., vibratory, pinprick, hot/cold to ankles) and/or joint position or mild impairment that is not symmetrical | Severe impairment (decreased or loss of sensation to knees or wrists) or loss of sensation of at least mod degree in multiple different body sites (i.e., upper and lower extremities) | Sensory loss involves limbs and trunk |

APPENDIX IV continued – Adverse Event Grading

| Parameter | | Grade 1  Mild | | Grade 2  Moderate | | Grade 3  Severe | | Grade 4  Potentially  Life-Threatening |
| --- | --- | --- | --- | --- | --- | --- | --- | --- |
| Arthralgia/Arthritis | | Arthralgia | | Arthralgia with joint effusion or moderate impairment of activity | | Frank arthritis with or without effusion OR resulting in severe impairment of activity | | --------------- |
| Myalgia | | Myalgia without limitation of activity | | Muscle tenderness at other than injection site or with moderate impairment of activity | | Frank myonecrosis OR with severe impairment of activity | | --------------- |
| SKIN | | | | | | | | |
| Skin (vaccination site) | | Refer to Appendix 4 for evaluation of specific changes at site of vaccination | | | | | | |
| Skin (general) | | Scattered macular or papular eruption or erythema that is asymptomatic | | Scattered macular or papular eruption or erythema with pruritus or other associated symptoms | | Generalized symptomatic macular, papular, or vesicular eruption | | Exfoliative dermatitis or ulcerating dermatitis |
| URINALYSIS | | | | | | | | |
| Proteinuria:  Random urine | | 1+ | | 2 - 3+ | | 4+ | | Nephrotic syndrome |
| Proteinuria:  24 hour urine | | 200 mg - 1 g loss/day  OR <0.3% OR <3 g/l | | 1 – 2 g loss/day OR  0.3 – 1.0% OR 3 - 10 g/l | | 2 – 3.5 g loss/day OR  >1.0% OR > 10 g/l | | Nephrotic syndrome  OR >3.5 g loss/day |
| Proteinuria:  Hematuria | | Microscopic only <10  RBC/HPF | | >10 RBC/HPF | | Gross, with or without clots OR RBC casts | | Obstructive OR transfusion req |
| MISCELLANEOUS | | | | | | | | |
| Fever  Oral>12 hours | 37.7 - 38.9°C  (100.0 – 101.5°F) | | 39.0 – 39.5°C  (101.6 – 102.9°F)  OR max temp of 103°F | | 39.8 – 40.5°C  (103 - 105°F)  OR max temp of 103.5°F | | >40.5°C (105°F)  OR max temp of >105°F | |
| Headache | Mild, no R_x_ req, OR non-narcotic analgesia R_x_ | | Mod; OR responds to initial narcotic R_x_ | | Severe; intractable; OR requiring repeated narcotic R_x_ | | Requiring hospitalization, associated with neurologic, respiratory or cardiovascular abnormalities | |
| Allergic Reaction | Pruritus without rash at injection site | | Localized urticaria at injection site | | Generalized urticaria angioedema | | Anaphylaxis | |
| ADL | Normal activity reduced <48 hours | | Normal activity reduced 25 - 50% >48 hours | | Normal activity reduced >50%; cannot work >48 hours | | Unable to care for self | |
| Eye |  | | Mild pain, visual changes, conjunctival erythema, abnormal slit lamp | | Loss of vision, clinically diagnosed uveitis, mod-severe pain, glaucoma | | --------------- | |
