# Supplementary material for: Partially Randomized, Non-Blinded Trial of DNA and MVA Therapeutic Vaccines Based on Hepatitis B Virus Surface Protein for Chronic HBV Infection
Source: PLoS One. 2011 Feb 15;6(2):e14626. doi: 10.1371/journal.pone.0014626 (PMC3039644; doi:10.1371/journal.pone.0014626)
Supplement: Protocol S3 — Trial Protocol. (0.27 MB DOC) [file pone.0014626.s010.doc]

University of Oxford

**Clinical Trial Protocol**

**A phase II clinical trial to evaluate two administrations of 2mg plasmid DNA (pSG2.HBs) and one administration of 15 x 10e7 pfu modified vaccinia virus Ankara (MVA.HBs) expressing hepatitis B surface antigen combined with lamivudine in subjects with chronic hepatitis B virus infection: HBs005**

**Protocol No:** HBs005

Original protocol: 19^th^ April 2002

Draft 0_3 22^nd^ May 2003

SCC Draft 1_0 25^th^ May 2003

Version 1.2 after Protocol amendment 3 30th October 2003

This document is the property of the University of Oxford. It may not be used, divulged or published without the consent of University of Oxford.

**This protocol describes an addition of two study arms to an ongoing study**

(SCC 876, L2001-36, L2002-4)

**Funding Agency:** Wellcome Trust

The Wellcome Building 183 Euston Road

London NW1 2BE, United Kingdom

**Principal Investigator:** Samuel J McConkey

MRC Laboratories, The Gambia

**Scientific co-investigators**

Dorka D. Awi

MRC Laboratories, The Gambia

James Cavenaugh

University of Oxford

Adrian VS Hill

University of Oxford

Assan Jaye

MRC Laboratories, The Gambia

Maimuna Mendy

MRC Laboratories, The Gambia

Steve Kaye

MRC Laboratories, The Gambia

Hilton C Whittle

MRC Laboratories, The Gambia.

Antonio Bertoletti

University College London

**Clinical Monitor**

Tumani Corrah

MRC Laboratories

The Gambia, West Africa.

**Declaration**

I agree to conduct the study in accordance with the protocol

Samuel McConkey ……………………………… Date …… ……… … … … …

input.doc

**PROTOCOL SYNOPSIS**

| **Title:**  A phase II clinical trial to evaluate two administrations of 2 mg plasmid DNA (pSG2.HBs) and one administration of 15 x 10e7 pfu modified vaccinia virus Ankara (MVA.HBs) expressing hepatitis B surface antigen combined with lamivudine in subjects with chronic hepatitis B virus (HBV) infection: HBs005 |
| --- |
| **Funding Agency:**  The Wellcome Trust |
| **Objectives:**  Primary Objectives  1) To assess the safety of the DNA vaccine, pSG2.HBs, and recombinant MVA.HBs in chronic-HBV-infected Gambians  2) To determine the efficacy of a prime-boost immunisation regimen using these vaccines in reducing hepatitis B viral DNA levels and inducing viral clearance either with and without concomitant anti-viral chemotherapy  Secondary Objective  To assess the cellular immune response induced by pSG2.HBs and MVA.HBs in volunteers from Gambia who have chronic Hepatitis B |
| **Study design:**  This is a randomised controlled study of two further arms in an ongoing study. If the current vaccines continue to show the excellent safety profile, but less than optimal immunogenicity or efficacy then we will assess the effects of different doses. We will use 2mg DNA and 15 x 10e7 pfu of MVA in a prime-boost vaccination with oral lamivudine therapy. Twenty four subjects with chronic HBV infection will recruited, 12 with eAg in blood (Group I ) and 12 without eAg (Group J). Subjects in both groups will receive 2 mg plasmid pSG2.HBs by intramuscular injection on two occasions and then 15 x 10^7^ plaque-forming units (pfu) MVA.HBs by intradermal injection on one occasion. Subjects in Group I will receive lamivudine 100mg daily PO for 11 weeks. Each vaccination will be separated by 3 weeks. Safety will be assessed by adverse event monitoring, blood chemistry and haematological monitoring of the subjects. Blood samples will also be collected for measurement of HBV DNA by quantitative PCR and assessment of cellular immune response induced in the subjects. |
| **Subject numbers:**  12 subjects with eAg-positive chronic hepatitis B infection  12 eAg-negative subjects |
| **Summary of subject eligibility criteria:**  Healthy subjects aged 15 to 25 with chronic HBV infection |

| **Investigational agents:**  pSG2.HBs  Formulation: 1 mg/mL in phosphate-buffered saline  Vial size: 0.6 mL (0.5 mL intended usable volume); 2 vials per dose  Intended dose: 2 mg  MVA.HBs  Formulation: 5 x 10^8^ pfu/ml in 10 mM Tris, 140 mM NaCl (pH7.7)  Vial size: 0.2 mL (0.1 mL intended usable volume)  Intended dose: 15 x 10^7^ pfu  Lamivudine *Zeffix* (GlaxoSmithKline)  Formulation: 100 mg tablets  Intended dose: 100 mg daily by mouth |
| --- |
| **Endpoints:**  Primary Endpoints:  Toxicity determined by safety assessments.  HBV DNA levels by quantitative PCR  Secondary Endpoints:  Peripheral blood hepatitis B surface antigen-specific CD8+ T cell responses by Interferon-γ Elispot assay and tetramer staining  ‘e’ antigen and surface antigen seroconversion |

**Table of Contents**

[1. TEAM ROSTER 7](#__RefHeading___Toc41673959)

[2. BACKGROUND AND RATIONALE 8](#__RefHeading___Toc41673960)

[2.1 Introduction 8](#__RefHeading___Toc41673961)

[2.2 Role of cytotoxic T lymphocytes in chronic HBV infection 8](#__RefHeading___Toc41673962)

[2.3 Rationale for the study 8](#__RefHeading___Toc41673963)

[2.4 Investigational agents are the same as presently used 9](#__RefHeading___Toc41673964)

[2.5 Justification of doses and dose interval 9](#__RefHeading___Toc41673965)

[2.6 Preclinical summary is as for the main study 10](#__RefHeading___Toc41673966)

[3. OBJECTIVES 10](#__RefHeading___Toc41673967)

[4. STUDY DESIGN 10](#__RefHeading___Toc41673968)

[5. SUBJECT SELECTION 11](#__RefHeading___Toc41673969)

[5.1 Inclusion criteria 11](#__RefHeading___Toc41673970)

[5.2 Exclusion criteria 11](#__RefHeading___Toc41673971)

[6. TRIAL MATERIAL 11](#__RefHeading___Toc41673972)

[6.1 Trial supplies 11](#__RefHeading___Toc41673973)

[6.2 Drug allocation and administration 12](#__RefHeading___Toc41673974)

[7. CONCOMITANT MEDICATION 12](#__RefHeading___Toc41673975)

[8. METHODS OF ASSESSEMENT 12](#__RefHeading___Toc41673976)

[8.1 Clinical monitoring 13](#__RefHeading___Toc41673977)

[8.2 Clinical laboratory tests 13](#__RefHeading___Toc41673978)

[8.2.1 Haematology 13](#__RefHeading___Toc41673979)

[8.2.2 Serum chemistry 13](#__RefHeading___Toc41673980)

[8.3 Cellular immune response assays 13](#__RefHeading___Toc41673981)

[8.4 Analysis of genetic polymorphisms 14](#__RefHeading___Toc41673982)

[8.5 Antibody assays 14](#__RefHeading___Toc41673983)

[8.6 Approximate total blood volumes over 329 days 14](#__RefHeading___Toc41673984)

[8.7 Urinalysis 14](#__RefHeading___Toc41673985)

[8.8 Sample labelling will be as for the other arms 14](#__RefHeading___Toc41673986)

[8.9 Sample shipment will be as for the other arms 15](#__RefHeading___Toc41673987)

[9. PROCEDURES 15](#__RefHeading___Toc41673988)

[10. ADVERSE EVENTS 15](#__RefHeading___Toc41673989)

[11. WITHDRAWALS 15](#__RefHeading___Toc41673990)

[12. STATISTICS 15](#__RefHeading___Toc41673991)

[12.1 Design of study 15](#__RefHeading___Toc41673992)

[12.2 Numbers of subjects 15](#__RefHeading___Toc41673993)

[12.3 Randomisation 15](#__RefHeading___Toc41673994)

[12.4 Methods of analysis 15](#__RefHeading___Toc41673995)

[12.5 Justification of open allocation 15](#__RefHeading___Toc41673996)

[12.6 Replacement subjects 16](#__RefHeading___Toc41673997)

[13. RECORDING AND COLLECTION OF DATA 16](#__RefHeading___Toc41673998)

[14. ETHICAL AND REGULATORY REQUIREMENTS 16](#__RefHeading___Toc41673999)

[14.1 Ethical standards 16](#__RefHeading___Toc41674000)

[14.2 Ethical review 16](#__RefHeading___Toc41674001)

[14.3 Regulatory approval 16](#__RefHeading___Toc41674002)

[14.4 Subject Confidentiality and Data Protection 16](#__RefHeading___Toc41674003)

[15. LEGAL AND ADMINISTRATIVE OBLIGATIONS 16](#__RefHeading___Toc41674004)

[15.1 Emergency contact cards 16](#__RefHeading___Toc41674005)

[15.2 Monitoring 16](#__RefHeading___Toc41674006)

[15.3 Adherence to the protocol 17](#__RefHeading___Toc41674007)

[16. AUDIT AND INSPECTION 17](#__RefHeading___Toc41674008)

[17. DISCLOSURE OF INFORMATION 17](#__RefHeading___Toc41674009)

[18. REFERENCES 18](#__RefHeading___Toc41674010)

[APPENDIX 1: 19](#__RefHeading___Toc41674011)

[APPENDIX II. HANDLING PROCEDURES 19](#__RefHeading___Toc41674012)

[APPENDIX III FLOW DIAGRAM OF PROTOCOL PROCEDURES 21](#__RefHeading___Toc41674013)

# TEAM ROSTER

Principal Investigator

Samuel J McConkey,

Head of Viral Disease Research Programme

MRC Laboratories, Fajara,

PO Box 273, Banjul, The Gambia

Tel +220 497381

Fax +220 496 117

[samuel.mcconkey@ndm.ox.ac.uk](mailto:samuel.mcconkey@ndm.ox.ac.uk)

Co-investigators

Hilton C Whittle

MRC Laboratories

Tel: +220 497 884

Fax: +220 496 513

hwhittle@mrc.gm

Adrian VS Hill

Professor, Nuffield D. of Medicine

University of Oxford

Rm 7501, John Radcliffe Hospital

Oxford, OX3 9DU, UK

Tel: +44 (0) 1865 222301

Fax: +44 (0) 1865 221921

[adrian.hill@imm.ox.ac.uk](mailto:adrian.hill@imm.ox.ac.uk)

Assan Jaye,

MRC Laboratories, The Gambia

Tel +220 495 442 ext. 428/336/342

Assan_jaye@hotmail.com

Steven Kaye,

MRC Laboratories, The Gambia

Tel +220 495 442 ext. 342

skaye@mrc.gm

Clinical Monitor

Tumani Correh

MRC Laboratories,

PO Box 273, Banjul,

The Gambia

tcorrah@mrc.gm

Maimuna Mendy,

MRC Laboratories, The Gambia

Tel +220 495 442 ext. 334

mmendy@mrc.gm

Antonio Bertoletti

Institute of Hepatology

University College of London Medical School

69-75 Chenies Mews

London WC1E 6HX

Fax: +44 (0) 20-7380 0405

Tel: +44 (0) 20-7679 6517

a.bertoletti@ucl.ac.uk

Dorka D. Awi

Medical Research Council

Laboratories, The Gambia

dawi@mrc.gm

James S Cavenaugh

University of Oxford

jcavenaugh@mrc.gm

# BACKGROUND AND RATIONALE

## Introduction

The broad rationale for this extra arm of the ongoing HBs004 trial is the same as for the original trial which indicated the possible need for dose-ranging studies.

Hepatitis B virus (HBV) infection is a leading cause of liver disease world-wide, leading to chronic carriage, cirrhosis and hepatocellular carcinoma. HBV infection is therefore a serious public health problem, particularly in endemic areas despite an effective preventive vaccine. Therapy of chronic hepatitis B infection is still an unmet clinical need. The first effective therapy for chronic HBV infection was treatment with interferon-alpha (IFN-α). The response rate is 5 – 40% (Farrell 2000) and is particularly disappointing in those infected vertically, in Asians, or in those with pre-core mutant HBV, with low alanine aminotransferase (ALT) levels and longstanding infections. This excludes most patients in sub-Saharan Africa. In patients with cirrhosis IFN-α can cause a flare of disease activity leading to liver failure and death. Side effects can be a problem, it is difficult to administer and is expensive.

The antiviral reverse-transcriptase-inhibitor lamivudine is a powerful inhibitor of HBV replication. After 12 weeks of monotherapy the serum levels of HBV-DNA drops by median of 10^5^ logs (Gauthier et al. 1999). It is safe and well tolerated over many years. On stopping therapy most patients revert to the pre-treatment levels of HBV in blood.

## Role of cytotoxic T lymphocytes in chronic HBV infection

Several lines of evidence suggest that major histocompatibility complex (MHC) class I-restricted CTL play a central role in the clearance of HBV infections.

Chimpanzees develop an acute self-limited infection with HBV. They effect clearance of HBV immunologically through a TNF and IFN-gamma dependent mechanism without widespread hepatocyte death (Guidotti *et al*. 1999).

## Rationale for the study

A number of different strategies can be employed to induce CTL responses, including live replicating vectors, nucleic acid immunisation, novel adjuvants, multimeric protein particles, and denatured protein monomers or peptides. Several of these approaches have been applied to the induction of HBV CTL. However, the CTL responses induced have been somewhat disappointing, particularly when the formulations have been administered to humans.

We have found that a prime-boost strategy using DNA and recombinant MVA can induce high levels of T cells (McConkey *et al.* 2003). This was the case mainly at doses of 2mgs of DNA and 15 x 10e7 pfu of MVA. The current proposal is to use these doses of the hepatitis B vaccines.

This strategy is supported by safety data from studies in UK and The Gambia. To date 47 people have received MVA.HBS and 35 have received pSG2.HBs. Studies in 10 healthy volunteers in Gambia have shown a good safety profile for the vaccines under study in this proposal. Also, phase II studies in eAg-negative and eAg-positive chronic carriers currently in progress have shown good safety profile and cellular immune responses although no efficacy at the low doses used. In addition malaria vaccines that have identical vector backbones- the pSG2 plasmid and the MVA parent strain- have been used in UK and The Gambia in more than 200 people. There is an acceptable safety profile also at doses of 2 mg DNA.ME-TRAP and 15 x 10e8 pfu of MVA.ME-TRAP. A summary of the safety data from the recent trail in Farafenni, in which 2mg of pSG2 and 15 x 10e7 pfu of MVA were administered is attached in Appendix 1.

The aim of this study is to assess the safety, efficacy and immunogenicity of therapeutic heterologous prime-boost vaccination with 2 mgs DNA plasmid vaccine (pSG2.HBs) followed by 15 x 10e7 pfu recombinant MVA vaccine (MVA.HBs) in volunteer subjects with chronic HBV infection. Each vaccine vector carries HBsAg and preS2 antigens from the ayw strain of HBV. The current proposal adds two additional arms to the previously approved studies which are underway. We propose two additional groups of twelve volunteers in each - Group I who have eAg-positive chronic hepatitis B and Group J who have eAg-negative chronic hepatitis B. Each volunteer will receive two DNA and one MVA vaccine. Those in Group I will also receive with lamivudine.

## Investigational agents are the same as presently used

## Justification of doses and dose interval

In this proposal we are initiating a change of dose for both the vaccines and a change of schedule. The dose of pSG2.HBs will be 2 mg IM given twice separated by three weeks. And the dose of MVA.HBs will be 15 x 10e7 pfu given only once. The use of these doses is supported by the very favourable safety profile obtained so far with these vaccines in UK and in The Gambia, and by the large quantity of favourable safety data from the very similar vaccines used in the recent large malaria trials done in Farafenni by Dr. Moorthy. The details of this are attached as appendix 1. We propose changing the schedule so that in this arm we will administer only a single MVA.HBs vaccine rather than the two done in the past. The use of a single booster dose of MVA is supported from immunogeniciy results from studies of MVA.ME-TRAP (McConkey et al. 2003). This indicated that there was no additional benefit from the use of a second dose of MVA three weeks after the first. There might be additional benefit if given after a much longer time interval, and this is being explored at present.

Several studies of 2 mg DNA vaccine for *Plasmodium falciparum* have been completed in UK and Gambia, which showed it was well tolerated and suggests that this dose could produce more effective priming than lower doses. Dose escalating studies in humans (Le *et al* 2000, Wang *et al* 1998) evaluated doses of 20, 100 500 and 2500 μg of plasmid DNA; local reactogenicity and systemic symptoms were few and mild. In these studies there were no clinically significant biochemical or haematologic changes and no detectable anti-double stranded DNA antibodies were generated. A DNA vaccine containing 5 mg and 8 mg of DNA has been given by Merck, and was well tolerated (unpublished data).

In other clinical trials, Adrian Hill, Vasee Moorthy and Samuel McConkey studied a recombinant MVA vector expressing malaria antigens. The backbone of the MVA vectors for the malaria vaccines is identical to the ones in the proposed study. The doses used were 3 x 10^7^ pfu, 6 x 10^7^ pfu and 15 x 10^7^ pfu. The highest dose of MVA.ME-TRAP has been used in 35 volunteers in Oxford in 4 different studies, and it appears to be well tolerated and highly immunogenic. A higher dose of 5 x 10^8^ pfu MVA vector has been used safely in bovine calves and primates. In humans, the malaria-MVA 5 x 10^7^ pfu was well tolerated and immunogenic.

Recent studies by Vasee Morthy et al in Gambian adults using 2 doses of 2mg DNA ME-TRAP followed by a single 1.5 x 10^8^ pfu dose of MVA ME-TRAP show that adverse events were rare after the first and second doses of DNA ME-TRAP compared to those in the control group who had rabies vaccine. Injection site pain, limited arm motion, headache and malaise in the first 24 hours after vaccination (mild to moderate in intensity in all but one volunteer) were more common after MVA ME-TRAP vaccination than after the rabies vaccination. Some volunteers developed an injection site blister 1 – 2 days after MVA ME-TRAP vaccination which healed over 1 – 3 weeks without complications. Induration (for 1 – 2 days) and discolouration (faint, shiny macular appearance for several weeks) was common after MVA ME-TRAP vaccination. Results of adverse events are shown in Appendix 1.

There were no significant laboratory safety abnormalities related to vaccination (PCV, ALT and creatinine). A dose of 2mg of pSG.HBs will be used in this trial but future studies may evaluate higher doses. Similarly, a dose 15 x 10^7^ pfu of MVA.HBs will be used in this study.

A phase I study of pSG.HBs and MVA.HBs was completed in UK in March 2002. Six healthy subjects will receive each vaccination. Also, a phase II study of pSG.HBs and MVA.HBs is presently on at the Gambia which was started in 2002. It involved 32 chronic carriers with HbeAg negative and 16 chronic carriers with HbeAg positive. The safety results indicate that it has been well tolerated. It has also shown some good cellular immune responses but no efficacy.

## Preclinical summary is as for the main study

# OBJECTIVES

Primary Objectives

1) To assess the safety of the DNA vaccine, pSG2.HBs, and recombinant MVA.HBs in healthy chronic HBV-infected Gambians.

2) To determine the efficacy of a prime-boost immunisation regimen using these vaccines in reducing hepatitis B viral DNA levels and inducing viral clearance or seroconversion.

Secondary Objective

To assess the cellular immune response induced by pSG2.HBs and MVA.HBs in healthy chronic HBV-infected Gambians.

# STUDY DESIGN

The two additional arms in the trial that are proposed in this protocol will involve twelve chronic HBV carriers who have eAg in their blood (Group I) and twelve chronic HBv carriers who are eAg negative (Group J). Subjects in both groups will receive 2mg DNA and 15 × 10e7 pfu of MVA in a prime-boost vaccination. Subjects in Group I will also receive 11 weeks of oral lamivudine therapy. They will receive a 4 week course of oral lamivudine therapy before receiving the first vaccination, 2mg plasmid pSG2.HBs by intramuscular injection on two occasions and then 15 × 10^7^ plaque-forming units (pfu) MVA.HBs by intradermal injection on one occasion. Each vaccination will be separated by 3 weeks. After the last vaccination subjects will take the lamivudine for another one week and then discontinue it. Subjects will be required to stay in the unit during vaccination and for one hour after vaccination. Follow-up will occur at the following times after vaccination; 30 minutes, 60 minutes, 2 days, 4 days, 7 days, and after the last vaccination follow-up will also be done at 28 days, 3 months, 6 months and 9 months.

# SUBJECT SELECTION

Number of subjects: 12 eAg positive (Group I)

12 eAg negative (Group J)

## Inclusion criteria

1. Gambian (from any ethnic group) aged 15 to 25 years inclusive.
2. Chronic HBV infection, defined by presence of HBs for more than 6 months
3. ALT level < twice upper limit of normal value for The Gambia
4. For Group I detectable eAg in the blood and for Group J no detectable eAg in the blood

## Exclusion criteria

These will be the same as for the other arms in the trail described in protocol HBs004 Version 1.0 of 19^th^ April 2002.

# TRIAL MATERIAL

## Trial supplies

Trial vaccines will be provided by Oxxon Pharmaccines Ltd free of charge.

Lamivudine (Zeffix) will be obtained from GlaxoSmithKline.

pSG2.HBs will be supplied in glass vials containing 0.6 mL (0.5 mL intended usable volume) of plasmid DNA at a concentration of 1 mg/mL in phosphate-buffered saline.

MVA.HBs will be supplied in glass vials containing 0.2 mL (0.1 mL intended usable volume) of MVA.HBs at a concentration of 5 x 10^8^ pfu/mL in 10 mM Tris, 140 mM NaCl, pH 7.7.

The final dose for pSG.HBs will be 2mg and for MVA.HBs it will be 15 ×10e7pfu.

*Treatment pSG2.HBs MVA.HBs Lamivudine*

*group dose dose dose*

Group I Yes, Yes Yes 100 mgs daily

Group J Yes, Yes Yes

Each vaccinated subject will receive 8 vials of pSG2.HBs and 3 vials of MVA.HBs. So 192 vials of pSG.HBs and 72 vials of MVA.HBs will be needed in total. Each vial will be labelled with the company name (Oxxon Pharmaccines Ltd), the name of the product, the concentration, the contents (filled volume), storage details, the manufacturing date and lot number. The outer packaging will include all of the above, plus a warning that the product must be stored out of the reach of children, the statement “For clinical trial use only” and the re-test date.

All study materials will be stored securely in a temperature-controlled facility at MRC laboratories up to the time of administration. The pSG.DNA will be stored at -20ºC +- 3ºC. The MVA.HBs will be stored at - 75ºC +- 8ºC. The investigator or his designated deputy will maintain accurate records demonstrating dates and amounts of drug received, to whom dispensed and accounts of any supplies accidentally or deliberated destroyed; these details will be recorded on the drug accountability forms.

## Drug allocation and administration

Following completion of the screening procedure, each subject will be allocated a study number in the Group I. Each of the subjects will receive oral lamivudine for 4 weeks followed by pSG2.HBs on day 28 and day 49 and MVA.HBs on day 70.

The pSG2.HBs will be administered as a single deep intramuscular injection. The MVA.HBs will be administered as two or three intradermal injections. During administration of the vaccines, medicines and resuscitation equipment will be immediately available for the management of anaphylaxis. Subjects will stay in the unit for one hour after each vaccination for observation.

In order to minimise dissemination of the recombinant MVA into the environment the inoculation site will be covered with a durable occlusive adherent dressing for two days after immunisation. This should absorb any virus that may leak out through the needle track. Thereafter, it is unlikely that there will be any shedding of virus into the environment as the MVA virus will not replicate. The dressing will be removed by study personnel two days after immunisation. Handling procedures are described in detail in Appendix II.

There are no restrictions on food or lifestyle activity except for illegal drugs, potentially toxic herbs and excessive alcohol usage.

# CONCOMITANT MEDICATION

Non-prescription preparations (*e.g.,* paracetamol) may be taken during the study. Any medication taken during the study must be documented on the case report form (CRF) in the appropriate section.

# METHODS OF ASSESSEMENT

## Clinical monitoring

If the subject identifies a general practitioner, they will be contacted after satisfactory screening as notification that the subject is taking part in the study and to ascertain any significant medical history.

On initial screening subjects will be questioned on their past and current health. Concurrent medication will be documented.

Major body systems will be examined and any abnormalities noted.

Subjects will be weighed in light outdoor clothing without shoes and the weight recorded in kilograms. Height (cm) without shoes will be recorded.

Blood pressure will be measured using standard methods. Measurements will be made after the subject has been sitting for at least 2 minutes. Recordings will be made in mmHg. Pulse rate (PR) will be recorded in beats per minute (bpm) at the radial pulse (minimum observation time 1 minute) after the subject has been sitting for 2 minutes. Respiratory rate (RR) will be recorded in breaths per minute (minimum observation time 1 minute) after the subject has been sitting for 2 minutes. The subject’s axillary temperature will be recorded in ºC with the subject sitting (minimum observation time 2 minutes). The above vital sign assessments will be made at screening, day 0 and on days 28, 35, 49, 56, 70, 77, 98, 161, 245, and 329. If at any time vital signs show clinically significant changes then they shall continue to be assessed at 15 minute intervals until no longer indicated (outside ranges BP 90/50 – 150/90 mmHg, PR 40 – 110 bpm, RR 10 – 18/min, temperature 35 – 37.5ºC).

Tolerance and adverse events will be assessed throughout the study. Subjects will be instructed to volunteer adverse events noted at any time during the study.

## Clinical laboratory tests

### Haematology

The following will be measured:

Haemoglobin, Packed cell volume (PCV), White cell count, Differential (neutrophils, lymphocytes, monocytes), Platelet count

Each assessment will require 1.0 ml of blood.

Subjects in Groups I and J will have 2.0 ml of blood taken in an EDTA tube at screening, and on days 35, 56, 77, 98, 161, 245, and 329. Total = 16.0 mL per subject.

### Serum chemistry

The following will be measured:

Aspartate transaminase (AST), Alanine transferase (ALT),Creatinine, Gamma glutaryl transpeptidase (GGT)

Each assessment on serum will require 0.5mL of blood.

Subjects in group I will have serum chemistry measured at screening and on days 35, 56, 77, 98, 161, 245, and 329. Total = 4.0 mL per subject.

## Cellular immune response assays

Induction of cellular immune responses will be assessed by antigen specific T lymphocyte activation measured in an ELISPOT (gamma interferon release) assay on fresh cells and in selected cases by tetramer staining (for HLA A*02 subjects), proliferation and cytotoxicity assay.

Blood samples for CTL responses will be taken at screening and on days 56, 77, 98, 161, 245 and 329. Samples of whole blood collected into sterile heparinised tubes will be processed to isolate peripheral blood mononuclear cells, which will either be analysed fresh or cryopreserved. The cryopreserved cells will be used to characterise the cellular response in detail using intracellular cytokine staining and multichannel FACS counting, tetramers staining, proliferation and cytokine measurement.

Each assessment will be done on 20 ml of blood. Total = 140 ml per subject.

## Analysis of genetic polymorphisms

This will be the same as for the existing arms of the trail described in Protocol HBs004 Version 1.0 dated 19^th^ April 2002.

## Antibody assays

After pre-test counselling, an HIV test will be performed at screening.

For all subjects, quantitative PCR will be used to measure HBV DNA levels (Roche, Amplicor). This will be done at screening and day 0, 56, 77, 98, 161, 245, and 329. Serology for HBs, anti-HBs antibody, HbsAg, anti-HBe antibody will be done at the same times in all subjects. Total = 16 ml per subject.

The presence of anti-DNA antibodies will be determined using a standard assay in the Clinical Immunology Department, The Churchill, Oxford Radcliffe Hospital, Oxford. Samples may also be assayed for the presence of anti-MVA antibodies. Excess plasma from the material collected for cellular immune responses as described above will be used for these tests so no additional blood will be collected for these assays.

HLA testing will be performed once for each subject at baseline. Two ml of blood will be taken into an EDTA tube.

## Approximate total blood volumes over 329 days

| Group | I | J |
| --- | --- | --- |
| Screening for HBV, HIV | 3 | 3 |
| Haematology | 16 | 16 |
| Serum chemistry | 4 | 4 |
| CTL assay | 140 | 140 |
| HBV PCR and serology | 24 | 24 |
| HLA typing | 2 | 2 |
| Total ml per subject | 189 | 189 |

## Urinalysis

Urinalysis will be performed at screening.

## Sample labelling will be as for the other arms

## Sample shipment will be as for the other arms

# PROCEDURES

(See Flow Diagram, Appendix III)

During the intervention phase these will be the same as for the other arms of the study

After the intervention phase subjects will return to the clinical site on study days 98, 161, 245, and 329. A full physical examination will be performed and vital signs will be assessed. Blood samples will be taken for haematology, serum chemistry and antibody assays. The injection site will be examined and the subjects will be questioned for AEs.

# ADVERSE EVENTS

These will be recorded and graded in the same way as for the other arms in the study.

# WITHDRAWALS

Management of subjects who withdraw from the study will be as for the other arms.

# STATISTICS

## Design of study

This is a phase II study of the safety, efficacy and immunogenicity of therapeutic prime-boost vaccination for HBV. This adds two arms to an existing study of the same vaccines; one arm with eAg positive subjects and one with eAg-negative subjects. The addition of these arms makes this a dose-ranging study.

## Numbers of subjects

Twelve subjects in each group are considered a minimum number in order to meet the study objectives. This has been increased from 8 subjects per group in the previous arms so that it is likely that data from at least 10 subjects will be available at the end of the study.

There are two primary endpoints of this study. The first is to assess safety of this particular combination of pSG2.HBs and MVA.HBs in Gambians. The second is to determine the efficacy of a prime-boost immunisation regimen using these vaccines in reducing hepatitis B viral DNA levels and inducing viral clearance.

## Randomisation

No randomisation will be done to select the subjects are the eligibility criteria for the two groups are different.

## Methods of analysis

This will be the same as for the other arms.

## Justification of open allocation

No attempt will be made in the study to conceal the allocation group of the subjects either from the subjects themselves or the investigators. This is justified given the objective nature of the outcome measures (millimetres of redness at vaccine site, HBV-DNA measure) and the added difficulties that would be encountered in executing a placebo controlled study.

## Replacement subjects

Any subject who withdraws or is withdrawn from the study for reasons considered unconnected with the study medication may be replaced and a new subject allocated to the same treatment. The new subject will complete all study assessments. Safety data for all subjects will be included in the analysis.

# RECORDING AND COLLECTION OF DATA

This will be done in the same way as for subjects in the other arms of the trial.

# ETHICAL AND REGULATORY REQUIREMENTS

## Ethical standards

This study will be performed in accordance with the current ethical standards for clinical trials in Gambia.

## Ethical review

The final study protocol will be approved by the Ethics Committee and the Scientific Review Committee at the Medical Research Council Laboratories, Fajara, Gambia. The study will also be approved by the Ethics Committee that has jurisdiction for central Oxfordshire where University of Oxford is located. Written approval of the protocol and informed consent will be obtained prior to recruitment of subjects into the study.

## Regulatory approval

The study will be performed in compliance with the requirements of the Gambian authorities.

## Subject Confidentiality and Data Protection

These aspects will be handled in the same way as the other arms of the study.

# LEGAL AND ADMINISTRATIVE OBLIGATIONS

## Emergency contact cards

All subjects will be issued with a study card when they leave the clinic which states that they are participants in a clinical trial. Contact numbers in case of emergency will also be included.

## Monitoring

No provision has been made for monitoring in this trial.

## Adherence to the protocol

Deviations from the protocol will be avoided as much as possible. Deviations that occur will be documented in a file in the regulatory folder.

The clinic visits may be scheduled up to seven days before or after the planned time at the convenience of the subject and the investigators.

# AUDIT AND INSPECTION

ICH guidelines on Good Clinical Practice (GCP) require independent audit of clinical studies. Quality control/assurance may be performed pre-study, during study and post-study. The regulatory authorities in certain countries reserve the right to audit study sites following submission of data in regulatory dossiers. The Investigator understands the procedures that may occur and agrees to give access to the necessary documentation and files.

# DISCLOSURE OF INFORMATION

The information generated as a result of this trial may be presented in verbal or written form at the discretion of the investigator.

# REFERENCES

Barnaba V, Franco A, Alberti A, Balsano C, Benvenuto R, Balsano F. Recognition of hepatitis B virus envelope proteins by liver-infiltrating lymphocytes in chronic HBV infection. J Immunol 1989;143:2650-2655.

Farrell G. Hepatitis B e Antigen Seroconverstion: Effects of Lamivudine Alone or in combination with Interferon alpha. J Med Virol 2000; 61: 374-379.

Gauthier J, *et al*. Quantitation of hepatitis B viremia and emergence of YMDD variants in patients with chronic hepatitis B treated with lamivudine.

J Infectious Diseases 1999; 180: 1757-1762.

Guidotti LG, Rochford R, Chung J, Shapiro M, Purcell R, Chisari FV. Viral clearance without destruction of infected cells during acute HBV infection. Science 1999 284:825-9

Lin YL, Askonas BA. Biological properties of an influenza A virus-specific killer T cell clone: inhibition of virus replication *in vivo* and induction of delayed type hypersensitivity reactions. J Exp Med 1981;154:225-234.

McConkey SJ, Reece WR, Moorthy V *et al*. Enhanced T-cell immunogenicity of plasmid DNA vaccines boosted by recombinant modified vaccinia virus Ankara in humans. Nature Medicine 25 May 2003, doi:10.1038/nm881

Quinnan GV, Kirmani N Jr, Rook AH *et al*. Cytotoxic T cells in cytomegalovirus infection: HLA-restricted T lymphocyte and non-T-lymphocyte cytotoxic responses correlate with recovery from cytomegalovirus infection in bone marrow transplant recipients. N Engl J Med 1982;307:7-13.

Reddehase MJ, Mutter W, Munch K, Buhring HJ, Koszinowski UH. CD8-positive T lymphocytes specific for murine cytomegalovirus immediate early antigens mediate protective immunity. J Virol 1987;61:3102-3108.

Ryder RW, Whittle HC, Wojiecowsky T, Moffat WM, Baker BA, Sarr E, Oldfield F. Screening for hepatitis B virus markers is not justified in West African transfusion centres. Lancet ii; 449-452 (1984).

Sällberg M, Hughes J, Javadian A *et al*. Genetic immunisation of chimpanzees chronically infected with the hepatitis B virus, using a recombinant retroviral vector encoding the hepatitis B virus core antigen. Hum Gene Ther 1998;9:1719-1729.

Schneider J, Gilbert SC, Blanchard TJ *et al*. Enhanced immunogenicity for CD8^+^ T cell induction and complete protective efficacy of malaria DNA vaccination by boosting with MVA Nature Medicine 1998;4:397-402.

Schneider J, Gilbert SC, Hannan CM *et al*. Induction of CD8^+^ T cells using heterologous prime-boost immunisation strategies. Immunol Rev 1999;70:29-38.

#### APPENDIX 1:

***Preliminary safety results of a trial in Farafenni in The Gambia of 15 x 10e7 pfu MVA.ME-TRAP. The table illustrates frequency of solicited symptoms during the 7 days after MVA vaccine or after HDC Rabies vaccine.***

|  | **i.d. MVA ME-TRAP** | | | | | **i.d. RABIES VACCINE** | | | | |
| --- | --- | --- | --- | --- | --- | --- | --- | --- | --- | --- |
|  | **N** | **%** | | | **95% CI** | **N** | | **%** | | **95% CI** |
| **Local symptoms** | | | | | | | | | | |
| Limited arm motion | 28/152 | | 18% | | (13-26) | | 2/158 | | 1.3% | (0.2-4.5) |
| Pain | 103/152 | | 68% | | (60-75) | | 19/157 | | 12% | (7.4-18) |
| Discolouration | 116/151 | | 77% | | (69-83) | | 26/156 | | 17% | (11-23) |
| Induration | 138/152 | | 91% | | (85-94) | | 86/158 | | 54% | (46-62) |
| Blister | 89/151 | | 59% | | (51-67) | | 14/156 | | 9.0% | (5.0-15) |
| **General symptoms** | | | | | | | | | | |
| Headache | 45/161 | | 28% | (21-36) | | | 19/168 | | 11% | (6.9-17) |
| Objective Fever | 6/161 | | 3.7% | (1.4-7.9) | | | 0/168 | | 0% | (0-2.0) |
| Malaise | 40/161 | | 25% | (18-32) | | | 14/168 | | 8.3% | (4.6-14) |
| Nausea | 3/161 | | 1.9% | (0.4-5.3) | | | 1/168 | | 0.6% | (0-3.3) |

Notes: N = total number of subjects who received the third dose. n (%) = number (percentage) of subjects who had at least one report of the symptom.

The adverse events noted above were almost all grade 1 or grade2. There were no serious adverse events. Three subjects in this trial had grade 3 adverse events which occurred soon after the MVA.ME-TRAP vaccination. These are described briefly below.

Subject 92: grade 3 pain both arms, and grade 3 limited arm motion both arms, both on day 1

Subject 284: grade 3 headache, malaise, and pain left and right, all on day 1

Subject 311: grade 3 limited arm motion both arms, day 1 and day 2.

In each case these resolved completely.

#### APPENDIX II. HANDLING PROCEDURES

**These will be the same as for the other arms of the trial**

#### APPENDIX III FLOW DIAGRAM OF PROTOCOL PROCEDURES

| Day | Screen^1^ | 0 | 28 | 30 | 32 | 35 | 49 | 51 | 53 | 56 | 70 | 72 | 74 | 77 | 98 | 161 | 245 | 329 |
| --- | --- | --- | --- | --- | --- | --- | --- | --- | --- | --- | --- | --- | --- | --- | --- | --- | --- | --- |
| Eligibility | X |  |  |  |  |  |  |  |  |  |  |  |  |  |  |  |  |  |
| Consent documents | X |  |  |  |  |  |  |  |  |  |  |  |  |  |  |  |  |  |
| Height and weight | X |  |  |  |  |  |  |  |  |  |  |  |  | X |  |  |  |  |
| Physical examination | X |  |  |  |  |  |  |  |  |  |  |  |  | X |  |  |  |  |
| Urinalysis | X |  |  |  |  |  |  |  |  |  |  |  |  |  |  |  |  |  |
| HLA testing | X |  |  |  |  |  |  |  |  |  |  |  |  |  |  |  |  |  |
| HIV serology | X |  |  |  |  |  |  |  |  |  |  |  |  |  |  |  |  |  |
| HBV serology | X | X |  |  |  |  |  |  |  | X |  |  |  | X | X | X | X | X |
| HBV DNA level | X | X |  |  |  |  |  |  |  | X |  |  |  | X | X | X | X | X |
| Vaccination |  |  | X |  |  |  | X |  |  |  | X |  |  |  |  |  |  |  |
| Lamivudine supply |  | X | X | X | X | X | X | X | X | X | X | X | X |  |  |  |  |  |
| Vital signs | X | X | X^2^ |  |  | X | X^2^ |  |  | X | X^2^ |  |  | X | X | X | X | X |
| Injection site exam |  |  | X | X | X | X | X | X | X | X | X | X | X | X | X | X | X | X |
| Record of medication | X | X | X | X | X | X | X | X | X | X | X | X | X | X | X | X | X | X |
| Tolerance/AEs |  |  | X | X | X | X | X | X | X | X | X | X | X | X | X | X | X | X |
| FBC, Chem | X |  |  |  |  | X |  |  |  | X |  |  |  | X | X | X | X | X |
| T cell assays | X |  |  |  |  |  |  |  |  | X |  |  |  | X | X | X | X | X |
| DNA antibody | X |  |  |  |  |  |  |  |  | X |  |  |  | X |  | X |  | X |
| EDTA volume | 5 |  |  |  |  | 2 |  |  |  | 2 |  |  |  | 2 | 2 | 2 | 2 | 2 |
| LiHeparin volume | 20 |  |  |  |  |  |  |  |  | 20 |  |  |  | 20 | 20 | 20 | 20 | 20 |
| Clotted volume | 5 | 3 |  |  |  | 3 |  |  |  | 3 |  |  |  | 3 | 3 | 3 | 3 | 3 |

^1^ Within 12 weeks ^2^ Measured pre-dose

HIV Human immunodeficiency virus, AEs adverse events
